# Leveraging Metal and Ligand Reactive Sites for One Pot Reactions: Ligand‐Centered Borenium Ions for Tandem Catalysis with Palladium

**DOI:** 10.1002/chem.202201791

**Published:** 2022-09-26

**Authors:** Manisha Skaria, Johnathan D. Culpepper, Scott R. Daly

**Affiliations:** ^1^ Department of Chemistry The University of Iowa Iowa City Iowa 52242 USA

**Keywords:** borenium, boron ligand, cross coupling, cycloaddition, tandem catalysis

## Abstract

Tandem catalysts that perform two different organic transformations in a single pot are highly desirable because they enable rapid and efficient assembly of simple organic building blocks into more complex molecules. Many examples of tandem catalysis rely on metal‐catalyzed reactions involving one or more metal complexes. Remarkably, despite surging interest in the development of chemically reactive (i. e., non‐innocent) ligands, there are few examples of metal complexes that leverage ligand‐centered reactivity to perform catalytic reactions in tandem with separate catalytic reactions at the metal. Here we report how multifunctional Pd complexes with triaminoborane‐derived diphosphorus ligands, called TBDPhos, appear to facilitate borenium‐catalyzed cycloaddition reactions at the ligand, and Pd‐catalyzed Stille and Suzuki cross‐coupling reactions at the metal. Both transformations can be accessed in one pot to afford rare examples of tandem catalysis using separate metal and ligand catalysis sites in a single complex.

## Graphical abstract

**Pd complexes** with triaminoborane‐containing diphosphorous ligands are reported that are capable of tandem cycloaddition and cross‐coupling catalysis using separate reactive sites on the metal and ligand.

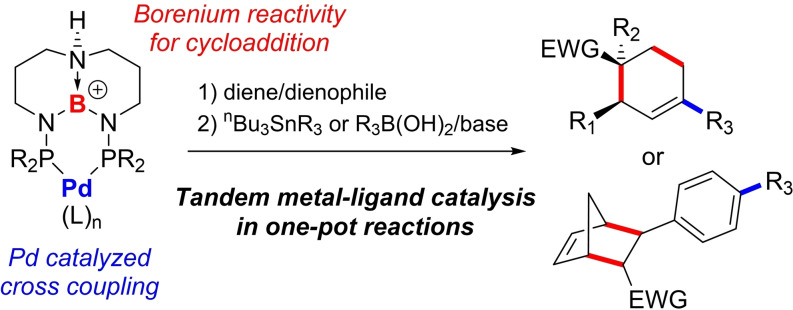

## Introduction

Catalysts that promote atom‐efficient, multistep assembly of organic molecules in one‐pot reactions are highly attractive for synthesis of complex molecular scaffolds used in pharmaceuticals, agrochemicals, and other fine chemicals.^[1]^ One‐pot reactions can collapse the synthesis timeline, limit material loss, and reduce operational costs because they do not require workup and purification after each reaction step. These reactions are also more amenable to automation because of their improved operational simplicity.^[2]^

A favored method of one‐pot reactions is tandem catalysis: coupled catalytic reactions that sequentially transform a substrate via two or more mechanistically distinct processes.^[3]^ Tandem catalysis can occur by combining multiple catalysts in a single reaction vessel to drive sequential catalytic transformations (orthogonal tandem catalysis) or through use of a single catalyst (assisted or auto‐tandem catalysis).^[3a]^

Despite the existence of many chemically reactive ligands that can work independently or cooperatively with metals to perform a single reaction (e. g., metal‐ligand cooperativity and bifunctional catalysis),^[4]^ we are not aware of well‐defined molecular complexes capable of performing two different catalytic transformations using separate metal and ligand reactive sites. Most examples of tandem catalysis rely exclusively on reactions that are catalyzed by the metals of one or more homogenous catalysts. An alternative approach would be the use of multifunctional metal catalysts that have active sites on the ligand capable of orthogonal reactivity.

**Figure 1:**
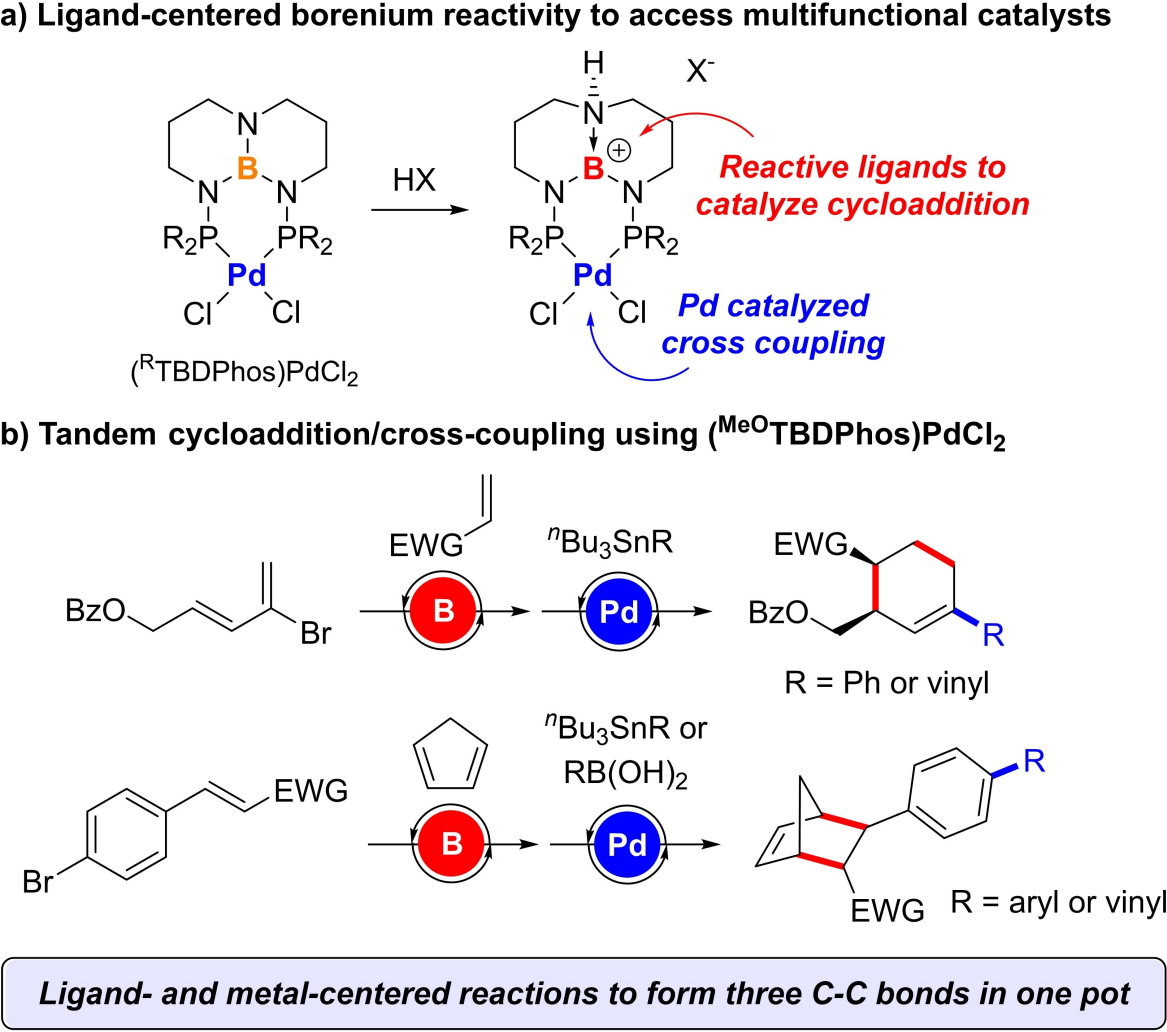
a) Structure and borenium reactivity of (^R^TBDPhos)PdCl_2_ complexes. b) Summary of tandem catalysis results described in this report.

**Scheme 1:**
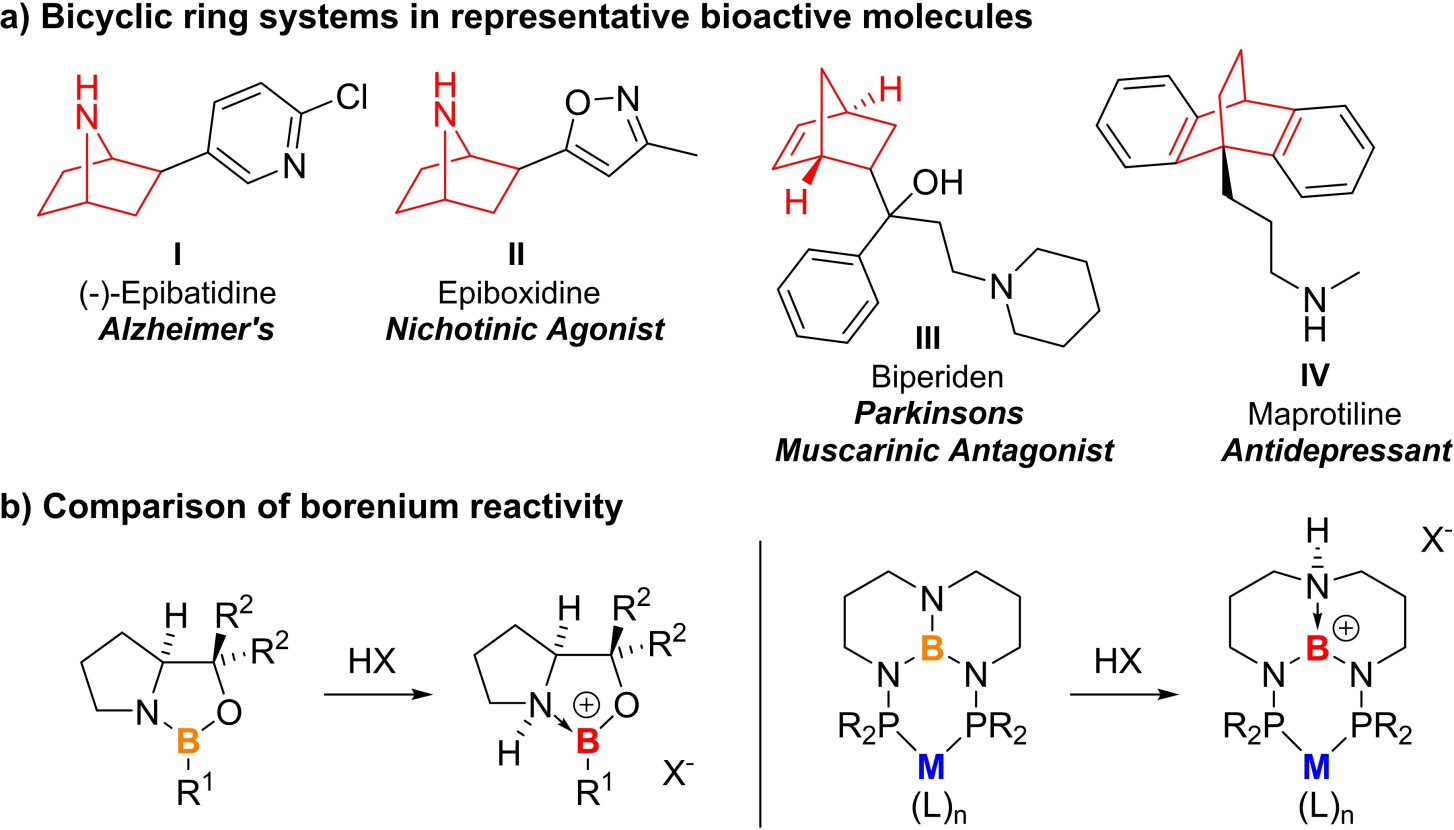
a) Representative bio‐active molecules with bicyclic ring systems. b) Comparison of oxazaborolidine and borenium reactivity in metal TBDPhos complexes.

Here we describe how Pd complexes with a class of chemically reactive boron ligands called TBDPhos are capable of catalyzing two of the most important C−C forming reactions, cycloaddition and cross coupling, in tandem, one‐pot reactions (Figure 1). As elaborated by Baran and others,^[5]^ these complementary reactions are especially powerful when used together. Cycloaddition reactions rapidly build complexity by generating 2D and 3D (bicyclic) ring systems and stereocenters (Scheme 1a), whereas cross coupling is highly modular with large libraries of substrates available for rapid and diverse functionalization. Our results show how both reactions can be catalyzed using separate metal‐ and ligand‐centered reactive sites in a single multifunctional catalyst.^[6]^

## Results and Discussion

To investigate the feasibility of tandem metal‐ligand catalysis, we focused our initial studies on (^R^TBDPhos)PdCl_2_ complexes. ^R^TBDPhos is a class of diphosphorus ligands (where R is the substituent attached to phosphorus) that contain a chemically reactive triaminoborane derived from triazaboradecalin (TBD).^[7]^ We have shown in previous work how the bridgehead N−B bond on the TBD backbone can undergo trans addition of H−X (X=OH, OR, Cl, and F) to form new B−X and N−H bonds while ^R^TBDPhos is bound to metals.^[8]^ This reactivity proceeds via formation of highly Lewis acidic borenium ions (three‐coordinate boron cations)^[9]^ that form by protonation of the bridgehead nitrogen on TBD (Scheme 1b). We have shown that the ligand‐centered borenium ion can be isolated by using acids with weakly coordinating anions like bistriflimide (NTf_2_^−^).^[8b]^

Borenium ions like those generated in ^R^TBDPhos complexes are strong Lewis acids that are capable of catalyzing cycloaddition reactions. For example, it has been shown that treating oxazaborolidines with strong Bronsted acids like HOTf or HNTf_2_ yielded borenium ions that catalyzed a wide range of Diels–Alder (DA) reactions (Scheme 1b).^[10]^ We envisioned that ligand‐centered borenium reactivity in ^R^TBDPhos complexes could be used similarly for catalysis at the ligand, thereby leaving the metal available for other transformations.

**Scheme 2:**
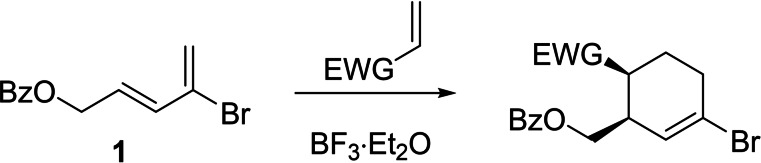
General BF_3_ ⋅ Et_2_O catalyzed DA reactions reported by Brimble and Furkert using the brominated diene **1**.^[11]^

To test (^R^TBDPhos)PdCl_2_ complexes as multifunctional catalysts, we pursued synthetic targets that could be formed via Lewis‐acid catalysed cycloaddition and Pd‐catalysed cross‐coupling reactions in separate steps. Brimble, Furkert, and co‐workers recently described such stepwise reactions starting with Lewis acid catalysed cycloaddition of the 2‐bromo‐1,3‐butadiene **1** using BF_3_ ⋅ Et_2_O (Scheme 2).^[11]^ After workup and isolation, the brominated cycloaddition products were then functionalized with Stille and Suzuki cross‐coupling reactions using 10 mol% Pd(PPh_3_)_4_. Depending on the dienophile, some of the cycloaddition reactions required heating to 60 °C, the use of neat BF_3_ ⋅ Et_2_O, and/or other Lewis acid catalysts like neat TiCl_4_ to afford the cycloaddition products in good yields and at reasonable timescales.^[11]^ The more forcing catalysis conditions required for cycloaddition reactions to occur with **1** made them ideal candidates for challenging the reactivity of ligand‐centered borenium ions generated in (^R^TBDPhos)PdCl_2_ complexes.

Diels–Alder (DA) reactivity with ^R^TBDPhos complexes was first established by screening the reactivity of the 2‐bromo‐1,3‐butadiene **1**, acrolein (**2 a**), and 10 mol% (^MeO^TBDPhos)PdCl_2_ ^[12]^ with different solvents and Brønsted acid activators (Table 1). Although several TBDPhos derivatives are known, (^MeO^TBDPhos)PdCl_2_ was initially selected because we had shown previously that the methoxy substituents attached to phosphorus give rise to higher reactivity at the TBD backbone when compared to other substituents (as discussed below).^[8d]^ The 10 mol% of catalyst and activator was selected for subsequent comparison to cross coupling reactions that used the same loading with some of the brominated DA products in prior work with Pd(PPh_3_)_4_.^[11]^

**Table 1:** Optimization of Diels–Alder reaction conditions.

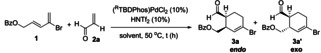						
	cat:HX	t [h]	solvent	R	Yield [%]^[b]^	*endo:exo*
1	1 : 1	12	PhMe^[d]^	OMe	–^[c]^	–
2	1 : 1	24	MeCN	OMe	–	–
3	1 : 1	1	THF	OMe	nd^[e]^	–
4	1 : 1	24	DMF	OMe	–	–
5	1 : 1	0.5	DCM	OMe	37	75 : 25
6	1 : 1	5	DCM	Ph	45	29 : 71
7	1 : 1	12	DCM	O^*i*^Pr	–	67 : 33
8	1 : 1	0.2	DCM	O^*i*^Pr‐F	24	67 : 33
9	1:0	8	DCM	OMe	nd	–
10	0 : 1	8	DCM	–	nd	–

[a] [1]=1 M. Yields reported are after column purification. Diastereomeric ratios were determined using ^1^H NMR spectroscopy. [b] nd=product **3** not detected. [c] 50 % of **1** recovered after column chromatography. [d] Reaction was carried out at 80 °C. [e] Catalyst decomposed.

Initial DA catalysis attempts in toluene with HCl and HOTf activators gave no detectable reaction even when the mixtures were heated to 80 °C for 12 h. We have shown previously that chloride and triflate can bind to the TBD backbone when protonated,^[8a]^ which suggested the need for an acid with a more non‐nucleophilic counter anion. Indeed, conducting the reactions with 1 : 1 cat/HNTf_2_ in toluene yielded formation of the DA product, but the reaction remained incomplete after 12 h of heating (Entry 1). Conducting the same reactions in MeCN, THF, DMF yielded no improvements, but switching to DCM resulted in complete consumption of the diene in 30 min. The DA product **3** was isolated in 37 % yield as an inseparable mixture of endo/exo isomers (dr 75 : 25) after column chromatography (Entry 5). Control experiments with only HNTf_2_ or (^MeO^TBDPhos)PdCl_2_ (Entries 9 and 10) showed no formation of **3** indicating that both must be present for the DA reaction to occur.

**Figure 2:**
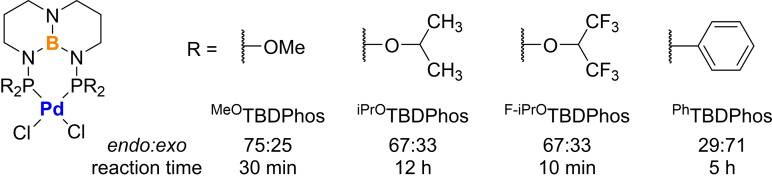
Different (^R^TBDPhos)PdCl_2_ variants tested for the reaction in Table 1.

**Figure 3:**
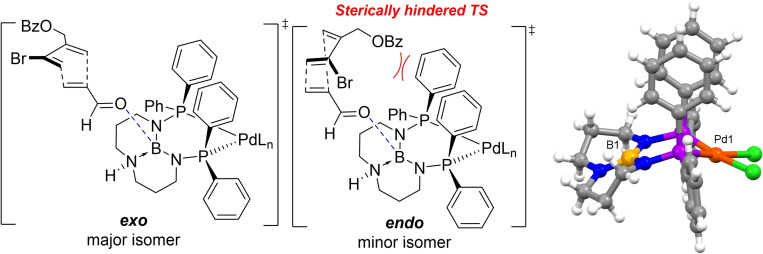
*Left* – comparison of postulated transition state differences for borenium‐catalyzed cycloaddition reactions with (PhTBDPhos)PdCl_2_. *Right* – ball and stick plot of the crystal structure of (PhTBDPhos)PdCl_2_ showing the picketed Ph groups.^[8c]^

With optimized conditions in hand, we tested several other variations of (^R^TBDPhos)PdCl_2_ with different R substituents attached to phosphorus (Figure 2).^[12]^ Prior DFT studies of ligand‐centered reactions with (^R^TBDPhos)Pt(S_2_C_6_H_4_) and MeOH showed that that the reactions were ca. 5 kcal/mol more exergonic with R=MeO compared to R=Ph.^[8d]^ Consistent with the decreased reactivity with R=Ph, the cycloaddition reaction to form **3** with (^Ph^TBDPhos)PdCl_2_ was significantly slower than (^MeO^TBDPhos)PdCl_2_ (5 h vs. 0.5 h). However, the change to (^Ph^TBDPhos)PdCl_2_ inverted the endo/exo stereoselectivity of the reaction from dr 75 : 25 with R=MeO to dr 29 : 71 with R=Ph (Figure 2). This type of stereoselectivity inversion with open chain dienes has been observed in comparative DA catalyzed reactions with BF_3_ ⋅ Et_2_O and B(C_6_F_5_)_3_ with the latter showing the greater preference for the more‐difficult‐to‐access exo products.^[13]^ The preference for exo stereoselectivity was attributed to the increased steric bulk of the C_6_F_5_ substituents that prevented endo approach of the diene in the transition state. A similar steric argument could be made for ^Ph^TBDPhos, which has rigid Ph groups that picket themselves to inhibit endo approach of the diene, as shown in the crystal structure of (^Ph^TBDPhos)PdCl_2_ (Figure 3), but a more detailed mechanistic study is needed to rule out other possibilities. We note that this catalyst dependent change in endo/exo ratio only occurs with open, acyclic dienes and does not occur in reactions with the cyclic dienes.

We next tested the influence of (^R^TBDPhos)PdCl_2_ with bulkier alkoxy groups attached to phosphorus. Switching to R=O^*i*^Pr attenuated formation of **3** and significant amounts of diene **1** remained unreacted after 12 h (Entry 7). In contrast, using R=OCH(CF_3_)_2_ (O^*i*^Pr−F) with fluorinated isopropyl groups dramatically increased reactivity and decreased the time needed to consume **1** to 10 min (Entry 8). However, more reaction by‐products were observed by TLC and NMR spectroscopy that corresponded to lower isolated yield of **3** (24 %). The increased steric bulk of the substituents attached to oxygen led to only a slight increase in the exo isomer (*dr* 67 : 33) compared to R=MeO.

**Scheme 3:**
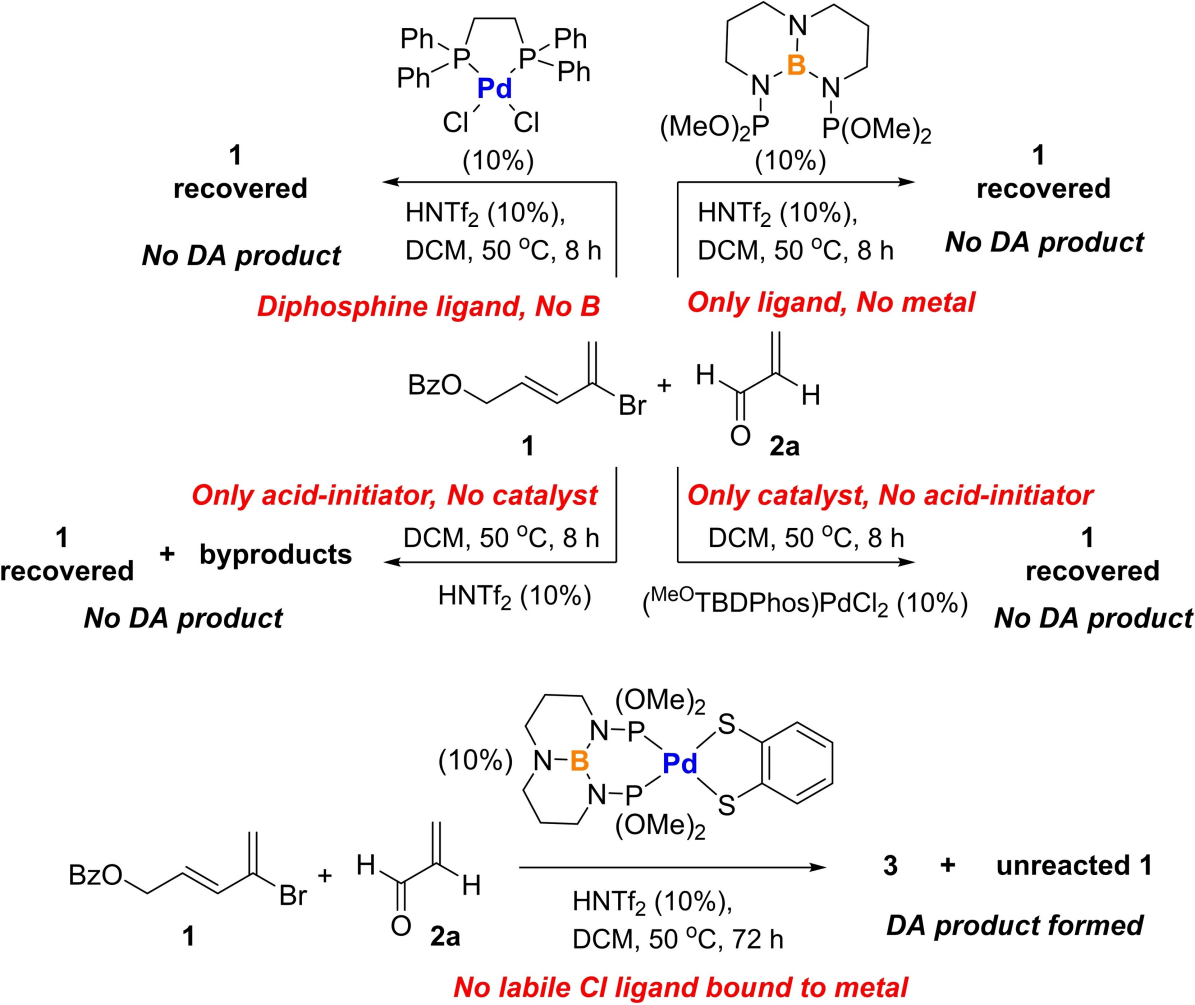
Control experiments to determine the significance of each group in the functioning DA catalyst.

Control reactions were carried out to determine the significance of each moiety in the catalyst (Scheme 3). Separate reactions with only the ^MeO^TBDPhos ligand and HNTf_2_ (no Pd) and (dppe)PdCl_2_ and HNTf_2_ (no boron ligand; dppe=1,2‐bis(diphenylphosphino)ethane) showed no formation of **3**. The lack of reactivity with only ^MeO^TBDPhos is in alignment with our previous studies showing how TBDPhos ligands tend to decompose during attempted reactions at TBD unless the ligand is bound to a metal.^[8c]^ The results with (dppe)PdCl_2_ suggest that the DA reaction does not occur at the metal and requires boron present in the TBDPhos ligands.

To further corroborate that the DA reactions occur at boron and not Pd, we performed an additional control reaction with the 1,2‐benzenedithiolate complex (^MeO^TBDPhos)Pd(S_2_C_6_H_4_).^[12]^ We have shown in previous studies how the chloride ligands in related (^Ph^TBDPhos)NiCl_2_ are labile in the presence of acids like HNTf_2_,^[8a]^ so we had to consider the possibility that chloride displacement from the metal could provide open coordination sites where potential DA reactions could occur. Even though this chloride displacement is also likely to occur in the control reaction with (dppe)PdCl_2_ and HNTf_2_ (which showed no DA reactivity), we tested the 16‐electron (^MeO^TBDPhos)Pd(S_2_C_6_H_4_) with chelating and more strongly bound dithiolate to further rule out the possibility of metal involvement. Indeed, the DA reaction with **1** and **2 a** occurs with (^MeO^TBDPhos)Pd(S_2_C_6_H_4_) to form **3** when performed under the same loading and conditions. We note that the reaction is significantly slower than with (^MeO^TBDPhos)PdCl_2_ (some unreacted **1** remains after 3 days), but this is consistent with prior studies showing how boron reactivity at the TBD backbone is attenuated in complexes with ancillary thiolate and dithiolate ligands,[^8a^, ^8d^] as compared to identical reactions where the ancillary ligand is chloride.

Because of its combination of relatively fast reaction times and limited side product formation in our optimization studies, we proceeded with testing (^MeO^TBDPhos)PdCl_2_ with different dienophiles to evaluate the scope of different DA reactions. Reactions of 2‐bromo‐3,5‐butadiene dienophiles **2 b**–**2 d** (EWG=COOMe, CN and COCH_3_) gave products **3 b**–**3 d** in 40–70 % yields (Table 2). The reaction was also tested by varying the substituent (R=Me) in crotonaldehyde **2 e** to give product **3 e** in 20 min, 66 % (*dr* 79 : 21). The dienophiles maleic anhydride (**2 f**) and benzoquinone (**2 g**) gave products **3 f** and **3 g**, respectively, as single isomers in isolated yields of 54 and 71 %.

**Table 2:**
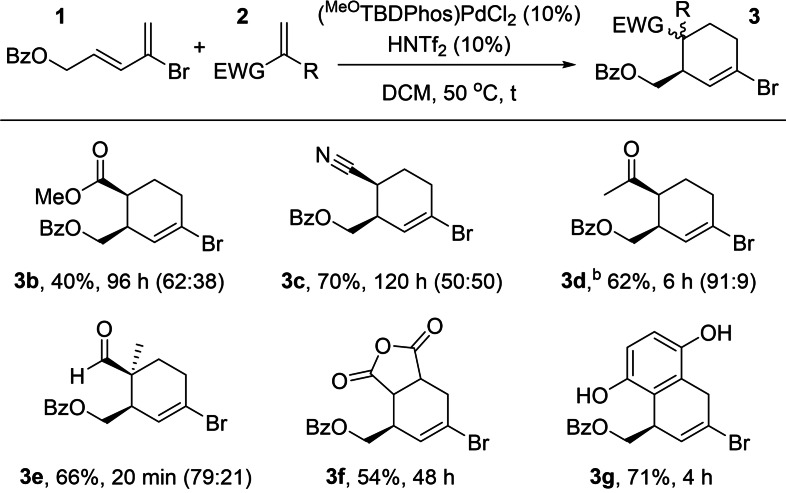
Diels–Alder reaction with brominated diene **1**.^[a]^

[a] [**1**]=1 M. Yields reported are after column purification. Diastereomeric ratios were calculated using ^1^H NMR spectroscopy. [b] 11.0 equivalent of dienophile.

To demonstrate that the DA reactions could also be used to synthesize bicyclic rings, we tested the reactivity of (^MeO^TBDPhos)PdCl_2_ with cyclopentadiene and the dienophiles shown in Table 3. Compared to reactions shown in Table 1 and Table 2, the DA reactions in Table 3 proceeded more rapidly in toluene, which is more desirable for the subsequent cross‐coupling reactions compared to chlorinated solvents like DCM (see below).^[14]^ The reaction of 4‐bromocinnamaldehyde with cyclopentadiene was carried out at −78 °C to obtain product **6 a** in 72 % yield in 5 min. For R=CH_3_ as in *trans*‐benzylideneacetone, the DA cycloaddition gave product **6 b** in excellent yield in 10 min at −20 °C (94 %; *dr* 97 : 3). The low temperatures used to prepare **6 a** and **6 b** are similar to those described for other boron‐catalyzed DA reactions with cyclopentadiene and related dienophiles.^[15]^ We also tested acyclic dienes like isoprene and butadiene to obtain the cycloadducts **6 c** and **6 d** in good isolated yields of 88 % (*dr* 91 : 9) in 10 h and 82 % (*dr*>99 : 1) in 12 h, respectively. These reactions are faster compared to those using BF_3_ ⋅ Et_2_O as the catalyst; DA reactions with isoprene and butadiene and the parent cinnamaldehyde take 24 h to complete at room temperature using 15 mol% BF_3_ ⋅ Et_2_O.^[16]^ Given these comparative metrics, we used the reaction of isoprene and 4‐bromocinnamaldehyde to further evaluate the influence of (^MeO^TBDPhos)PdCl_2_ and HNTf_2_ loading on the rate of reaction to form **6 c**. Lowering the loadings to 6 mol% increased the completion time to 24 h (similar to BF_3_ ⋅ Et_2_O at 15 mol%). Decreasing the loading further to 3 mol% slowed the reaction to the point that unreacted starting material was still observed after 3 days. These studies showed that the DA reactivity decreases as expected as the catalyst loading is reduced.

**Table 3:**
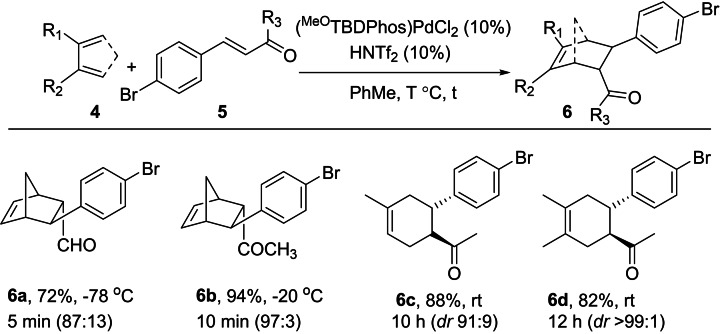
Diels–Alder Cycloaddition with brominated dienophile **5**.^[a]^

[a] [**1**]=1 M. Yields reported are after column purification. Diastereomeric ratios were calculated using ^1^H NMR spectroscopy.

Upon optimization of DA reactions with two different diene‐dienophile sets, we shifted our focus to cross‐coupling catalysis. We started with Stille reactions because they could be performed under the same acidic conditions as the DA step with the HNTf_2_ activator. The DA reaction with **1** and acrolein **2 a** was performed in DCM to form the cycloadduct **3**, as described above. After confirming the complete consumption of **1 a** by TLC, DCM was removed under vacuum, and toluene and the organotin reagent were added to the pot. The mixture was heated to 80 °C to yield the final products **7 a** (R=Ph) and **7 b** (R=vinyl) in an overall yield of 58 % (*dr* 75 : 25) and 42 % (*dr* 91 : 9), respectively (Table 4, entries 1 and 2). Changing the dienophiles, we obtained Stille coupled cycloadducts **7 c** (acrylonitrile, R=Ph) in 60 % yield (*dr*=50 : 50) and **7 d** (methacrolein, R=vinyl) in 45 % yield (*dr*=79 : 21) (Table 4, entries 3 and 4). In the case of benzoquinone and R=Ph, we obtained **7 e** as a single isomer, as observed in cycloadduct **3 g** (Table 4, entry 5). Similar reactions were demonstrated with cyclopentadiene and cyclohexadiene with dienophile **5**, followed by addition of ^*n*^Bu_3_Sn(vinyl) to obtain products **8 a** (64 %, dr 97 : 3) and **9 a** (45 %, dr 87 : 13) (Table 4, entries 6 and 7). These latter reactions were performed using toluene for both steps. We emphasize that the catalysis shown in Table 4 proceeded without any extra loading of catalyst between the first and second step.

**Table 4:**
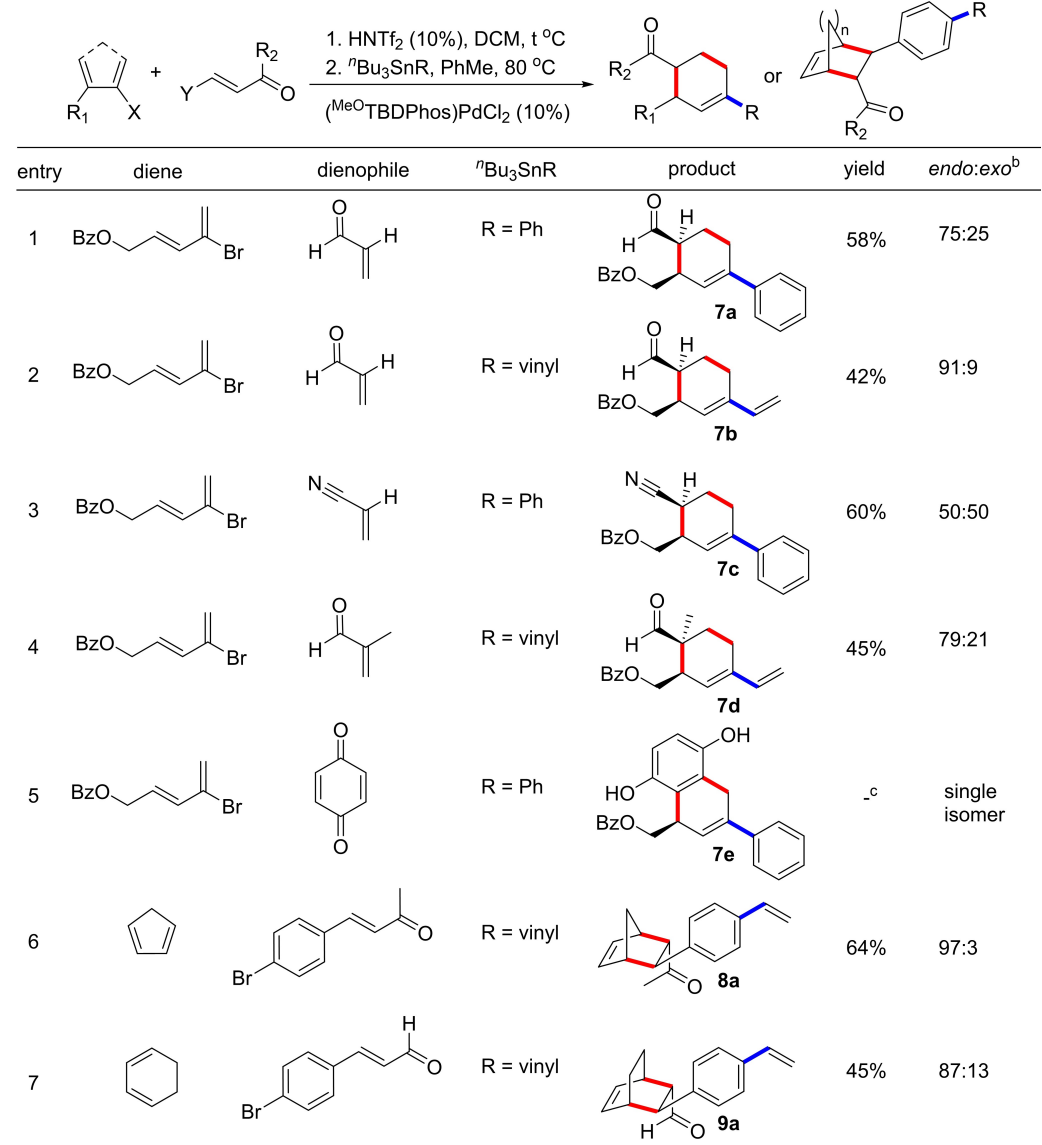
One‐pot Diels–Alder and Stille cross‐coupling results.

[a] [**1**]=1 M. Yields reported are after column purification. [b] Diastereomeric ratios were calculated using ^1^H NMR spectroscopy. [c] Yield could not be determined due to an inseparable impurity (see Supporting Information).

**Scheme 4:**
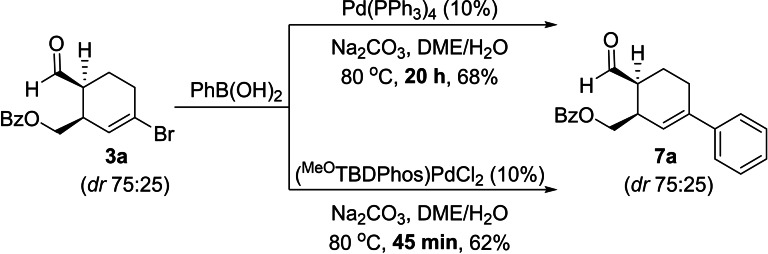
Comparison of Suzuki cross‐coupling of **3 a** and PhB(OH)_2_ using Pd(PPh_3_)_4_ and (^MeO^TBDPhos)PdCl_2_.

We next turned our attention to Suzuki cross‐coupling reactions. These reactions require stochiometric amounts of base to proceed, and it was not clear how switching from acidic conditions required for cycloaddition reactions to basic conditions required for the Suzuki reactions would affect catalyst stability and reactivity. As a baseline, we first tested the reactivity of (^MeO^TBDPhos)PdCl_2_ for Suzuki reactions only under basic conditions in the reaction of **3 a** and phenylboronic acid to form **7 a**, as described by Brimble and Furkert with Pd(PPh_3_)_4_.^[11]^ To our satisfaction, the reaction with (^MeO^TBDPhos)PdCl_2_ not only formed **7 a** with similar isolated yield, it was significantly faster than Pd(PPh_3_)_4_ (45 min vs. 20 h) when conducted under identical conditions (Scheme 4). Unfortunately, attempts to combine the reactions in a single pot failed, primarily because the transition between acidic and basic conditions decomposed the catalyst.^[17]^ Similar results were observed in one‐pot DA/Suzuki reactions aimed at preparing **8 b** (Table 5).

**Table 5:**
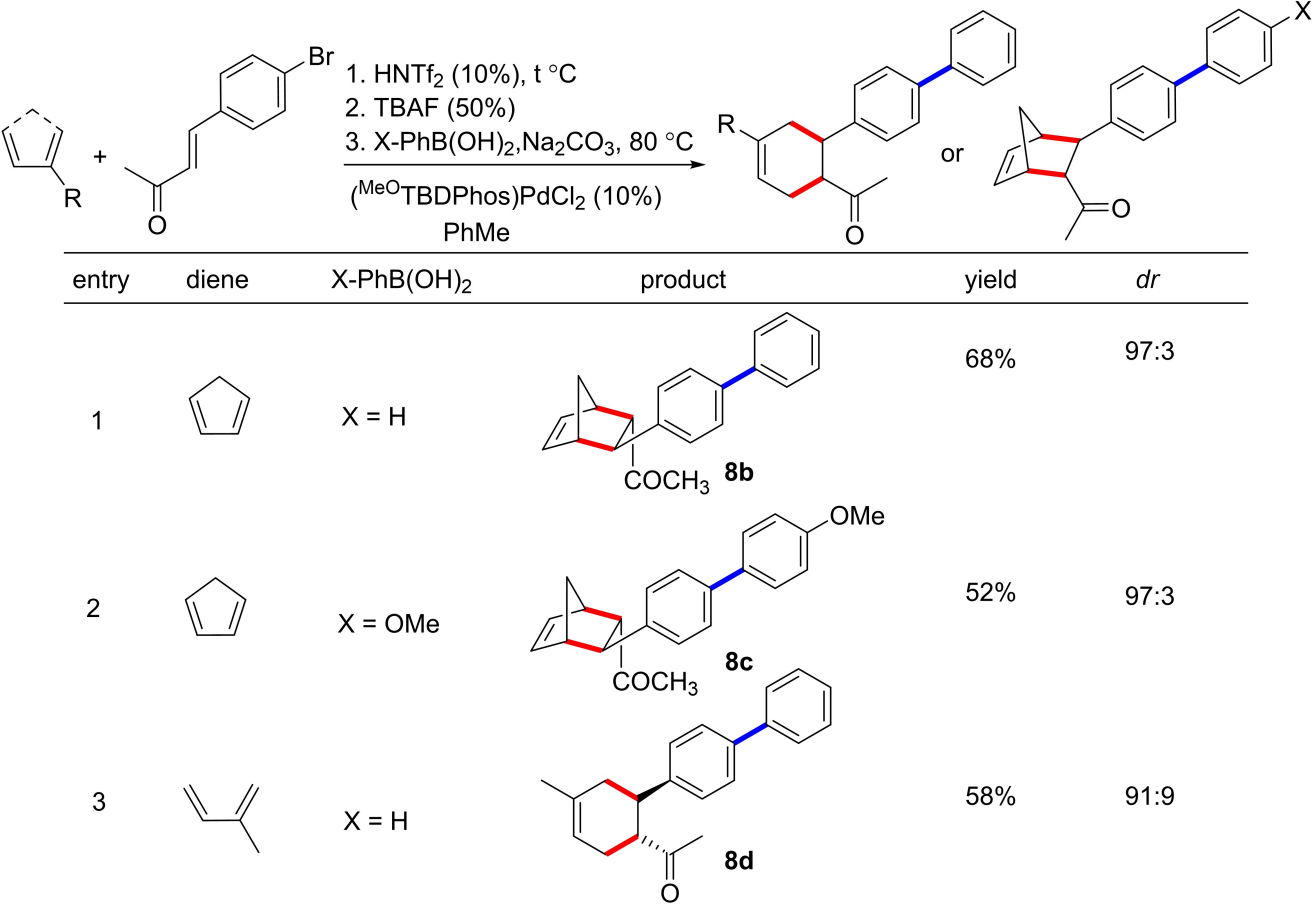
One‐pot Diels–Alder and Suzuki cross‐coupling results.

[a] [**1**]=1 M. Yields reported are after column purification. Diastereomeric ratios were calculated using ^1^H NMR spectroscopy.

**Figure 4:**
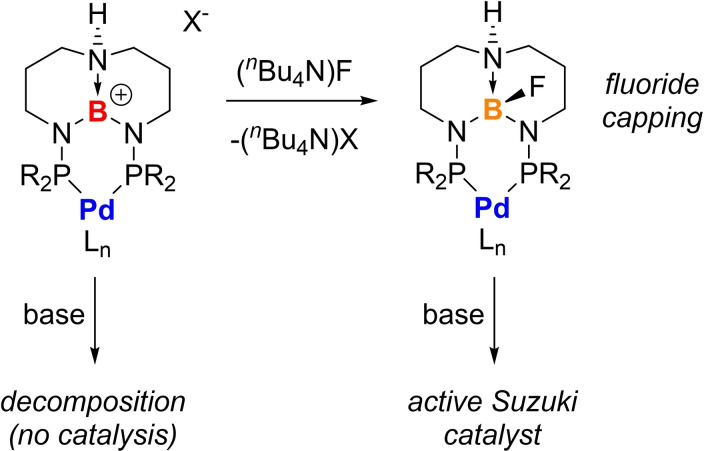
Fluoride‐capping strategy for switching between acidic cycloaddition conditions and basic Suzuki cross‐coupling conditions.

We have observed in other studies that TBDPhos complexes are significantly more stable with respect to pH‐induced decomposition when fluoride is added to boron in the TBD backbone.[^8c^, ^18^] Thus we postulated that addition of fluoride after the Diels–Alder reaction but before switching to the basic conditions required for the Suzuki reactions would attenuate ligand decomposition (Figure 4). Addition of (^*n*^Bu_4_N)F (0.5 equiv) after the DA reaction allowed the subsequent Suzuki reaction to proceed smoothly with PhB(OH)_2_ to yield **8 b** (68 %, *dr* 97 : 3; Table 5). Similar results were obtained with 4‐methoxy phenyl boronic acid to give **8 c** (52 %, *dr* 97 : 3), and we verified the one‐pot DA/Suzuki reaction works with acyclic dienes like isoprene to give **8 d** (58 %, *dr* 91 : 9) (Table 5, entry 3). These results suggest that fluoride can be used to stabilize TBDPhos catalysts for switching between acidic conditions required for cycloaddition and basic conditions required for many types of cross‐coupling reactions. Unfortunately, this fluoride capping approach was still not sufficient to achieve a one‐pot synthesis of **7 a** with PhB(OH)_2_ after the DA reaction to form **3 a**. Nevertheless, we have demonstrated that **7 a** can be prepared in one pot reactions using Stille coupling, as shown in Table 4.

## Conclusion

**Scheme 5:**
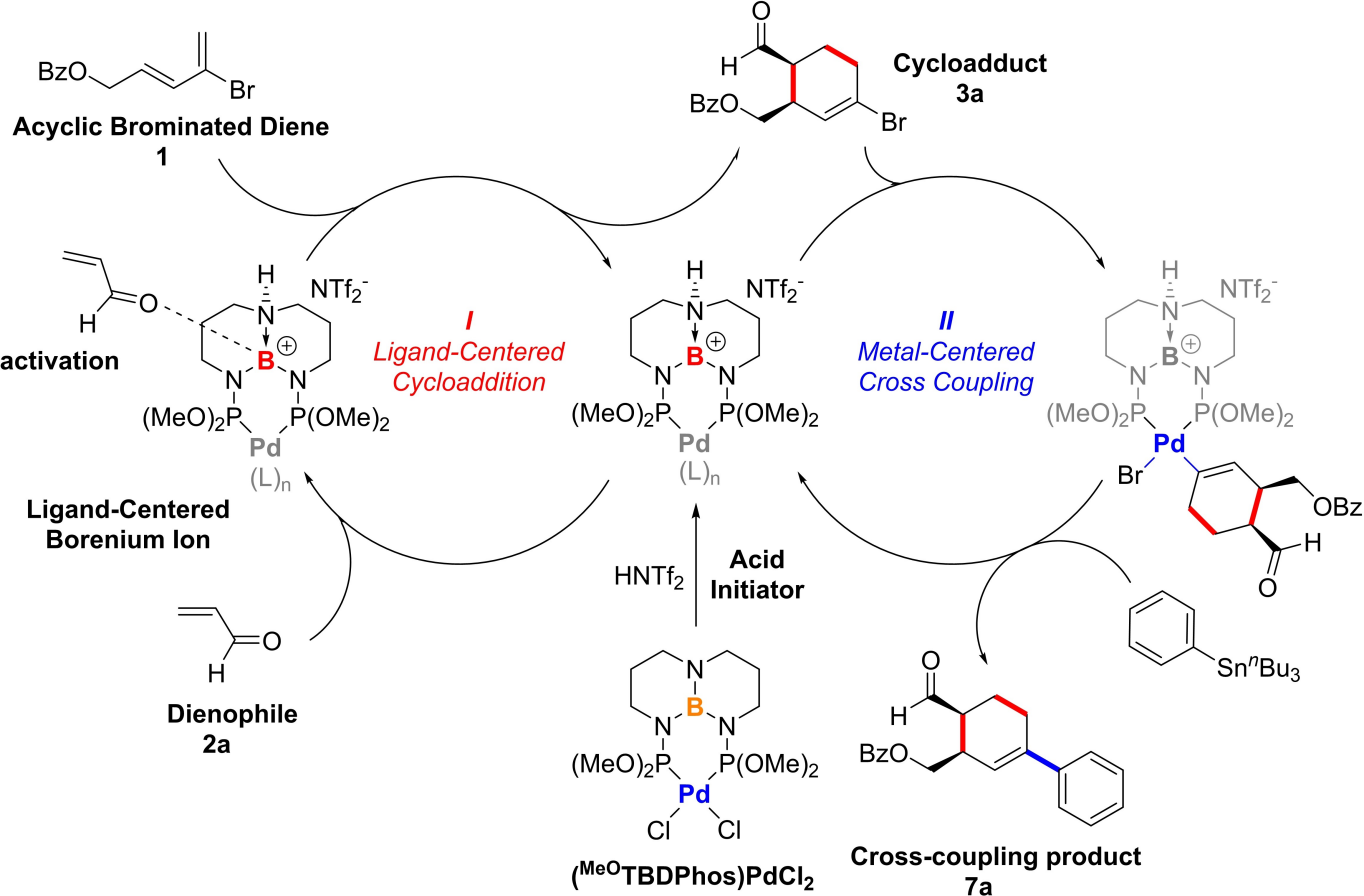
Possible catalytic cycles involved in assisted tandem catalysis with (^MeO^TBDPhos)PdCl_2_, as represented with the synthesis of **7 a** via Stille coupling.

In summary, we have demonstrated how (^MeO^TBDPhos)PdCl_2_ can serve as a multifunctional catalyst for tandem Diels–Alder (DA) and C−C cross coupling in one‐pot reactions. The results suggest that ligand‐centered borenium ions generated on the TBD backbone catalyze the formation of brominated cycloaddition products that can be functionalized using metal‐catalyzed Stille and Suzuki cross coupling reactions with the same complex (Scheme 5). The mechanistically discrete DA and cross‐coupling reactions described here are intervention‐controlled using pH, temperature, and/or solvent. Thus, these coupled catalytic reactions are defined as assisted tandem catalysis.^[3a]^ An advantage of these and other examples of assisted tandem catalysis is that both catalytic processes can be optimized separately to maximize tandem yield and stereoselectivity of the final product.

A distinguishing feature of these reactions that sets them apart from other examples of tandem catalysis is that they appear to use separate metal and ligand reactive sites in a single complex. In this context, it is notable that there are many examples of well‐defined inner‐ and outer‐sphere boron ligands that have proven capable of assisting metals in cooperative reactions with small molecules,[^4j^, ^19^] but we are not aware of any that have been used to carry out transformations in tandem with separate catalytic reactions at the metal.

We note that the primary focus of this initial work was to demonstrate how tandem reactivity can be achieved using reactive TBDPhos ligands. There is still much to learn about the mechanistic details of these reactions and the interdependent reactivity of metal and boron (especially with different phosphorus substituents and ancillary ligand combinations). However, given the immense catalogue of known Lewis‐acid‐catalyzed reactions (such as cycloaddition) and late‐transition‐metal‐catalyzed reactions (such as cross coupling), we view the demonstrated one‐pot reactions described here as important first steps into a new and highly fertile area of multifunctional catalysis research using reactive ligands.

## Experimental Section

**General Considerations**: Unless otherwise noted, all reactions for the preparation of the substrates were performed in oven‐dried glassware under nitrogen with solvents that were freshly distilled or dried using a Glass Contour Solvent Purification System (Pure Process Technology) and stored over molecular sieves. (^R^TBDPhos)PdCl_2_ complexes were synthesized as described previously[^8c^, ^12^] and stored in a nitrogen‐filled glovebox prior to use. All commercial reagents were used without further purification unless otherwise indicated.

Catalytic reactions were magnetically stirred and monitored by thin layer chromatography carried out on 0.25 mm E. Merck silica gel plate (60f–254) using UV light as visualizing agent followed by KMnO_4_ as a TLC stain. Separation of mixtures was performed by flash chromatography using silica gel (60 Å pore size) with the denoted solvent system.

^1^H and ^13^C NMR data were recorded on a Bruker AVANCE (300‐MHz) or a Bruker AVANCE (500‐MHz) NMR spectrometers using chloroform‐*d* (CDCl_3_) as the internal standards. All NMR chemical shifts are recorded in parts per million relative to the chloroform reference peaks at δ 7.26 and δ 77.0 for ^1^H and ^13^C, respectively. Coupling constants are reported in hertz. ^1^H NMR spectra are reported as chemical shift in ppm, followed by relative integral, multiplicity (“s” singlet, “bs” broad singlet, “d” doublet, “dd” doublet of doublets, “dt” doublet of triplet, “t” triplet, “q” quartet, “p” pentet, “m” multiplet), coupling constant where applicable, and assignment.

## Synthesis

**Standard catalytic procedure for the synthesis of Diels–Alder products 3 a**–**3 g**: (^MeO^TBDPhos)PdCl_2_ (57.5 mg, 0.11 mmol, 0.1 equiv.) was weighed out in an oven‐dried catalytic tube in the glovebox and dissolved in dry and degassed DCM (1.0 ml). The DCM from the diene **1** (0.30 g, 1.15 mmol, 1.0 equiv.), which was stored at 4 °C as a 0.2 M solution, was removed under vacuum. It was then dissolved in degassed DCM (2.0 ml) and added to the solution of **1** under nitrogen atmosphere. The catalytic tube was sealed using a rubber septum and acrolein **2 a** (193.2 mg, 3.44 mmol, 3.0 equiv.) was added. To this solution, HNTf_2_ (32.2 mg, 0.11 mmol, 0.1 equiv.) dissolved in degassed DCM (2.0 mL) was added dropwise. The reaction mixture turned red and was added to an oil bath preheated to 50 °C for 30 min. Upon completion (as monitored by TLC), the reaction was cooled to room temperature, quenched with saturated NaHCO_3_ solution (10 ml), extracted using DCM (2×10 ml), and concentrated to obtain crude product **3**. Column chromatography on silica column (ethyl acetate/hexane=1/10) afforded an inseparable mixture of **3 a** and **3 a’** (450 mg, 37 %, *endo*/*exo* 3 : 1) as a pale‐yellow wax.

Diels–Alder cycloadducts **3 b**–**3 g** were prepared as described for **3 a**.

**Standard catalytic procedure for the synthesis of Diels–Alder products 6 a**–**6 d**: (^MeO^TBDPhos)PdCl_2_ (57.5 mg, 0.11 mmol, 0.1 equiv.) was weighed out in oven‐dried catalytic tube in the glovebox. 4‐Bromocinnamaldehyde **5 a** (0.30 g, 1.42 mmol, 1.0 equiv.) was dissolved in dry and degassed toluene (2.0 ml) and added to the tube under nitrogen atmosphere. The catalytic tube was sealed using a rubber septum and cyclopentadiene **4 a** (0.28 g, 4.25 mmol, 3.0 equiv.) was added. The reaction mixture was allowed to cool to −78 °C and HNTf_2_ (39.8 mg, 0.14 mmol, 0.1 equiv.) dissolved in degassed toluene (2.0 mL) was added dropwise. The reaction mixture turned dark purple and within 5 min was quenched with saturated NaHCO_3_ solution (10 ml), extracted using EtOAc (2×10 ml), and concentrated to obtain crude product **3**. Column chromatography on silica column neutralized with Et_3_N (EtOAc/hexane=1/20) afforded an inseparable mixture of **6 a** and **6 a’** (0.28 g, 72 %, *endo*/*exo* 87 : 13) as a colorless wax.

Diels–Alder cycloadducts **6 b** ‐ **6 d** were prepared as described for **6 a** with the temperature of HNTf_2_ addition being −20 °C for **6 b** and 0 °C for **6 c** and **6 d**.

**Standard catalytic procedure for one‐pot Diels–Alder and Stille coupling to obtain 7 a–7 e, 8 a, and 9 a**: (^MeO^TBDPhos)PdCl_2_ (22.0 mg, 0.044 mmol, 0.1 equiv.) was weighed out in an oven‐dried catalytic tube in the glovebox. *trans*‐Benzylideneacetone **5 b** (100.0 mg, 0.44 mmol, 1.0 equiv.) was dissolved in dry and degassed toluene (1.0 ml) and added to the tube under nitrogen atmosphere. The catalytic tube was sealed using a rubber septum and cyclopentadiene **4 a** (88.0 mg, 1.33 mmol, 3.0 equiv.) was added. The reaction mixture was cooled to −20 °C and HNTf_2_ (12.0 mg, 0.044 mmol, 0.1 equiv.) dissolved in degassed toluene (2.0 mL) was added dropwise. The reaction mixture turned dark purple and was monitored using TLC. Once complete consumption of the DA starting material was confirmed, ^*n*^Bu_3_Sn(vinyl) (169 mg, 0.53, 1.2 equiv.) was added. The reaction was heated to 80 °C for 4 h, quenched using saturated aqueous KF solution (10 ml), and extracted using EtOAc (2×10 ml). The organic layer was further washed with KF solution (2×5 ml), water (10 ml), dried over MgSO_4_, and concentrated under vacuum to obtain the crude product. Column chromatography on silica column neutralized with Et_3_N (EtOAc/hexane=1/20) afforded **8 a** (68 mg, 64 %, *endo*/*exo* 97 : 3) as a colorless oil.

Stille products **7 a**–**7 e** and **9 a** were prepared using the same procedure as described above.

**Standard catalytic procedure for one‐pot Diels–Alder and Suzuki coupling to obtain 8 b**–**8 d**: (^MeO^TBDPhos)PdCl_2_ (22.0 mg, 0.044 mmol, 0.1 equiv.) was weighed out in an oven‐dried catalytic tube in the glove box. *trans*‐Benzylideneacetone **5 b** (100.0 mg, 0.44 mmol, 1.0 equiv.) was dissolved in dry and degassed toluene (1.0 ml) and added to the tube under nitrogen atmosphere. The catalytic tube was sealed using a rubber septum and cyclopentadiene **4 a** (88.0 mg, 1.33 mmol, 3.0 equiv.) was added. The reaction mixture was allowed to cool to −20 °C and HNTf_2_ (12.0 mg, 0.044 mmol, 0.1 equiv.) dissolved in degassed toluene (2.0 mL) was added dropwise. The reaction mixture turned dark purple and was monitored using TLC. Once complete consumption of the DA starting material was confirmed, TBAF (58.1 mg, 0.22 mmol, 0.5 equiv.) dissolved in dry toluene (1 ml) was added and the mixture was stirred at room temperature for 15 min. PhB(OH)_2_ (65.0 mg, 0.53 mmol, 1.2 equiv.) and Na_2_CO_3_ (56.0 mg, 0.53 mmol, 1.2 equiv.) dissolved in degassed water (1.0 ml) was then added. The reaction was heated to 80 °C for 1 h, quenched with water (10 ml), and extracted using EtOAc (2×10 ml). The organic layer was washed with brine (10 ml), dried over MgSO_4_, and concentrated under vacuum to obtain the crude product. Column chromatography on silica column neutralized with Et_3_N (EtOAc/hexane=1/20) afforded **8 b** (87.0 mg, 68 %, *endo*/*exo* 97 : 3) as a white solid.

Suzuki products **8 c** and **8 d** was prepared using the same procedure as described above.

## Characterization

**(3‐bromo‐6‐formylcyclohex‐2‐en‐1‐yl)methyl benzoate (3 a)**: Colorless wax, (45.0 mg, 0.14 mmol, 37 %); ^1^H NMR (500 MHz, CDCl_3_): major isomer δ 9.86 (s, 1H), 8.01–7.96 (m, 2H), 7.59–7.55 (m, 1H), 7.47–7.43 (m, 2H), 6.14–6.13 (m, 1H), 4.45 (dd, *J*=11.5, 5.4 Hz, 1H), 4.28–4.23 (m, 1H), 3.23–3.18 (m, 1H), 2.78–2.74 (m, 1H), 2.61–2.50 (m, 2H), 2.10–2.02 (m, 2H); ^13^C NMR (125 MHz, CDCl_3_): δ 201.6, 166.0, 133.3, 129.6, 129.5, 128.5, 126.7, 125.2, 63.5, 47.5, 38.0, 33.7, 20.7, two peaks are merged. ^1^H NMR (500 MHz, CDCl_3_): minor isomer **3a**’ δ 9.72 (d, *J*=1.4 Hz), 6.08 (p, 1H), 4.34 (dd, *J*=11.0, 5.7 Hz, 1H), 3.14–3.11 (m, 1H), 1.97–1.91 (m, 1H); ^13^C NMR (125 MHz, CDCl_3_): 202.0, 166.2, 133.2, 129.4, 128.4, 126.7, 65.8, 47.1, 37.0, 33.2, 22.5, other peaks are merged.

**3‐bromo‐6‐(methoxycarbonyl)cyclohex‐2‐en‐1‐yl)methyl benzoate (3 b)**: Colorless oil, (53 mg, 0.15 mmol, 40 %); ^1^H NMR (500 MHz, CDCl_3_): δ 8.05–8.00 (m, 2H), 7.59–7.55 (m, 1H), 7.47–7.43 (m, 2H), 6.13–6.11 (m, 1H), 4.31 (d, *J*=8.2 Hz, 1H), 4.26 (dd, *J*=8.2, 3.5 Hz, 1H), 3.57 (s, 3H), 3.15–3.09 (m, 1H), 2.86–2.81 (m, 1H), 2.58–2.50 (m, 2H), 2.05–1.96 (m, 2H); ^13^C NMR (125 MHz, CDCl_3_): δ 179.4, 166.3, 133.2, 130.0, 129.7, 128.5, 127.3, 124.5, 64.5, 51.9, 40.0, 38.6, 34.2, 23.0, two peaks are merged. ^1^H NMR (500 MHz, CDCl_3_): minor isomer 3b’ δ 6.04–6.02 (m, 1H), 4.56–4.52 (m, 1H), 4.38–4.34 (m, 1H) 3.63 (s, 3H); ^13^C NMR (125 MHz, CDCl_3_): most of the peaks are merged.

**((1S,6S)‐3‐bromo‐6‐cyanocyclohex‐2‐en‐1‐yl)methyl benzoate (3 c)**: White solid, (86 mg as *endo/exo* mixture, 0.27 mmol, 70 %); ^1^H NMR (500 MHz, CDCl_3_): δ 8.07–8.03 (m, 2H), 7.62–7.56 (m, 1H), 7.49–7.43 (m, 2H), 5.98 (q, *J*=3.5 Hz, 1H), 4.56–4.51 (m, 1H) 4.41–4.34 (m, 1H), 3.25–3.20 (m, 1H), 3.01–2.94 (m, 1H), 2.88–2.75 (m, 1H), 2.61–2.51 (m, 1H), 2.27–2.17 (m, 1H), 2.06–1.95 (m, 1 H); ^13^C NMR (125 MHz, CDCl_3_): δ 166.0, 133.4, 129.7, 129.4, 128.5, 125.0, 124.0, 118.6, 64.8, 38.9, 32.0, 26.9, 25.9, two peaks are merged.

**((1S,6R)‐3‐bromo‐6‐cyanocyclohex‐2‐en‐1‐yl)methyl benzoate (3 c’)**: White solid, (86 mg as *endo/exo* mixture, 0.27 mmol, 70 %); ^1^H NMR (500 MHz, CDCl_3_): δ 8.05–8.03 (m, 2H), 7.61–7.58 (m, 1H), 7.49–7.45 (m, 2H), 6.03 (q, *J*=1.7 Hz, 1H), 4.41–4.34 (m, 2H), 3.00–2.95 (m, 1H), 2.85–2.81 (m, 1H), 2.70–2.63 (m, 1H), 2.61–2.53 ( m, 1H), 2.25–2.17 (m, 1H), 2.11–2.05 (m, 1H); ^13^C NMR (125 MHz, CDCl_3_): δ 166.1, 133.4, 129.7, 129.3, 128.5, 125.6, 124.0, 120.6, 64.8, 40.8, 33.0, 26.6, 25.6, two carbon peaks are merged.

**((1S,6S)‐6‐acetyl‐3‐bromocyclohex‐2‐en‐1‐yl)methyl benzoate (3 d)**: White solid, (80 mg, 0.24 mmol, 62 %); ^1^H NMR (500 MHz, CDCl_3_): δ 7.98–7.96 (m, 2H), 7.58–7.55 (m, 1H), 7.46–7.43 (m, 2H), 6.14–6.12 (m, 1H), 4.34–4.31 (m, 1H), 4.20–4.16 (m, 1H), 3.18–3.13 (m, 1H), 2.87–2.83 (m, 1H), 2.58–2.48 ( m, 2 H), 2.19 (s, 3H), 2.08–1.98 (m, 1 H), 1.96–1.90 (m, 1H); ^13^C NMR (125 MHz, CDCl_3_): δ 208.7, 166.1, 133.2, 129.7, 129.6, 128.5, 127.6, 127.1, 63.6, 47.8, 38.9, 34.4, 29.0, 21.7, two carbon peaks are merged. ^1^H NMR (500 MHz, CDCl_3_): minor isomer 3d’ δ 6.06–6.04 (m, 1H), 4.27–4.23 (m, 1H), 4.16–4.13 (m, 1H), 2.20 (s, 3H), ^13^C NMR (125 MHz, CDCl_3_): δ 209.5, 127.7, 124.8, 122.8, 66.3, 38.9, 34.2, 26.5, other peaks are merged.

**3‐bromo‐6‐formyl‐6‐methylcyclohex‐2‐en‐1‐yl)methyl benzoate (3 e)**: Colorless solid, (85 mg, 0.25 mmol, 66 %); ^1^H NMR (500 MHz, CDCl_3_): δ 9.70 (s, 1H), 8.00–7.98 (m, 2H), 7.60–7.56 (m, 1H), 7.48–7.44 (m, 2H), 6.12–6.10 (m, 1H), 4.44–4.38 (m, 1H), 4.34–4.30 (m, 1H), 2.73–2.70 (m, 1H), 2.63–2.56 (m, 1H), 2.55–2.51 ( m, 1H), 2.07 (p, *J*=6.8 Hz, 1 H), 1.75–1.70 (m, 1H), 1.23 (s, 3H); ^13^C NMR (125 MHz, CDCl_3_): δ 203.5, 166.1, 133.3, 129.6, 128.5, 126.4, 123.8, 63.7, 45.9, 44.9, 40.7, 32.1, 28.8, 19.7, two carbon peaks are merged. ^1^H NMR (500 MHz, CDCl_3_): minor isomer 3e’ δ 9.54 (s, 1H), 7.60–7.56 (m, 1H), 6.01–6.00 (m, 1H), 4.31–4.27 (m, 1H), 4.21–4.17 (m, 1H), 3.15–3.11 (m, 1H), 1.95–1.89 (m, 1H), 1.64–1.59(m, 1H); 13 C NMR (100 MHz; CDCl3): δ 204.2, 129.6, 129.5, 128.5, 126.2, 64.1, 45.8, other peaks are merged.

**((4S)‐6‐bromo‐1,3‐dioxo‐1,3,3 a,4,7,7 a‐hexahydroisobenzofuran‐4‐yl)methyl benzoate (3 f)**: White solid, (75 mg, 0.15 mmol, 54 %); ^1^H NMR (500 MHz, CDCl_3_): δ 8.03–8.01 (m, 2H), 7.61–7.58 (m, 1H), 7.48–7.45 (m, 2H), 6.27 (dd, *J*=3.9, 2.8 Hz, 1H), 4.76 (dd, *J*=11.5, 7.0 Hz, 1H), 4.64 (dd, *J*=11.5, 7.2 Hz, 1H), 3.60–3.58 (m, 2H), 3.10 (d, *J*=2.1 Hz, 1H), 3.07 (d, *J*=1.3 Hz, 1H), 2.98–2.93 (m, 1H); ^13^C NMR (125 MHz, CDCl_3_): δ 172.2, 170.4, 166.1, 133.4, 129.7, 129.4, 129.0, 128.5, 120.4, 63.4, 41.1, 40.9, 37.4, 33.6, two carbon peaks are merged; ESI‐(MS+Na) calcd. for C_16_H_13_BrO_5_: 386.9844; Found: 386.9838.

**(R)‐(3‐bromo‐5,8‐dihydroxy‐1,4‐dihydronaphthalen‐1‐yl)methyl benzoate (3 g)**: White solid, (102 mg, 0.28 mmol, 71 %); ^1^H NMR (500 MHz, CDCl_3_): δ 7.90–7.89 (m, 2H), 7.58–7.54 (m, 1H), 7.44–7.41 (m, 2H), 6.81 (d, *J*=11 Hz, 1H), 6.77 (d, *J*=11 Hz, 1H), 6.25–6.24 (m, 1H), 4.55 (dd, *J*=11.0, 4.4 Hz, 1H), 4.43 (dd, *J*=11.0, 3.6 Hz, 1H), 3.97–3.93 (m, 1H), 3.51–3.45 (m, 1H), 3.33–3.27 (m, 1H), two OH protons; ^13^C NMR (125 MHz, CDCl_3_): δ 185.7, 166.2, 140.4, 138.0, 136.8, 136.5, 136.1, 133.3, 129.5, 129.4, 128.5, 126.2, 119.4, 65.5, 38.1, 33.4, two carbon peaks are merged.

**(1R,2S,3S,4S)‐3‐(4‐bromophenyl)bicyclo[2.2.1]hept‐5‐ene‐2‐carbaldehyde (6 a)**: Pale‐yellow wax, (94 mg, 0.34 mmol, 72 %); ^1^H NMR (500 MHz, CDCl_3_): δ 9.59 (d, *J*=2.1 Hz, 2H), 7.42 (d, *J*=8.5 Hz, 1H), 7.13 (d, *J*=8.2 Hz, 2H), 6.41 (dd, *J*=8.9, 3.2 Hz, 1H), 6.17 (dd, *J*=5.7, 2.8 Hz, 1H), 3.35 (bs, 1H), 3.09 (bs, 1H), 3.04 (d, *J*=4.6 Hz, 1H), 2.92–2.90 (m, 1H), 1.75 (d, *J*=8.8 Hz, 1 H), 1.65–1.62 (m, 1H); ^13^C NMR (125 MHz, CDCl_3_): δ 202.9, 142.6, 139.1, 133.8, 131.6, 131.2, 129.1, 120.0, 61.0, 48.2, 47.1, 45.0, two carbon peaks are merged. ^1^H NMR (500 MHz, CDCl_3_): minor isomer 6a’ δ 9.90 (d, *J*=2.0 Hz, 2H), 7.36 (d, *J*=8.5 Hz, 1H), 7.01 (d, *J*=8.2 Hz, 2H), 6.35 (dd, *J*=8.9, 3.2 Hz, 1H), 6.05 (dd, *J*=5.7, 2.8 Hz, 1H), 3.70 (bs, 1H), 3.23 (bs, 1H), 3.18 (d, *J*=4.6 Hz, 1H), 2.54–2.52 (m, 1H), 1.58–1.57 (m, 1H); ^13^C NMR (125 MHz, CDCl_3_): δ 202.2, 136.5, 129.6, 59.5, 48.3, 47.5, 45.2, other peaks are merged.

**1‐((1R,2S,3S,4S)‐3‐(4‐bromophenyl)bicyclo[2.2.1]hept‐5‐en‐2‐yl)ethan‐1‐one (6 b)**: Pale‐yellow wax, (366 mg, 1.26 mmol, 94 %); ^1^H NMR (500 MHz, CDCl_3_): δ 7.40 (d, *J*=14.2 Hz, 2H), 7.13 (d, *J*=13.9 Hz, 2H), 6.39 (dd, *J*=9.3, 5.4 Hz, 1H), 6.02 (dd, *J*=9.5, 4.6 Hz, 1H), 3.34 (bs, 1H), 3.16–3.14 (m, 1H), 2.99–2.96 (m, 2H), 2.16 (s, 3H). 1.80 (d, *J*=14.4 Hz, 1 H), 1.63–1.60 (m, 1H); ^13^C NMR (125 MHz, CDCl_3_): δ 207.6, 143.5, 139.4, 133.1, 131.5, 129.2, 119.7, 61.3, 48.2, 47.6, 46.5, 44.6, 29.0, two carbon peaks and peaks of the minor isomers are merged.

**1‐(4’‐bromo‐5‐methyl‐1,2,3,6‐tetrahydro‐[1,1’‐biphenyl]‐2‐yl)ethan‐1‐one (6 c)**: Colorless wax, (92 mg, 0.31 mmol, 88 %); ^1^H NMR (500 MHz, CDCl_3_): δ 7.40 (d, *J*=5.8 Hz, 2H), 7.07 (d, *J*=8.4 Hz, 2H), 5.46 (m, 1H), 3.05–2.99 (m, 1H), 2.95–2.90 (m, 1H), 2.25–2.10 (m, 2H), 2.20–2.12 (m, 2H), 1.88 (s, 3H), 1.69 (s, 3H); ^13^C NMR (125 MHz, CDCl_3_): δ 211.7, 143.4, 133.6, 131.7, 129.2, 120.2, 118.9, 52.8, 42.3, 38.4, 29.7, 28.8, 23.1, two carbon peaks are merged; ESI (MS+H) calcd. for C_15_H_18_BrO_5_: 293.0541; Found: 293.0538

**1‐(4’‐bromo‐4,5‐dimethyl‐1,2,3,6‐tetrahydro‐[1,1’‐biphenyl]‐2‐yl)ethan‐1‐one (6 d)**: Colorless wax, (137 mg, 0.45 mmol, 82 %); ^1^H NMR (500 MHz, CDCl_3_): δ 7.40 (d, *J*=14.1 Hz, 2H), 7.06 (d, *J*=14.1 Hz, 2H), 3.00–2.95 (m, 2H), 2.23–2.09 (m, 4H), 1.88 (s, 3H), 1.67 (s, 3H), 1.63 (s, 3H); ^13^C NMR (125 MHz, CDCl_3_): δ 211.5, 143.3, 131.6, 129.1, 125.2, 123.8, 120.2, 53.8, 42.7, 40.2, 34.9, 29.6, 18.6, 18.6, two carbon peaks are merged; ESI (MS+H) calcd. for C_16_H_20_BrO: 307.0698; Found: 307.0693

**((3R)‐4‐formyl‐3,4,5,6‐tetrahydro‐[1,1’‐biphenyl]‐3‐yl)methyl benzoate (7 a)**: Colorless wax, (72 mg, 0.22 mmol, 58 %); ^1^H NMR (500 MHz, CDCl_3_): δ 9.81 (d, *J*=1.8 Hz, 1H), 8.02–7.99 (m, 3 H), 7.58–7.55 (m,1H), 7.47–7.43 (m,1H), 7.39–7.37 (m, 2H), 7.34–7.31 (m, 2H), 7.28–7.26 (m, 1H), 6.04–6.03 (m, 1H), 4.47 (dd, *J*=5.4, 11.0, 1H), 4.37–4.25 (m, 2H), 3.27–3.22 (m, 1H), 2.56–2.53 (m, 2H), 2.18 (m, 1H), 1.98–1.91 (m, 1H); ^13^C NMR (125 MHz, CDCl_3_): δ 203.2, 166.4, 141.1, 133.1, 129.8, 129.6, 128.5, 128.4, 128.3 127.4, 125.3, 122.1, 67.0, 47.1, 35.4, 26.0, 21.7, four peaks are merged.^1^H NMR (500 MHz, CDCl_3_): minor isomer 7a’ δ 9.72 (d, *J*=1.8 Hz, 1H), 6.08–6.05 (m, 1H), 3.16–3.10 (m, 1H), 2.60–2.58 (m, 2H), 2.11–2.04 (m, 1H); ^13^C NMR (125 MHz, CDCl_3_): δ 201.9, 133.2, 129.8, 126.8, 65.8, 37.0, 22.5 other peaks are merged.

**((1R)‐6‐formyl‐3‐vinylcyclohex‐2‐en‐1‐yl)methyl benzoate (7 b)**: Colorless oil, (56 mg, 0.21 mmol, 42 %); ^1^H NMR (500 MHz, CDCl_3_): δ 9.75 (s, 1 H), 8.00 (d, J=7.6 Hz, 2 H), 7.57 (t, J=7.3 Hz, 1H), 7.44 (t, J=7.7 Hz, 2H), 7.37 (dd, J=17.6, 10.7 Hz, 1 H), 5.69 (s, 1 H), 5.16 (d, J=17.6 Hz, 1 H), 5.03 ( d, J=10.7, 1H), 4.40–4.37 (m, 1H), 4.26–4.22(m, 1H), 3.17 (bs, 1H), 2.54 (bs, 1H), 2.33–2.30 (m, 1H), 2.25–2.20(m, 1H), 2.08–2.04 ( m,1H), 1.86–1.79(m,1H); ^13^C NMR (125 MHz, CDCl_3_): δ 203.2, 166.4, 138.8, 137.9, 133.1, 129.9, 129.6, 128.4, 126.4, 112.3, 66.8, 9.0, 35.3, 22.2, 21.1, two peaks are merged. ^1^H NMR (500 MHz, CDCl_3_): minor isomer 7b’ δ 9.89 (s, 1 H), 5.78 (s, 1 H), 4.49–4.46 (m, 1H), 2.75 (bs, 1H); carbon peaks are merged.

**((1S,6S)‐3‐phenyl‐6‐cyanocyclohex‐2‐en‐1‐yl)methyl benzoate (7 c)**: White solid, (62 mg as *endo/exo* mixture, 0.19 mmol, 60 %); ^1^H NMR (500 MHz, CDCl_3_): δ 8.08–8.05 (m, 2 H), 7.58 (tt, J=12.3 Hz, J=2.1 Hz, 1 H), 7.46 (t, J=13.0 Hz, 2 H), 7.38–7.27 (m, 5H), 5.98–5.96 (m, 1 H), 4.53–4.40 (m, 2 H), 3.15–3.06 (m, 1 H), 2.92–2.85 ( m, 1 H), 2.74–2.64 (m, 1 H), 2.60–2.49 (m, 1 H), 2.36–2.27 (m, 1 H), 2.17–2.05 (m, 1 H); ^13^C NMR (125 MHz, CDCl_3_): δ 166.3, 140.5, 139.5, 133.3, 129.7, 129.6, 129.5, 128.5, 128.4, 127.8, 125.3, 120.8, 115.3, 65.8, 39.2, 27.6, 25.6, 24.7 three peaks are merged. EI+ calcd. for C_21_H_19_NO_2_: 317.1416; Found: 317.1400.

**((1S,6R)‐3‐phenyl‐6‐cyanocyclohex‐2‐en‐1‐yl)methyl benzoate (7 c’)**: White solid, (62 mg as *endo/exo* mixture, 0.19 mmol, 60 %); ^1^H NMR (500 MHz, CDCl_3_): δ 8.09–8.05 (m, 2 H), 7.59 (tt, J=12.2 Hz, J=2.2 Hz, 1 H), 7.42 (t, J=13.7 Hz, 2 H), 7.42–7.27 (m, 5H), 5.91–5.89 (m, 1 H), 4.70–4.64 (m, 1 H), 4.45–4.38 (m, 1 H), 3.32–3.27 (m, 1 H), 3.14–3.06 ( m, 1 H), 2.88–2.75 (m, 1 H), 2.62–2.53 (m, 1 H), 2.39–2.29 (m, 1 H), 2.08–1.97 (m, 1 H); ^13^C NMR (125 MHz, CDCl_3_): δ 166.2, 140.6, 139.3, 133.3, 129.7, 129.6, 128.5, 128.4, 127.8, 125.3, 119.9, 119.4, 65.8, 37.3, 27.8, 24.7 five peaks are merged.

**3‐vinyl‐6‐formyl‐6‐methylcyclohex‐2‐en‐1‐yl)methyl benzoate (7 d)**: Colorless oil, (43 mg, 0.15 mmol, 45 %); ^1^H NMR (500 MHz, CDCl_3_): δ 9.74 (s, 1H), 8.00–7.97 (m, 2H), 7.58–7.55 (m, 1H), 7.46–7.43 (m, 2H), 6.42–6.36 (m, 1H), 5.77–5.76 (m, 1 H), 5.18 (d, *J*=17.6 Hz, 1H), 5.04 ( d, *J*=10.8 Hz, 1 H), 4.44–4.41 (m, 1 H), 4.36–4.33 (m, 1 H), 2.76–2.74 ( m, 1 H), 2.36–2.31 (m, 1 H), 2.27–2.20 (m, 1 H), 2.05–2.00 (m, 1 H), 1.74–1.69 (m, 1 H), 1.21 (s, 3 H); ^13^C NMR (125 MHz, CDCl_3_): δ 204.8, 166.2, 138.6, 137.2, 133.1, 129.8, 129.6, 128.5, 126.3, 112.4, 64.4, 47.0, 43.3, 27.4, 20.9, 19.9, two carbon peaks are merged. ^1^H NMR (500 MHz, CDCl_3_): minor isomer **3e**’ δ 9.58 (s, 1H), 8.07–8.02 (m, 2H), 7.46–7.43 (m, 2H), 6.41–6.34)m, 1 H), 5.61–5.60 (m, 1 H), 4.88–4.84 (m, 1H), 4.80–4.75 (m, 1 H), 4.17–4.13 (m, 1 H), 3.20–3.17 (m, 1H), 1.85–1.79 (m, 1H), 1.63–1.59 (m, 1H), 1.1 (s, 3 H); 13 C NMR (100 MHz; CDCl3): δ 205.2, 166.3, 137.3, 64.9, 38.9, 28.5, 20.1 other peaks are merged.

**1‐((1S,2R,4R)‐3‐(4‐vinylphenyl)bicyclo[2.2.1]hept‐5‐en‐2‐yl)ethan‐1‐one (8 a)**: Colorless oil, (106 mg, 0.45 mmol, 64 %); ^1^H NMR (500 MHz, CDCl_3_): δ 7.34 (d, *J*=8.5 Hz, 2H), 7.23 (d, *J*=8.2 Hz, 1H), 6.69 (dd, *J*=17.6, 10.9 Hz, 1H), 6.40 (dd, *J*=5.7, 3.3 Hz, 1H), 6.03 (dd, *J*=5.7, 2.8 Hz, 1 H), 5.71 (dd, *J*=17.6, 1.0 Hz, 1H) 5.21 (dd, *J*=10.9, 0.9 Hz, 1H) 3.34–3.32 (m, 1 H), 3.17 (dd, *J*=5.0, 1.4 Hz, 1H), 3.05 (dd, *J*=5.0, 3.5 Hz, 1H), 3.01–2.99 (m, 1H), 2.15 (s, 3H), 1.85–1.83 (m, 1H), 1.62–1.60 ( m,1H), 1.86–1.79 (m,1H); ^13^C NMR (125 MHz, CDCl_3_): δ 207.9, 144.1, 139.3, 136.4, 135.4, 133.1, 127.6, 126.3, 113.3, 61.1, 48.6, 47.5, 46.5, 45.1, 29.1, two peaks are merged; ESI‐(MS+Na) calcd. for C_17_H_18_NaO: 261.1255; Found: 261.1250.

**1‐((1S,2R,4R)‐3‐([1,1’‐biphenyl]‐4‐yl)bicyclo[2.2.1]hept‐5‐en‐2‐yl)ethan‐1‐one (8 b)**: White solid, (128 mg, 0.44 mmol, 68 %); ^1^H NMR (500 MHz, CDCl_3_): δ 7.59–7.57 (m, 2 H), 7.54–7.52 (m, 2 H), 7.44–7.41 (m, 2H), 7.35–7.33 (m, 3H), 6.42 (dd, J=5.5, 3.2, 1H), 6.04 (dd, J=5.7, 2.7, 1H), 3.36 (bs, 1H), 3.24–3.23 (m,1 H), 3.11b–3.10(m, 1H), 3.06 (bs, 1H), 2.18 (s, 3H), 1.90 (d, J=8.6, 1H), 1.65–1.63 (m, 1H); ^13^C NMR (125 MHz, CDCl_3_): δ 208.0, 143.5, 140.9, 139.4, 138.9, 133.1, 128.7, 127.9, 127.2, 127.1, 127.0, 61.1, 48.6, 47.6, 46.5, 45.0, 29.1, four peaks are merged; ESI‐(MS+Na) calcd. for C_12_H_20_NaO: 311.1412; Found: 311.1404.

**1‐((1S,2R,4R)‐3‐(4’‐methoxy‐[1,1’‐biphenyl]‐4‐yl)bicyclo[2.2.1]hept‐5‐en‐2‐yl)ethan‐1‐one (8 c)**: White solid, (113 mg, 0.17 mmol, 52 %); ^1^H NMR (500 MHz, CDCl_3_): δ 7.52–7.79 (m, 4 H), 7.31 (d, *J*=13.6 Hz, 2 H), 6.97 (d, *J*=14.8 Hz, 2 H), 6.42 (dd, *J*=9.2, 5.4 Hz, 1 H), 6.04 (dd, *J*=9.4, 4.8 Hz, 1 H), 3.85 (s, 3H), 3.35 (bs, 1 H), 3.22 (d, *J*=4.7 Hz, 1H), 3.11–3.09 (m, 1H), 3.04 (bs, 1H), 2.17 (s, 3H), 1.89 (d, *J*=8.7 Hz, 1H), 1.64–1.62 (m, 1H); ^13^C NMR (125 MHz, CDCl_3_): δ 208.0, 159.0, 142.9, 139.4, 133.1, 127.9, 127.8, 127.7, 126.7, 114.2, 114.1, 61.1, 55.3, 48.7, 47.6, 46.5, 45.0, 29.1, four peaks are merged; ESI (MS+Na) calcd. for C_22_H_22_NaO_2_: 341.1517; Found: 341.1521.

**1‐(5‐methyl‐1,2,3,6‐tetrahydro‐[1,1’:4’,1’’‐terphenyl]‐2‐yl)ethan‐1‐one (8 d)**: White solid, (149 mg, 0.51 mmol, 58 %); ^1^H NMR (500 MHz, CDCl_3_): δ 7.58–7.56 (m, 2 H), 7.53 (d, *J*=8.3 Hz, 2 H), 7.43 (t, *J*=7.4 Hz, 2 H), 7.33 (tt, *J*=7.3, 1.4 Hz, 1 H), 7.27 (d, *J*=8.1 Hz, 2 H), 5.50–5.48 (m, 1H), 3.13–3.07 (m, 1H), 3.03–2.98 (m, 1H), 2.30–2.28 (m, 2H), 2.24–2.23 (m, 2H), 1.90 (s, 3H), 1.72 (s, 3H); ^13^C NMR (125 MHz, CDCl_3_): δ 212.2, 143.3, 139.4, 133.8, 129.2, 128.7, 127.8, 127.3, 127.1, 127.0, 118.9, 53.0, 46.6, 38.6, 29.7, 28.8, 23.1, four peaks are merged; EI (MS+.) calcd. for C_21_H_22_O: 290.1671; Found: 290.1660.

**(1S,4R)‐3‐(4‐vinylphenyl)bicyclo[2.2.2]oct‐5‐ene‐2‐carbaldehyde (9 a)**: Colorless oil, (50 mg, 0.21 mmol, 45 %); ^1^H NMR (500 MHz, CDCl_3_): δ 9.50 (d, *J*=1.3 Hz, 1H), 7.39 (d, *J*=8.3 Hz, 2 H), 7.24 (d, *J*=8.2 Hz, 2H), 6.71 (dd, *J*=17.6, 10.9 Hz, 1H), 6.52 (dt, *J*=7.4, 1.3 Hz, 1H), 6.20 (dt, *J*=7.4, 1.1 Hz, 1 H), 5.73 (dd, *J*=17.6, 0.9 Hz, 1H) 5.23 (dd, *J*=10.9, 0.9 Hz, 1H) 3.20–3.18 (m, 1 H), 3.09–3.07 (m, 1H), 2.83–2.82 (m, 1H), 2.63–2.61 (m, 1H), 1.73–1.71 (m, 1H), 1.50–1.44 ( m, 1 H), 1.11–1.04 (m,1H), 0.90–0.86 (m, 1H); ^13^C NMR (125 MHz, CDCl_3_): δ 202.7, 141.7, 137.1, 136.4, 131.6, 130.9, 128.2, 126.3, 113.6, 56.0, 43.0, 36.6, 31.3, 25.6, 18.8, two peaks are merged; GCMS (MS+.) calcd. for C_17_H_18_O: 238.1358; Found: 238.1357^1^H NMR (500 MHz, CDCl_3_): minor isomer 9a’ δ 7.45 (d, *J*=8.4 Hz, 2 H), 7.14 (d, *J*=8.3 Hz, 2H), 2.78–2.74 (m, 1H); ^13^C NMR (125 MHz, CDCl_3_): δ 202.3, 136.9, 131.5, 129.7, 42.6, 36.4, 31.3, 29.7 other peaks are merged.

## Conflict of interest

There are no conflicts to declare.

1

## Supporting information

As a service to our authors and readers, this journal provides supporting information supplied by the authors. Such materials are peer reviewed and may be re‐organized for online delivery, but are not copy‐edited or typeset. Technical support issues arising from supporting information (other than missing files) should be addressed to the authors.

Supporting Information

## Data Availability

The data that support the findings of this study are available in the supplementary material of this article.
