# Supplementary material for: Leveraging Metal and Ligand Reactive Sites for One Pot Reactions: Ligand‐Centered Borenium Ions for Tandem Catalysis with Palladium
Source: Chemistry. 2022 Sep 26;28(65):e202201791. doi: 10.1002/chem.202201791 (PMC9828003; doi:10.1002/chem.202201791)

Chemistry–A European Journal

Supporting Information

Leveraging Metal and Ligand Reactive Sites for One Pot Reactions: Ligand-Centered Borenium Ions for Tandem Catalysis with Palladium

Manisha Skaria, Johnathan D. Culpepper, and Scott R. Daly*

Leveraging Metal and Ligand Reactive Sites for One Pot Reactions: Ligand-Centered Borenium Ions for Tandem Catalysis with Palladium

Manisha Skaria, Johnathan D. Culpepper, and Scott R. Daly*

Department of Chemistry, The University of Iowa, Iowa City, Iowa 52242, United States

Corresponding e-mail: scott-daly@uiowa.edu

TABLE OF CONTENTS

1. Representative synthetic procedures ---------- S1

1.1 General considerations ---------- S1

1.2 Preparation of (E)-4-bromopenta-2,4-dien-1-yl benzoate (**1**) ---------- S1

1.3 Standard catalytic procedures ---------- S3

1.3.1 Synthesis of Diels-Alder product **3a** – **3g** ---------- S3

1.3.2 Synthesis of Diels-Alder product **6a** – **6d** ---------- S3

1.3.3 One-pot Diels-Alder and Stille coupling to obtain **7a** – **7e**, **8a, 9a** ---------- S4

1.3.4 One-pot Diels-Alder and Suzuki coupling to obtain **8b** – **8d** ---------- S4

2. Control reactions ---------- S5

2.1 Control reaction with the ligand only (no metal present): ---------- S5

2.2 Control reaction with Pd(dppe)Cl_2 (no boron in the ligand): ---------- S5

2.3 Control reaction with only catalyst (no acid initiator): ---------- S6

2.4 Control reaction with only acid-initiator (no catalyst): ---------- S6

2.5 Control reaction with (^{MeO}TBDPhos)Pd($S_2C_6H_4$) (no chloride bound to metal): ---------- S6

3. Analytical data for compounds ---------- S7

4. Supporting information references ---------- S16

5. NMR spectra ---------- S17

1. Representative synthetic procedures

1.1 General considerations

Unless otherwise noted, all reactions were performed in oven-dried glassware under nitrogen with solvents that were freshly distilled or dried using a Glass Contour Solvent Purification System (Pure Process Technology) and stored over molecular sieves. (^{R}TBDPhos)PdCl$_2$ complexes with R = Ph, MeO, ^{i}PrO, and F-^{i}PrO and (^{MeO}TBDPhos)Pd(S$_2$C$_6$H$_4$) were synthesized as described previously [s1] and stored in a glovebox prior to use. All commercial reagents were used without further purification unless otherwise indicated.

Catalytic reactions were magnetically stirred and monitored by thin layer chromatography carried out on 0.25 mm E. Merck silica gel plate (60$_f$ - 254) using UV light for visualization followed by $KMnO_4$ as a TLC stain. Separation of mixtures was performed by flash chromatography using silica gel (60 Å pore size) with the denoted solvent system.

^{1}H and ^{13}C NMR data were recorded on a Bruker AVANCE (300-MHz) or a Bruker AVANCE (500-MHz) NMR spectrometers using chloroform-*d* ($CDCl_3$) as the internal standards. All NMR chemical shifts are recorded in parts per million (PPM) relative to the chloroform reference peaks at δ 7.26 and δ 77.0 for ^{1}H and ^{13}C NMR spectra, respectively. Coupling constants are reported in hertz. ^{1}H NMR spectra are reported as chemical shift in ppm, followed by relative integral, multiplicity ("s" singlet, "bs" broad singlet, "d" doublet, "dd" doublet of doublets, "dt" doublet of triplet, "t" triplet, "q" quartet, "p" pentet, "m" multiplet), coupling constant where applicable, and assignment.

1.2 Preparation of (E)-4-bromopenta-2,4-dien-1-yl benzoate (1)

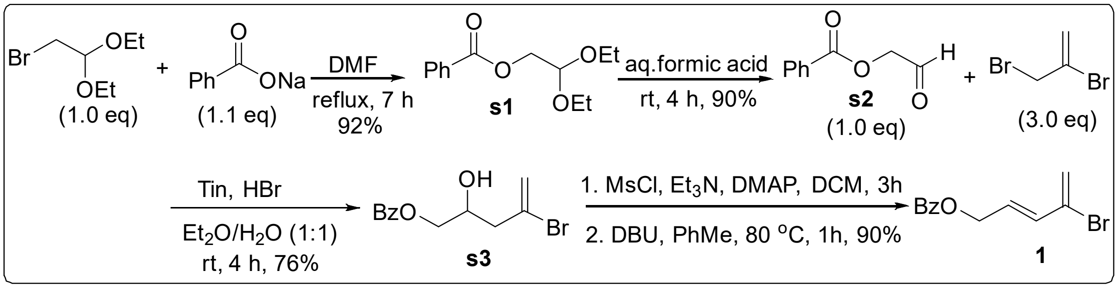

To a solution of 2-bromo-1,1-diethoxyethane (5 g, 3.8 ml, 25.37 mmol, 1.0 equiv.) in DMF (40 ml) was added sodium benzoate (2 g, 13.87 mmol, 0.55 equiv.) at room temperature. The reaction mixture was refluxed for 2 h followed by slow addition of additional sodium benzoate (2 g, 13.87 mmol, 0.55 equiv.) while refluxing. Reflux was then continued for 5 h. The mixture was allowed to cool to room temperature, worked up with ice-cold water (100 mL), and extracted using EtOAc (3 x 50 mL). The combined organic layers were washed with brine, dried over $MgSO_4$, and concentrated under reduced pressure. The residue was dried azeotropically with toluene (2 x 20 mL) to give benzoyloxy

acetaldehyde diethyl acetal **s1** as a slightly dark oil (5.5 g, 92%),[s2] which was used directly in the next reaction without purification.

A solution of crude acetal **s1** (33.5 mmol, 5.5 g) in aqueous formic acid (HCO_2H/H_2 = 8/2 v/v, 55 mL) was stirred at room temperature for 4 h. The reaction mixture was worked up with water (50 ml) and extracted using DCM (3 x 50 ml). The combined organic layers were washed with brine, dried over $MgSO_4$, and concentrated under reduced pressure to give aldehyde **s2** (3.4 g, 90%), which was used for the next step without purification.

To a suspension of Sn powder (3.69 g, 31.07 mmol, 1.5 equiv.) in a mixture of Et_2O/H_2O (1:1 v/v, 35 mL) was added 2,3-dibromopropene **4** (12.43 g, 6.76 mL, 62.19 mmol, 3 equiv.) and aldehyde **s2** (3.4 g, 20.7 mmol, 1.0 equiv.) at room temperature. The resulting mixture was vigorously stirred for 5 min, cooled using an ice bath, and HBr (48% aq., 5.0 mL, 62.19 mmol, 3.0 equiv.) was added slowly. The reaction mixture was stirred vigorously for 4 h. Upon completion of the reaction, as monitored using TLC, it was filtered through a celite bed. The bed was washed using Et_2O (50 mL). The organic layer was separated, and the aqueous layer further extracted with Et_2O (2 x 50 ml). The combined organic layers were washed with brine, dried over anhydrous $MgSO_4$, and concentrated *in vacuo*. The crude product was purified by flash column chromatography using silica gel (eluent: 10% EA and hexane) to afford the desired alcohol **s3** (4.5 g, 76%) as a colorless oil. [s2]

To a solution of alcohol **s3** (4.5 g, 16 mmol, 1 equiv.) in CH_2Cl_2 (45 mL) was added Et_3N (4.8 g, 6.6 mL, 47 mmol, 3 equiv.) and DMAP (192.8 mg, 1.58 mmol, 0.1 equiv.) and stirred for 10 mins. The reaction mixture was cooled to 0 °C and MsCl (3.6 g, 2.4 mL, 32 mmol, 2 equiv.) was added dropwise. The resulting mixture was warmed to room temperature, stirred for 3 h, quenched with saturated aqueous NH_4Cl (40 mL), and extracted with CH_2Cl_2 (2 × 40 mL). The combined organic layers were washed with brine (30 mL), dried over anhydrous $MgSO_4$, and concentrated *in vacuo* to obtain the Ms-protected alcohol (5.7 g, 100%).[s2] The crude oil (1.0 g) was dissolved in toluene (10 mL) and DBU (1.26 mL, 8.25 mmol, 3.0 equiv.) was added. The resulting mixture was stirred at 80 °C for 1 h, cooled to room temperature, worked up with saturated aqueous NH_4Cl (10 mL), extracted using Et_2O (3 × 20 mL). The combined organic layers dried over anhydrous $MgSO_4$ and concentrated *in vacuo*. The crude products were purified by flash column chromatography using silica gel (eluent: ethylacetate and hexane) to afford the desired product **1** (660 mg, 90%) as colorless oil.[s3]

Note: The diene **1** was stored as a 0.2 M solution in CH_2Cl_2 at 4 °C because it polymerizes when stored neat at room temperature.

1.3 Standard catalytic procedures

1.3.1 Procedure for the synthesis of Diels-Alder product 3a – 3g

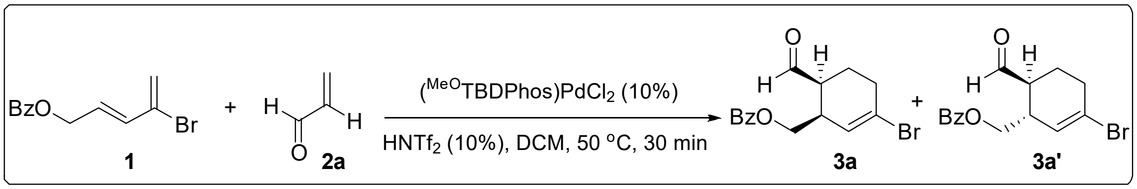

(MeOTBDPhos)$PdCl_2$ (57.5 mg, 0.11 mmol, 0.1 equiv.) was weighed out in an oven-dried catalytic tube in the glovebox and dissolved in dry and degassed DCM (1.0 ml). The DCM from the diene **1** (0.30 g, 1.15 mmol, 1.0 equiv.), which was stored at 4 °C as a 0.2 M solution, was removed under vacuum. It was then dissolved in degassed DCM (2.0 ml) and added to the solution of **1** under nitrogen atmosphere. The catalytic tube was sealed using a rubber septum and acrolein **2a** (193.2 mg, 3.44 mmol, 3.0 equiv.) was added. To this solution, $HNTf_2$ (32.2 mg, 0.11 mmol, 0.1 equiv.) dissolved in degassed DCM (2.0 mL) was added dropwise. The reaction mixture turned red and was added to an oil bath preheated to 50 °C for 30 min. Upon completion (as monitored by TLC), the reaction was cooled to room temperature, quenched with saturated $NaHCO_3$ solution (10 ml), extracted using DCM (2 x 10 ml), and concentrated to obtain crude product **3**. Column chromatography on silica column (ethyl acetate/hexane = 1/10) afforded an inseparable mixture of **3a** and **3a'** (450 mg, 37%, *endo*/*exo* 3:1) as a pale-yellow wax.

Diels-Alder cycloadducts **3b** – **3g** were prepared as described for **3a**.

1.3.2 Procedure for the synthesis of Diels-Alder product 6a – 6d

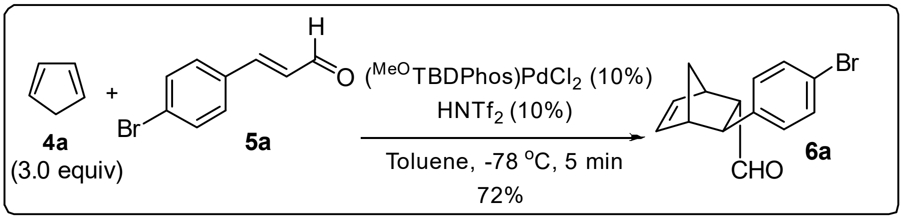

(MeOTBDPhos)$PdCl_2$ (57.5 mg, 0.11 mmol, 0.1 equiv.) was weighed out in oven-dried catalytic tube in the glovebox. 4-Bromocinnamaldehyde **5a** (0.30 g, 1.42 mmol, 1.0 equiv.) was dissolved in dry and degassed toluene (2.0 ml) and added to the tube under nitrogen atmosphere. The catalytic tube was sealed using a rubber septum and cyclopentadiene **4a** (0.28 g, 4.25 mmol, 3.0 equiv.) was added. The reaction mixture was allowed to cool to -78 °C and $HNTf_2$ (39.8 mg, 0.14 mmol, 0.1 equiv.) dissolved in degassed toluene (2.0 mL) was added dropwise. The reaction mixture turned dark purple and within 5 min was quenched with saturated $NaHCO_3$ solution (10 ml), extracted using EtOAc (2 x 10 ml), and concentrated to obtain crude product **3**. Column chromatography on silica column neutralized with Et_3N

(EtOAc/hexane = 1/20) afforded an inseparable mixture of **6a** and **6a'** (0.28 g, 72%, *endo*/*exo* 87:13) as a colorless wax.

Diels-Alder cycloadducts **6b** – **6d** were prepared as described for **6a** with the temperature of $HNTf_2$ addition being -20 °C for **6b** and 0 °C for **6c** and **6d**.

1.3.3 Procedure for one-pot Diels-Alder and Stille coupling to obtain 7a – 7e, 8a, and 9a

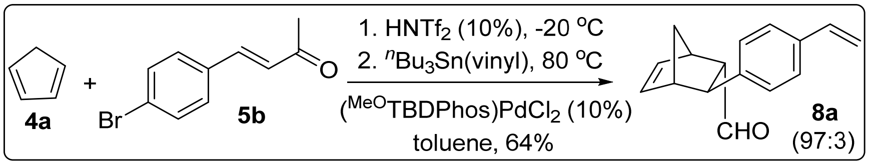

(^{MeO}TBDPhos)$PdCl_2$ (22.0 mg, 0.044 mmol, 0.1 equiv.) was weighed out in an oven-dried catalytic tube in the glovebox. *trans*-Benzylideneacetone **5b** (100.0 mg, 0.44 mmol, 1.0 equiv.) was dissolved in dry and degassed toluene (1.0 ml) and added to the tube under nitrogen atmosphere. The catalytic tube was sealed using a rubber septum and cyclopentadiene **4a** (88.0 mg, 1.33 mmol, 3.0 equiv.) was added. The reaction mixture was cooled to -20 °C and $HNTf_2$ (12.0 mg, 0.044 mmol, 0.1 equiv.) dissolved in degassed toluene (2.0 mL) was added dropwise. The reaction mixture turned dark purple and was monitored using TLC. Once complete consumption of the DA starting material was confirmed, $^{n}Bu_3Sn$(vinyl) (169 mg, 0.53, 1.2 equiv.) was added. The reaction was heated to 80 °C for 4 h, quenched using saturated aqueous KF solution (10 ml), and extracted using EtOAc (2 x 10 ml). The organic layer was further washed with KF solution (2 x 5 ml), water (10 ml), dried over $MgSO_4$, and concentrated under vacuum to obtain the crude product. Column chromatography on silica column neutralized with Et_3N (EtOAc/hexane = 1/20) afforded **8a** (68 mg, 64%, *endo*/*exo* 97:3) as a colorless oil.

Stille products **7a – 7e** and **9a** were prepared using the same procedure as described above.

1.3.4 Procedure for one-pot Diels-Alder and Suzuki coupling to obtain 8b – 8d

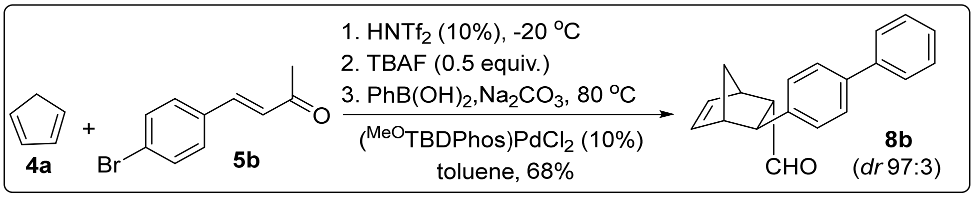

(^{MeO}TBDPhos)$PdCl_2$ (22.0 mg, 0.044 mmol, 0.1 equiv.) was weighed out in an oven-dried catalytic tube in the glove box. *trans*-Benzylideneacetone **5b** (100.0 mg, 0.44 mmol, 1.0 equiv.) was dissolved in dry and degassed toluene (1.0 ml) and added to the tube under nitrogen atmosphere. The catalytic tube was sealed using a rubber septum and cyclopentadiene **4a** (88.0 mg, 1.33 mmol, 3.0 equiv.) was added. The reaction mixture was allowed to cool to -20 °C and $HNTf_2$ (12.0 mg, 0.044 mmol, 0.1 equiv.) dissolved in degassed toluene (2.0 mL) was added dropwise. The reaction mixture

turned dark purple and was monitored using TLC. Once complete consumption of the DA starting material was confirmed, TBAF (58.1 mg, 0.22 mmol, 0.5 equiv.) dissolved in dry toluene (1 ml) was added and the mixture was stirred at room temperature for 15 min. $PhB(OH)_2$ (65.0 mg, 0.53 mmol, 1.2 equiv.) and Na_2CO_3 (56.0 mg, 0.53 mmol, 1.2 equiv.) dissolved in degassed water (1.0 ml) was then added. The reaction was heated to 80 °C for 1 h, quenched with water (10 ml), and extracted using EtOAc (2 x 10 ml). The organic layer was washed with brine (10 ml), dried over $MgSO_4$, and concentrated under vacuum to obtain the crude product. Column chromatography on silica column neutralized with Et_3N (EtOAc/hexane = 1/20) afforded **8b** (87.0 mg, 68%, *endo*/*exo* 97:3) as a white solid.

Suzuki products **8c** and **8d** were prepared using the same procedure as described above.

2. Control reactions

2.1 Control reaction with the ligand only (no metal present):

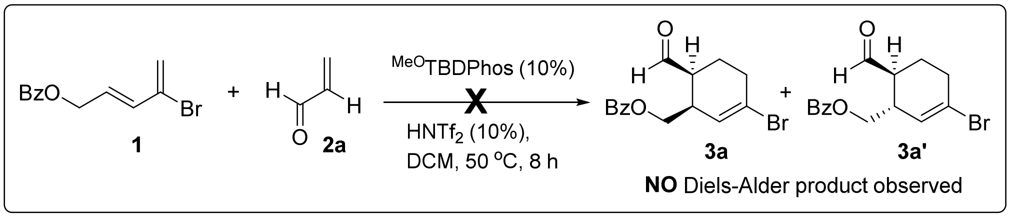

This control reaction was carried out with the ^{MeO}TBDPhos ligand without Pd. We wanted to test if the ligand itself could be protonated, without the metal present, and used as Lewis acid for the Diels-Alder reaction. The diene remained as such, and there was no formation of DA product even after 8 h of heating at 50 °C. This observation is in alignment with our prior studies showing how TBDPhos ligands decompose during attempted reactions at TBD unless the ligand is bound to a metal.[1d]

2.2 Control reaction with Pd(dppe)Cl_2 (no boron in the ligand):

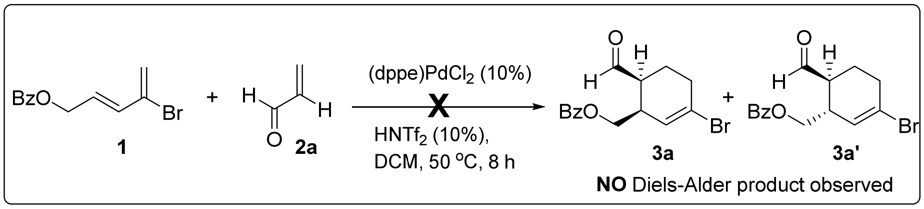

This control reaction was carried out with Pd(dppe)Cl_2 as the catalyst. We wanted to test if $PdCl_2$ complex containing a standard diphosphine ligand coordinated would catalyze the Diels-Alder reaction. The diene remained as such, and there was no formation of DA product even after 8 h of heating at 50 °C. This observation suggests the DA reaction does not occur at the metal and required the boron present in the ligand.

2.3 Control reaction with only catalyst (no acid initiator):

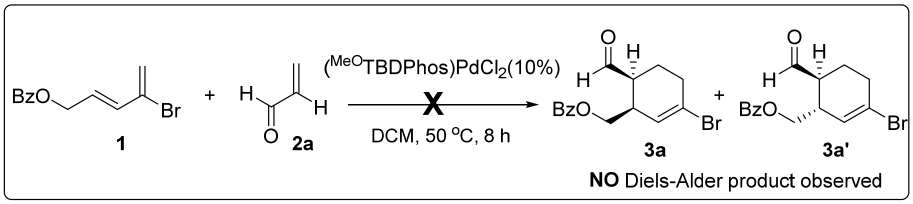

This control reaction was carried out with (^{MeO}TBDPhos)PdCl$_2$ as the catalyst without acid initiator. We wanted to test if (^{MeO}TBDPhos)PdCl$_2$ by itself would serve as a Lewis acid for the Diels-Alder reaction. The diene remained as such, and there was no formation of DA product even after 8 h of heating at 50 °C. This observation is in complete agreement with our studies about the protonation of the bridgehead nitrogen being the prerequisite for accessing reactivity at the borane.[1d]

2.4 Control reaction with only acid-initiator (no catalyst):

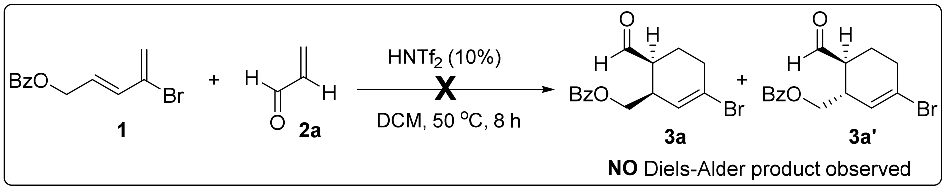

This control reaction was carried out with only the acid initiator HNTf$_2$ (10%). We wanted to test if HNTf$_2$ by itself would serve to catalyze the Diels-Alder reaction. The diene remained as such, and there was no formation of DA product even after 8 h of heating at 50 °C. This observation suggests that both the catalyst and acid initiator are required for the DA cycloaddition.

2.5 Control reaction with (^{MeO}TBDPhos)Pd($S_2C_6H_4$) (no chloride bound to metal):

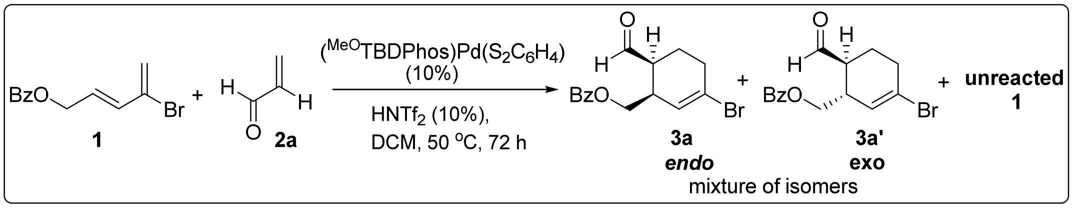

This control reaction was carried out with (^{MeO}TBDPhos)Pd($S_2C_6H_4$) (10%) and acid initiator HNTf$_2$ (10%). We wanted to test if the DA reaction would proceed with a strongly bound dithiolate coordinated to the metal. ^{1}H NMR data collected on the crude reaction mixture after 21 h revealed the expected resonances for **3a** and **3a′**, which continued to grow in when checked again at day 2 and when the reaction was stopped at 72 h. ^{1}H NMR analysis revealed that approximately half of the starting material **1** remained when the reaction was stopped.

3. Analytical data for compounds

Spectral data for (3-bromo-6-formylcyclohex-2-en-1-yl)methyl benzoate (3a)

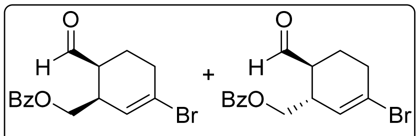

3a *endo* **3a'** *exo*

(*dr 75:3*)

Colorless wax, (45.0 mg, 0.14 mmol, 37%); ^{1}H NMR (500 MHz, $CDCl_3$): major isomer δ 9.86 (s, 1H), 8.01–7.96 (m, 2H), 7.59–7.55 (m, 1H), 7.47–7.43 (m, 2H), 6.14–6.13 (m, 1H), 4.45 (dd, *J* = 11.5, 5.4 Hz, 1H), 4.28-4.23 (m, 1H), 3.23–3.18 (m, 1H), 2.78–2.74 (m, 1H), 2.61–2.50 (m, 2H), 2.10–2.02 (m, 2H); ^{13}C NMR (125 MHz, $CDCl_3$): δ 201.6, 166.0, 133.3, 129.6, 129.5, 128.5, 126.7, 125.2, 63.5, 47.5, 38.0, 33.7, 20.7, two peaks are merged. ^{1}H NMR (500 MHz, $CDCl_3$): minor isomer **3a**' δ 9.72 (d, *J* = 1.4 Hz), 6.08 (p, 1H), 4.34 (dd, *J* = 11.0, 5.7 Hz, 1H), 3.14–3.11 (m, 1H), 1.97–1.91 (m, 1H); ^{13}C NMR (125 MHz, $CDCl_3$): 202.0, 166.2, 133.2, 129.4, 128.4, 126.7, 65.8, 47.1, 37.0, 33.2, 22.5, other peaks are merged.

Spectral data for 3-bromo-6-(methoxycarbonyl)cyclohex-2-en-1-yl)methyl benzoate (3b)

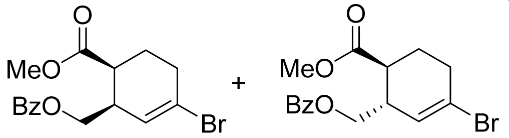

3b **3b'**

(*dr* 62:38)

Colorless oil, (53 mg, 0.15 mmol, 40%); ^{1}H NMR (500 MHz, $CDCl_3$): δ 8.05–8.00 (m, 2H), 7.59–7.55 (m, 1H), 7.47–7.43 (m, 2H), 6.13–6.11 (m, 1H), 4.31 (d, *J* = 8.2 Hz, 1H), 4.26 (dd, *J* = 8.2, 3.5 Hz, 1H), 3.57 (s, 3H), 3.15- 3.09 (m, 1H), 2.86- 2.81 (m, 1H), 2.58–2.50 (m, 2H), 2.05-1.96 (m, 2H); ^{13}C NMR (125 MHz, $CDCl_3$): δ 179.4, 166.3, 133.2, 130.0, 129.7, 128.5, 127.3, 124.5, 64.5, 51.9, 40.0, 38.6, 34.2, 23.0, two peaks are merged. ^{1}H NMR (500 MHz, $CDCl_3$): minor isomer **3b**' δ 6.04 – 6.02 (m, 1H), 4.56- 4.52 (m, 1H), 4.38- 4.34 (m, 1H) 3.63 (s, 3H); ^{13}C NMR (125 MHz, $CDCl_3$): most of the peaks are merged.

Spectral data for ((1S,6S)-3-bromo-6-cyanocyclohex-2-en-1-yl)methyl benzoate (3c)

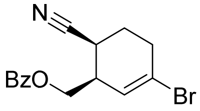

3c *endo*

White solid, (86 mg as *endo/exo* mixture, 0.27 mmol, 70%); ^{1}H NMR (500 MHz, $CDCl_3$): δ 8.07-8.03 (m, 2H), 7.62-7.56 (m, 1H), 7.49–7.43 (m, 2H), 5.98 (q, J = 3.5 Hz, 1H), 4.56-4.51 (m, 1H) 4.41- 4.34 (m, 1H), 3.25-3.20 (m, 1H), 3.01- 2.94 (m, 1H), 2.88-2.75 (m, 1H), 2.61- 2.51 (m, 1H), 2.27–2.17 (m, 1H), 2.06- 1.95 (m, 1 H); ^{13}C NMR (125 MHz, $CDCl_3$): δ 166.0, 133.4, 129.7, 129.4, 128.5, 125.0, 124.0, 118.6, 64.8, 38.9, 32.0, 26.9, 25.9, two peaks are merged.

Spectral data for ((1S,6R)-3-bromo-6-cyanocyclohex-2-en-1-yl)methyl benzoate (3c')

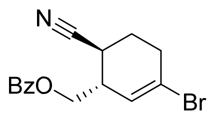

3c' *exo*

White solid, (86 mg as *endo/exo* mixture, 0.27 mmol, 70%); ^{1}H NMR (500 MHz, $CDCl_3$): δ 8.05-8.03 (m, 2H), 7.61-7.58 (m, 1H), 7.49–7.45 (m, 2H), 6.03 (q, J = 1.7 Hz, 1H), 4.41-4.34 (m, 2H), 3.00-2.95 (m, 1H), 2.85- 2.81 (m, 1H), 2.70- 2.63 (m, 1H), 2.61- 2.53 (m, 1H), 2.25–2.17 (m, 1H), 2.11-2.05 (m, 1H); ^{13}C NMR (125 MHz, $CDCl_3$): δ 166.1, 133.4, 129.7, 129.3, 128.5, 125.6, 124.0, 120.6, 64.8, 40.8, 33.0, 26.6, 25.6, two carbon peaks are merged.

Spectral data for ((1S,6S)-6-acetyl-3-bromocyclohex-2-en-1-yl)methyl benzoate (3d)

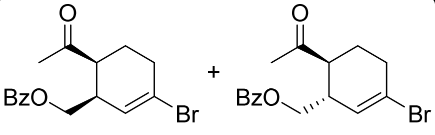

3d (*dr* 91:9) **3d'**

White solid, (80 mg, 0.24 mmol, 62%); ^{1}H NMR (500 MHz, $CDCl_3$): δ 7.98- 7.96 (m, 2H), 7.58-7.55 (m, 1H), 7.46–7.43 (m, 2H), 6.14 -6.12 (m, 1H), 4.34 -4.31 (m, 1H), 4.20 – 4.16 (m, 1H), 3.18- 3.13 (m, 1H), 2.87- 2.83 (m, 1H), 2.58- 2.48 (m, 2 H), 2.19 (s, 3H), 2.08- 1.98 (m, 1 H), 1.96- 1.90 (m, 1H); ^{13}C NMR (125 MHz, $CDCl_3$): δ 208.7, 166.1, 133.2, 129.7, 129.6, 128.5, 127.6, 127.1, 63.6, 47.8, 38.9, 34.4, 29.0, 21.7, two carbon peaks are merged. ^{1}H NMR (500 MHz, $CDCl_3$): minor isomer **3d**' δ 6.06-6.04 (m, 1H), 4.27- 4.23 (m, 1H), 4.16- 4.13 (m, 1H), 2.20 (s, 3H), ^{13}C NMR (125 MHz, $CDCl_3$): δ 209.5, 127.7, 124.8, 122.8, 66.3, 38.9, 34.2, 26.5, other peaks are merged.

Spectral data for 3-bromo-6-formyl-6-methylcyclohex-2-en-1-yl)methyl benzoate (3e)

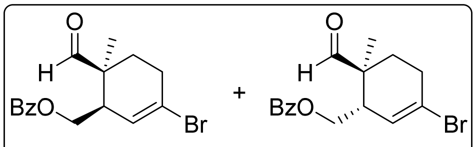

3e (*dr* 79:21) **3e'**

Colourless solid, (85 mg, 0.25 mmol, 66%); ^{1}H NMR (500 MHz, $CDCl_3$): δ 9.70 (s, 1H), 8.00-7.98 (m, 2H), 7.60-7.56 (m, 1H), 7.48–7.44 (m, 2H), 6.12- 6.10 (m, 1H), 4.44-4.38 (m, 1H), 4.34- 4.30 (m, 1H), 2.73- 2.70 (m, 1H), 2.63- 2.56 (m, 1H), 2.55- 2.51 (m, 1H), 2.07 (p, *J* = 6.8 Hz, 1 H), 1.75- 1.70 (m, 1H), 1.23 (s, 3H); ^{13}C NMR (125 MHz, $CDCl_3$): δ 203.5, 166.1, 133.3, 129.6, 128.5, 126.4, 123.8, 63.7, 45.9, 44.9, 40.7, 32.1, 28.8, 19.7, two carbon peaks are merged. ^{1}H NMR (500 MHz, $CDCl_3$): minor isomer **3e**' δ 9.54 (s, 1H), 7.60–7.56 (m, 1H), 6.01–6.00 (m, 1H), 4.31-4.27 (m, 1H), 4.21-4.17 (m, 1H), 3.15–3.11 (m, 1H), 1.95 -1.89 (m, 1H), 1.64 – 1.59(m, 1H); 13C NMR (100 MHz; CDCl3): δ 204.2, 129.6, 129.5, 128.5, 126.2 , 64.1, 45.8, other peaks are merged.

Spectral data for ((4S)-6-bromo-1,3-dioxo-1,3,3a,4,7,7a-hexahydroisobenzofuran-4-yl)methyl benzoate (3f)

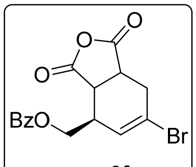

3f

White solid, (75 mg, 0.15 mmol, 54%); ^{1}H NMR (500 MHz, $CDCl_3$): δ 8.03-8.01 (m, 2H), 7.61-7.58 (m, 1H), 7.48–7.45 (m, 2H), 6.27 (dd, *J* = 3.9, 2.8 Hz, 1H), 4.76 (dd, *J* = 11.5, 7.0 Hz, 1H), 4.64 (dd, *J* = 11.5, 7.2 Hz, 1H), 3.60-3.58 (m, 2H), 3.10 (d, *J* = 2.1 Hz, 1H), 3.07 (d, *J* = 1.3 Hz, 1H), 2.98–2.93 (m, 1H); ^{13}C NMR (125 MHz, $CDCl_3$): δ 172.2, 170.4, 166.1, 133.4, 129.7, 129.4, 129.0, 128.5, 120.4, 63.4, 41.1, 40.9, 37.4, 33.6, two carbon peaks are merged; ESI-(MS+Na) calcd. for $C_{16}H_{13}BrO_5$: 386.9844; Found: 386.9838.

Spectral data for (R)-(3-bromo-5,8-dihydroxy-1,4-dihydronaphthalen-1-yl)methyl benzoate (3g)

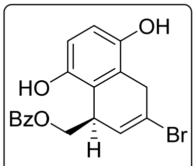

3g

White solid, (102 mg, 0.28 mmol, 71%); ^{1}H NMR (500 MHz, $CDCl_3$): δ 7.90-7.89 (m, 2H), 7.58-7.54 (m, 1H), 7.44–7.41 (m, 2H), 6.81 (d, *J* = 11 Hz, 1H), 6.77 (d, *J* = 11 Hz, 1H), 6.25–6.24 (m, 1H), 4.55 (dd, *J* = 11.0, 4.4 Hz, 1H), 4.43 (dd, *J* = 11.0, 3.6 Hz, 1H), 3.97–3.93 (m, 1H), 3.51–3.45 (m, 1H), 3.33–3.27 (m, 1H), two OH protons; ^{13}C NMR (125 MHz, $CDCl_3$): δ 185.7, 166.2, 140.4, 138.0, 136.8, 136.5, 136.1, 133.3, 129.5, 129.4, 128.5, 126.2, 119.4, 65.5, 38.1, 33.4, two carbon peaks are merged.

Spectral data for (1R,2S,3S,4S)-3-(4-bromophenyl)bicyclo[2.2.1]hept-5-ene-2-carbaldehyde (6a)

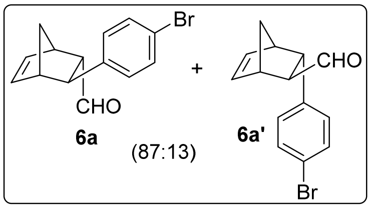

Pale-yellow wax, (94 mg, 0.34 mmol, 72%); ^{1}H NMR (500 MHz, $CDCl_3$): δ 9.59 (d, J = 2.1 Hz, 2H), 7.42 (d, J = 8.5 Hz, 1H), 7.13 (d, J = 8.2 Hz, 2H), 6.41 (dd, J = 8.9, 3.2 Hz, 1H), 6.17 (dd, J = 5.7, 2.8 Hz, 1H), 3.35 (bs, 1H), 3.09 (bs, 1H), 3.04 (d, J = 4.6 Hz, 1H), 2.92- 2.90 (m, 1H), 1.75 (d, J = 8.8 Hz, 1 H), 1.65-1.62 (m, 1H); ^{13}C NMR (125 MHz, $CDCl_3$): δ 202.9, 142.6, 139.1, 133.8, 131.6, 131.2, 129.1, 120.0, 61.0, 48.2, 47.1, 45.0, two carbon peaks are merged. ^{1}H NMR (500 MHz, $CDCl_3$): minor isomer **6a'** δ 9.90 (d, J = 2.0 Hz, 2H), 7.36 (d, J = 8.5 Hz, 1H), 7.01 (d, J = 8.2 Hz, 2H), 6.35 (dd, J = 8.9, 3.2 Hz, 1H), 6.05 (dd, J = 5.7, 2.8 Hz, 1H), 3.70 (bs, 1H), 3.23 (bs, 1H), 3.18 (d, J = 4.6 Hz, 1H), 2.54- 2.52 (m, 1H), 1.58-1.57 (m, 1H); ^{13}C NMR (125 MHz, $CDCl_3$): δ 202.2, 136.5, 129.6, 59.5, 48.3, 47.5, 45.2, other peaks are merged.

Spectral data for 1-((1R,2S,3S,4S)-3-(4-bromophenyl)bicyclo[2.2.1]hept-5-en-2-yl)ethan-1-one (6b)

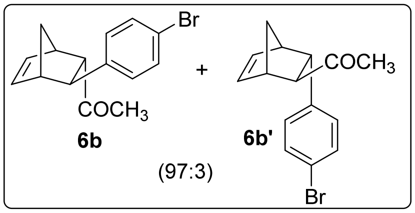

Pale-yellow wax, (366 mg, 1.26 mmol, 94%); ^{1}H NMR (500 MHz, $CDCl_3$): δ 7.40 (d, J = 14.2 Hz, 2H), 7.13 (d, J = 13.9 Hz, 2H), 6.39 (dd, J = 9.3, 5.4 Hz, 1H), 6.02 (dd, J = 9.5, 4.6 Hz, 1H), 3.34 (bs, 1H), 3.16- 3.14 (m, 1H), 2.99- 2.96 (m, 2H), 2.16 (s, 3H). 1.80 (d, J = 14.4 Hz, 1 H), 1.63-1.60 (m, 1H); ^{13}C NMR (125 MHz, $CDCl_3$): δ 207.6, 143.5, 139.4, 133.1, 131.5, 129.2, 119.7, 61.3, 48.2, 47.6, 46.5, 44.6, 29.0, two carbon peaks and peaks of the minor isomers are merged.

Spectral data for 1-(4'-bromo-5-methyl-1,2,3,6-tetrahydro-[1,1'-biphenyl]-2-yl)ethan-1-one 6c

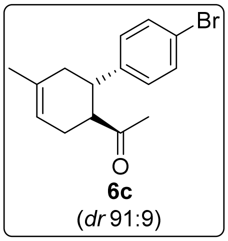

Colourless wax, (92 mg, 0.31 mmol, 88%); ^{1}H NMR (500 MHz, $CDCl_3$): δ 7.40 (d, J = 5.8 Hz, 2H), 7.07 (d, J = 8.4 Hz, 2H), 5.46 (m, 1H), 3.05- 2.99 (m, 1H), 2.95- 2.90 (m, 1H), 2.25-2.10 (m, 2H), 2.20-2.12 (m, 2H), 1.88 (s, 3H), 1.69 (s, 3H); ^{13}C NMR (125 MHz, $CDCl_3$): δ 211.7, 143.4, 133.6, 131.7, 129.2, 120.2, 118.9, 52.8, 42.3, 38.4, 29.7, 28.8, 23.1, two carbon peaks are merged; ESI (MS+H) calcd. for $C_{15}H_{18}BrO_5$: 293.0541; Found: 293.0538

Spectral data 1-(4'-bromo-4,5-dimethyl-1,2,3,6-tetrahydro-[1,1'-biphenyl]-2-yl)ethan-1-one (6d)

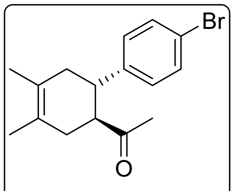

6d
(*dr* >99:1)

Colourless wax, (137 mg, 0.45 mmol, 82%); ^{1}H NMR (500 MHz, $CDCl_3$): δ 7.40 (d, J = 14.1 Hz, 2H), 7.06 (d, J = 14.1 Hz, 2H), 3.00- 2.95 (m, 2H), 2.23- 2.09 (m, 4H), 1.88 (s, 3H), 1.67 (s, 3H), 1.63 (s, 3H); ^{13}C NMR (125 MHz, $CDCl_3$): δ 211.5, 143.3, 131.6, 129.1, 125.2, 123.8, 120.2, 53.8, 42.7, 40.2, 34.9, 29.6, 18.6, 18.6, two carbon peaks are merged; ESI (MS+H) calcd. for $C_{16}H_{20}BrO$: 307.0698; Found: 307.0693

Spectral data ((3R)-4-formyl-3,4,5,6-tetrahydro-[1,1'-biphenyl]-3-yl)methyl benzoate (7a)

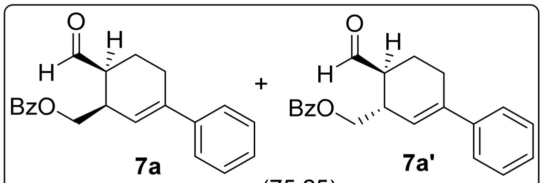

(75:25)

Colourless wax, (72 mg, 0.22 mmol, 58%); ^{1}H NMR (500 MHz, $CDCl_3$): δ 9.81 (d, J = 1.8 Hz, 1H), 8.02- 7.99 (m, 3 H), 7.58- 7.55 (m,1H), 7.47 -7.43 (m,1H), 7.39- 7.37 (m, 2H), 7.34 -7.31 (m, 2H), 7.28-7.26 (m, 1H), 6.04- 6.03 (m, 1H), 4.47 (dd, J = 5.4, 11.0, 1H), 4.37- 4.25 (m, 2H), 3.27 -3.22 (m, 1H), 2.56 – 2.53 (m, 2H), 2.18 (m, 1H), 1.98 -1.91 (m, 1H); ^{13}C NMR (125 MHz, $CDCl_3$): δ 203.2, 166.4, 141.1, 133.1, 129.8, 129.6, 128.5, 128.4, 128.3 127.4, 125.3, 122.1, 67.0, 47.1, 35.4, 26.0, 21.7, four peaks are merged.^{1}H NMR (500 MHz, $CDCl_3$): minor isomer **7a'** δ 9.72 (d, J = 1.8 Hz, 1H), 6.08- 6.05 (m, 1H), 3.16 -3.10 (m, 1H), 2.60 – 2.58 (m, 2H), 2.11-2.04 (m, 1H); ^{13}C NMR (125 MHz, $CDCl_3$): δ 201.9, 133.2, 129.8, 126.8, 65.8, 37.0, 22.5 other peaks are merged.

Spectral data for ((1R)-6-formyl-3-vinylcyclohex-2-en-1-yl)methyl benzoate (7b)

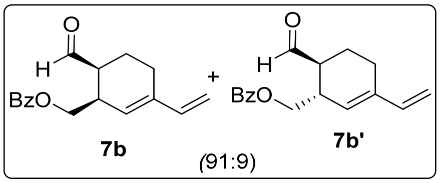

Colourless oil, (56 mg, 0.21 mmol, 42%); ^{1}H NMR (500 MHz, $CDCl_3$): δ 9.75 (s, 1 H), 8.00 (d, J = 7.6 Hz, 2 H), 7.57 (t, J = 7.3 Hz, 1H), 7.44 (t, J = 7.7Hz, 2H), 7.37 (dd, J = 17.6, 10.7 Hz, 1 H), 5.69 (s, 1 H), 5.16 (d, J = 17.6 Hz, 1 H), 5.03 (d, J = 10.7, 1H), 4.40 -4.37 (m, 1H), 4.26- 4.22(m, 1H), 3.17 (bs, 1H), 2.54 (bs, 1H), 2.33- 2.30 (m, 1H), 2.25 -2.20(m, 1H), 2.08 – 2.04 (m,1H), 1.86 -1.79(m,1H); ^{13}C NMR (125 MHz, $CDCl_3$): δ 203.2, 166.4, 138.8, 137.9, 133.1, 129.9, 129.6, 128.4, 126.4, 112.3, 66.8, 9.0, 35.3, 22.2, 21.1, two peaks are merged. ^{1}H NMR (500 MHz, $CDCl_3$): minor isomer **7b'** δ 9.89 (s, 1 H), 5.78 (s, 1 H), 4.49 -4.46 (m, 1H), 2.75 (bs, 1H); carbon peaks are merged.

Spectral data for ((1S,6S)-3-phenyl-6-cyanocyclohex-2-en-1-yl)methyl benzoate (7c)

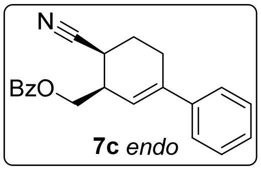

White solid, (62 mg as *endo/exo* mixture, 0.19 mmol, 60%); ^{1}H NMR (500 MHz, $CDCl_3$): δ 8.08- 8.05 (m, 2 H), 7.58 (tt, J = 12.3 Hz, J = 2.1 Hz, 1 H), 7.46 (t, J = 13.0 Hz, 2 H), 7.38-7.27 (m, 5H), 5.98-5.96 (m, 1 H), 4.53- 4.40 (m, 2 H), 3.15- 3.06 (m, 1 H), 2.92- 2.85 (m, 1 H), 2.74-2.64 (m, 1 H), 2.60-2.49 (m, 1 H), 2.36- 2.27 (m, 1 H), 2.17- 2.05 (m, 1 H); ^{13}C NMR (125 MHz, $CDCl_3$): δ 166.3, 140.5, 139.5, 133.3, 129.7, 129.6, 129.5, 128.5, 128.4, 127.8, 125.3, 120.8, 115.3, 65.8, 39.2, 27.6, 25.6, 24.7 three peaks are merged. EI+ calcd. for $C_{21}H_{19}NO_2$: 317.1416; Found: 317.1400

Spectral data for ((1S,6R)-3-phenyl-6-cyanocyclohex-2-en-1-yl)methyl benzoate (7c')

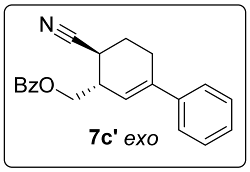

White solid, (62 mg as *endo/exo* mixture, 0.19 mmol, 60%); ^{1}H NMR (500 MHz, $CDCl_3$): δ 8.09- 8.05 (m, 2 H), 7.59 (tt, J = 12.2 Hz, J = 2.2 Hz, 1 H), 7.42 (t, J = 13.7 Hz, 2 H), 7.42-7.27 (m, 5H), 5.91-5.89 (m, 1 H), 4.70- 4.64 (m, 1 H), 4.45- 4.38 (m, 1 H), 3.32- 3.27 (m, 1 H), 3.14- 3.06 (m, 1 H), 2.88-2.75 (m, 1 H), 2.62- 2.53 (m, 1 H), 2.39- 2.29 (m, 1 H), 2.08- 1.97 (m, 1 H); ^{13}C NMR (125 MHz,

CDCl3): δ 166.2, 140.6, 139.3, 133.3, 129.7, 129.6, 128.5, 128.4, 127.8, 125.3, 119.9, 119.4, 65.8, 37.3, 27.8, 24.7 five peaks are merged.

Spectral data for 3-vinyl-6-formyl-6-methylcyclohex-2-en-1-yl)methyl benzoate (7d)

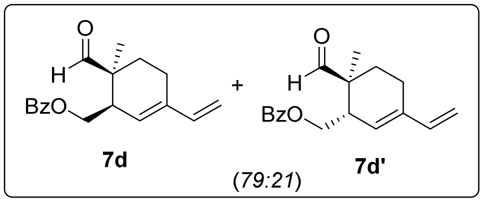

Colourless oil, (43 mg, 0.15 mmol, 45%); ^{1}H NMR (500 MHz, $CDCl_3$): δ 9.74 (s, 1H), 8.00-7.97 (m, 2H), 7.58-7.55 (m, 1H), 7.46–7.43 (m, 2H), 6.42- 6.36 (m, 1H), 5.77- 5.76 (m, 1 H), 5.18 (d, *J* = 17.6 Hz, 1H), 5.04 (d, *J* = 10.8 Hz, 1 H), 4.44- 4.41 (m, 1 H), 4.36- 4.33 (m, 1 H), 2.76- 2.74 (m, 1 H), 2.36-2.31 (m, 1 H), 2.27 -2.20 (m, 1 H), 2.05- 2.00 (m, 1 H), 1.74 -1.69 (m, 1 H), 1.21 (s, 3 H); ^{13}C NMR (125 MHz, $CDCl_3$): δ 204.8, 166.2, 138.6, 137.2, 133.1, 129.8, 129.6, 128.5, 126.3, 112.4, 64.4, 47.0, 43.3, 27.4, 20.9, 19.9, two carbon peaks are merged. ^{1}H NMR (500 MHz, $CDCl_3$): minor isomer **3e**' δ 9.58 (s, 1H), 8.07–8.02 (m, 2H), 7.46- 7.43 (m, 2H), 6.41- 6.34)m, 1 H), 5.61- 5.60 (m, 1 H), 4.88-4.84 (m, 1H), 4.80- 4.75 (m, 1 H), 4.17- 4.13 (m, 1 H), 3.20–3.17 (m, 1H), 1.85 -1.79 (m, 1H), 1.63 – 1.59 (m, 1H), 1.1 (s, 3 H); 13C NMR (100 MHz; CDCl3): δ 205.2, 166.3, 137.3, 64.9, 38.9, 28.5, 20.1 other peaks are merged.

Spectral data 1-((1S,2R,4R)-3-(4-vinylphenyl)bicyclo[2.2.1]hept-5-en-2-yl)ethan-1-one (8a)

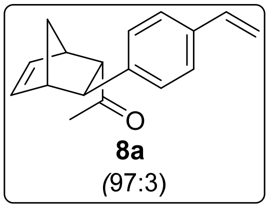

Colourless oil, (106 mg, 0.45 mmol, 64%); ^{1}H NMR (500 MHz, $CDCl_3$): δ 7.34 (d, *J* = 8.5 Hz, 2H), 7.23 (d, *J* = 8.2 Hz, 1H), 6.69 (dd, *J* = 17.6, 10.9 Hz, 1H), 6.40 (dd, *J* = 5.7, 3.3 Hz, 1H), 6.03 (dd, *J* = 5.7, 2.8 Hz, 1 H), 5.71 (dd, *J* = 17.6, 1.0 Hz, 1H) 5.21 (dd, *J* = 10.9, 0.9 Hz, 1H) 3.34 –3.32 (m, 1 H), 3.17 (dd, *J* = 5.0, 1.4 Hz, 1H), 3.05 (dd, *J* = 5.0, 3.5 Hz, 1H), 3.01- 2.99 (m, 1H), 2.15 (s, 3H), 1.85 – 1.83 (m, 1H), 1.62 – 1.60 (m,1H), 1.86 -1.79 (m,1H); ^{13}C NMR (125 MHz, $CDCl_3$): δ 207.9, 144.1, 139.3, 136.4, 135.4, 133.1, 127.6, 126.3, 113.3, 61.1, 48.6, 47.5, 46.5, 45.1, 29.1, two peaks are merged; ESI-(MS+Na) calcd. for $C_{17}H_{18}NaO$: 261.1255; Found: 261.1250.

Spectral data 1-((1S,2R,4R)-3-([1,1'-biphenyl]-4-yl)bicyclo[2.2.1]hept-5-en-2-yl)ethan-1-one (8b)

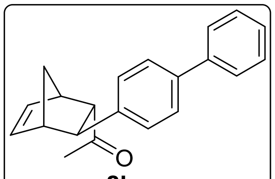

8b
(97:3)

White solid, (128 mg, 0.44 mmol, 68%); ^{1}H NMR (500 MHz, $CDCl_3$): δ 7.59- 7.57 (m, 2 H), 7.54- 7.52 (m, 2 H), 7.44 -7.41 (m, 2H), 7.35- 7.33 (m, 3H), 6.42 (dd, J = 5.5, 3.2, 1H), 6.04 (dd, J = 5.7, 2.7, 1H), 3.36 (bs, 1H), 3.24 -3.23 (m,1 H), 3.11b-3.10(m, 1H), 3.06 (bs, 1H), 2.18 (s, 3H), 1.90 (d, J = 8.6, 1H), 1.65 – 1.63 (m, 1H); ^{13}C NMR (125 MHz, $CDCl_3$): δ 208.0, 143.5, 140.9, 139.4, 138.9, 133.1, 128.7, 127.9, 127.2, 127.1, 127.0, 61.1, 48.6, 47.6, 46.5, 45.0, 29.1, four peaks are merged; ESI-(MS+Na) calcd. for $C_{12}H_{20}NaO$: 311.1412; Found: 311.1404.

Spectral data for 1-((1S,2R,4R)-3-(4'-methoxy-[1,1'-biphenyl]-4-yl)bicyclo[2.2.1]hept-5-en-2-yl)ethan-1-one (8c)

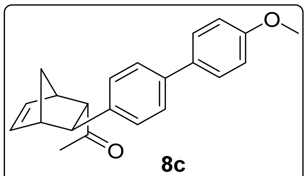

8c
(97:3)

White solid, (113 mg, 0.17 mmol, 52%); ^{1}H NMR (500 MHz, $CDCl_3$): δ 7.52- 7.79 (m, 4 H), 7.31 (d, *J* = 13.6 Hz, 2 H), 6.97 (d, *J* = 14.8 Hz, 2 H), 6.42 (dd, *J* = 9.2, 5.4 Hz, 1 H), 6.04 (dd, *J* = 9.4, 4.8 Hz, 1 H), 3.85 (s, 3H), 3.35 (bs, 1 H), 3.22 (d, *J* = 4.7 Hz, 1H), 3.11 -3.09 (m, 1H), 3.04 (bs, 1H), 2.17 (s, 3H), 1.89 (d, *J* = 8.7 Hz, 1H), 1.64 – 1.62 (m, 1H); ^{13}C NMR (125 MHz, $CDCl_3$): δ 208.0, 159.0, 142.9, 139.4, 133.1, 127.9, 127.8, 127.7, 126.7, 114.2, 114.1, 61.1, 55.3, 48.7, 47.6, 46.5, 45.0, 29.1, four peaks are merged; ESI (MS+Na) calcd. for $C_{22}H_{22}NaO_2$: 341.1517; Found: 341.1521.

Spectral data for 1-(5-methyl-1,2,3,6-tetrahydro-[1,1':4',1''-terphenyl]-2-yl)ethan-1-one (8d)

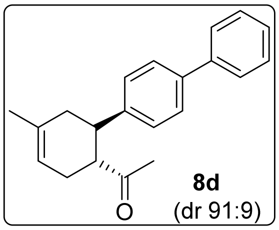

8d
(dr 91:9)

White solid, (149 mg, 0.51 mmol, 58%); ^{1}H NMR (500 MHz, $CDCl_3$): δ 7.58- 7.56 (m, 2 H), 7.53 (d, *J* = 8.3 Hz, 2 H), 7.43 (t, *J* = 7.4 Hz, 2 H), 7.33 (tt, *J* = 7.3, 1.4 Hz, 1 H), 7.27 (d, *J* = 8.1 Hz, 2 H), 5.50-

5.48 (m, 1H), 3.13- 3.07 (m, 1H), 3.03- 2.98 (m, 1H), 2.30- 2.28 (m, 2H), 2.24- 2.23 (m, 2H), 1.90 (s, 3H), 1.72 (s, 3H); ^{13}C NMR (125 MHz, $CDCl_3$): δ 212.2, 143.3, 139.4, 133.8, 129.2, 128.7, 127.8, 127.3, 127.1, 127.0, 118.9, 53.0, 46.6, 38.6, 29.7, 28.8, 23.1, four peaks are merged; EI (MS+.) calcd. for $C_{21}H_{22}O$: 290.1671; Found: 290.1660.

Spectral data for (1S,4R)-3-(4-vinylphenyl)bicyclo[2.2.2]oct-5-ene-2-carbaldehyde (9a)

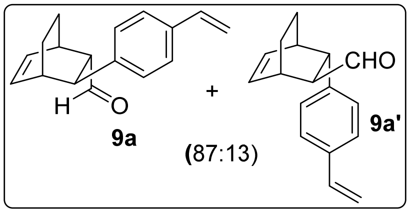

Colourless oil, (50 mg, 0.21 mmol, 45%); ^{1}H NMR (500 MHz, $CDCl_3$): δ 9.50 (d, *J* = 1.3 Hz, 1H), 7.39 (d, *J* = 8.3 Hz, 2 H), 7.24 (d, *J* = 8.2 Hz, 2H), 6.71 (dd, *J* = 17.6, 10.9 Hz, 1H), 6.52 (dt, *J* = 7.4, 1.3 Hz, 1H), 6.20 (dt, *J* = 7.4, 1.1 Hz, 1 H), 5.73 (dd, *J* = 17.6, 0.9 Hz, 1H) 5.23 (dd, *J* = 10.9, 0.9 Hz, 1H) 3.20 – 3.18 (m, 1 H), 3.09-3.07 (m, 1H), 2.83-2.82 (m, 1H), 2.63-2.61 (m, 1H), 1.73 – 1.71 (m, 1H), 1.50 – 1.44 (m, 1 H), 1.11-1.04 (m,1H), 0.90-0.86 (m, 1H); ^{13}C NMR (125 MHz, $CDCl_3$): δ 202.7, 141.7, 137.1, 136.4, 131.6, 130.9, 128.2, 126.3, 113.6, 56.0, 43.0, 36.6, 31.3, 25.6, 18.8, two peaks are merged; GCMS (MS+.) calcd. for $C_{17}H_{18}O$: 238.1358; Found: 238.1357. ^{1}H NMR (500 MHz, $CDCl_3$): minor isomer **9a'** δ 7.45 (d, *J* = 8.4 Hz, 2 H), 7.14 (d, *J* = 8.3 Hz, 2H), 2.78-2.74 (m, 1H); ^{13}C NMR (125 MHz, $CDCl_3$): δ 202.3, 136.9, 131.5, 129.7, 42.6, 36.4, 31.3, 29.7 other peaks are merged.

4. Supporting Information References

[s1] a) Culpepper, J. D.; Lee, K.; Daly, S. R. "Modular Synthesis of Diphosphoramidite Ligands Derived from 1,8,10,9-Triazaboradecalin and Their Complexes with Ni, Pd, and Pt." *Polyhedron*, **2022**, accepted. b) Fritz, P.; Niedenzu, K.; Dawson, J. W. *Inorg. Chem.* **1965**, *4*, 886-889. b) Rothgery, E. F.; Niedenzu, K. *Syn. Inorg. Metal-Org. Chem.* **1971**, *1*, 117-121. c) Lee, K.; Culpepper, J. D.; Parveen, R.; Swenson, D. C.; Vlaisavljevich, B.; Daly, S. R., Modifying Phosphorus(III) Substituents to Activate Remote Ligand-Centered Reactivity in Triaminoborane Ligands. *Organometallics* **2020,** *39* (13), 2526-2533. d) Lee, K.; Donahue, C. M.; Daly, S. R., Triaminoborane-bridged diphosphine complexes with Ni and Pd: coordination chemistry, structures, and ligand-centered reactivity. *Dalton Trans.* **2017,** *46* (29), 9394-9406.

[s2] Du, J. F.; Watanabe, K. A., Facile preparation of alpha-acyloxyacetaldehyde, a versatile intermediate in the synthesis of antiviral nucleosides. *Synthetic Commun.* **2004,** *34* (11), 1925-1930.

[s3] Choi, H.; Shirley, H. J.; Aitken, H. R. M.; Schulte, T.; Sohnel, T.; Hume, P. A.; Brimble, M. A.; Furkert, D. P., Intermolecular Diels-Alder Cycloaddition/Cross-Coupling Sequences of 2-Bromo-1,3-butadienes. *Org. Lett.* **2020,** *22* (3), 1022-1027.

^{1}H NMR data of compound **3a**
500 MHz

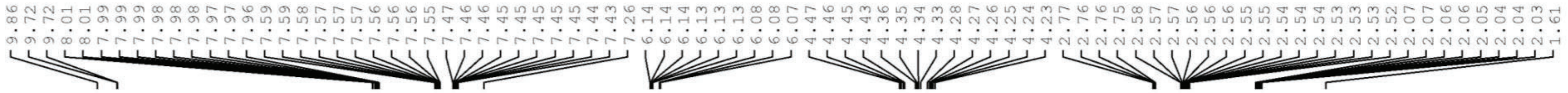

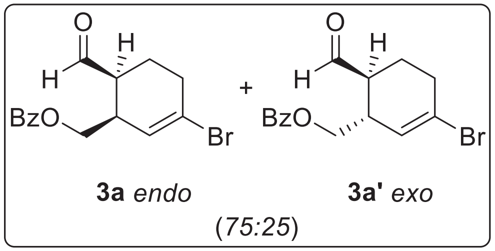

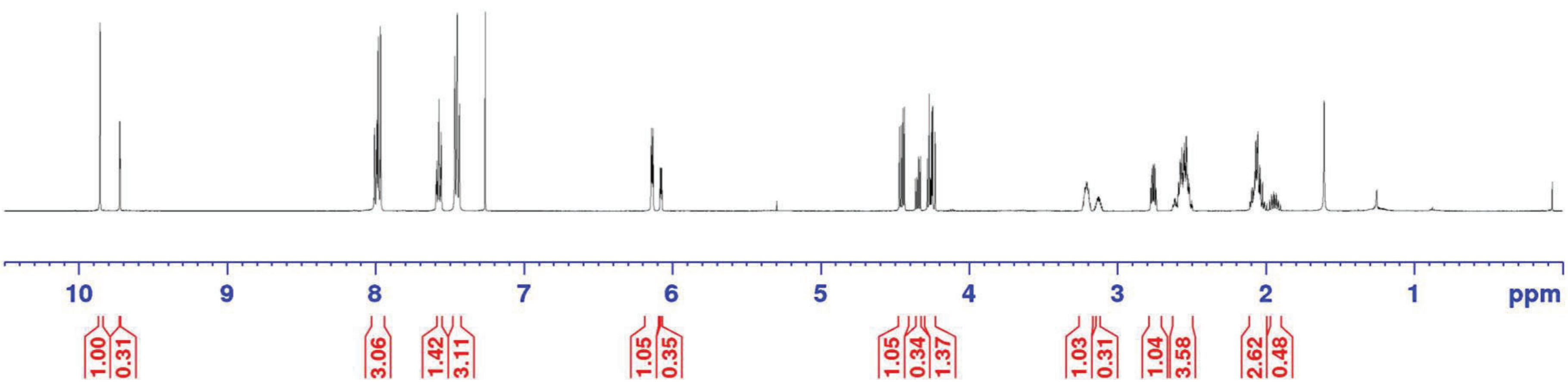

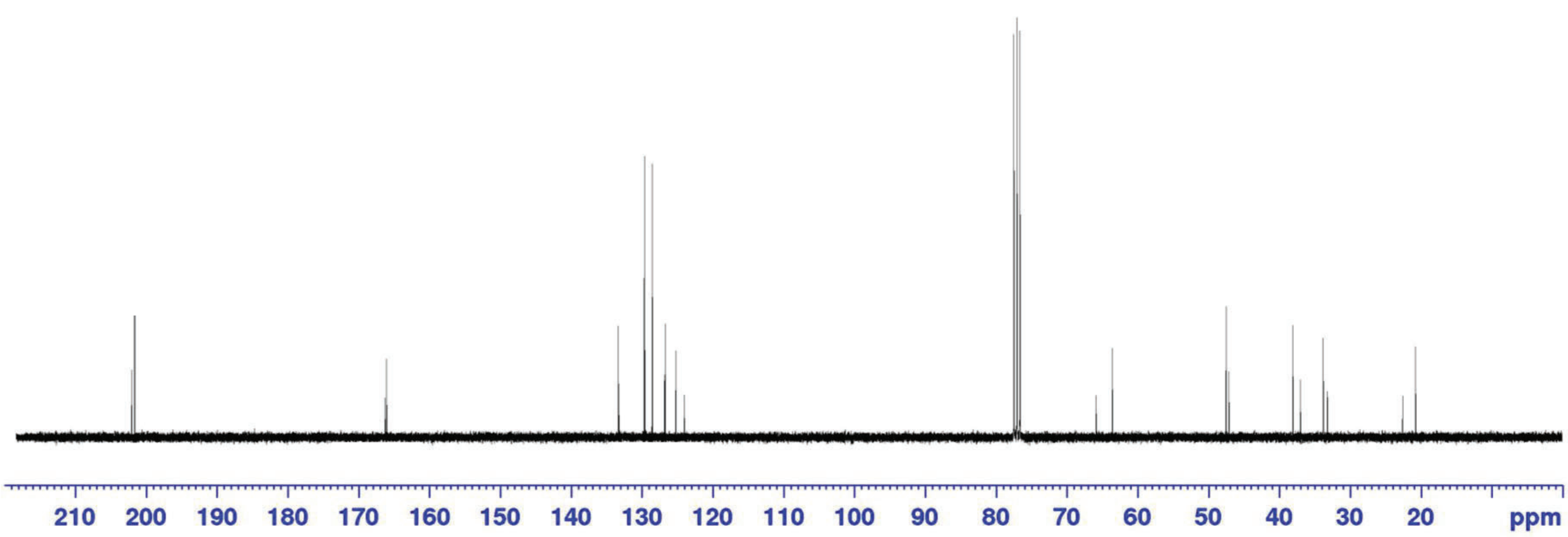

^{13}C NMR data of compound **3a**

500 MHz

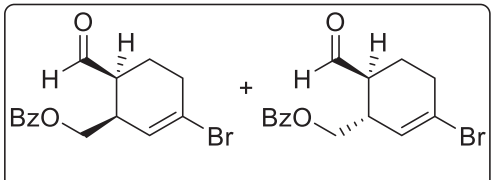

3a *endo* **3a'** *exo*

(75:25)

^{1}H NMR data of compound **3b**
500 MHz

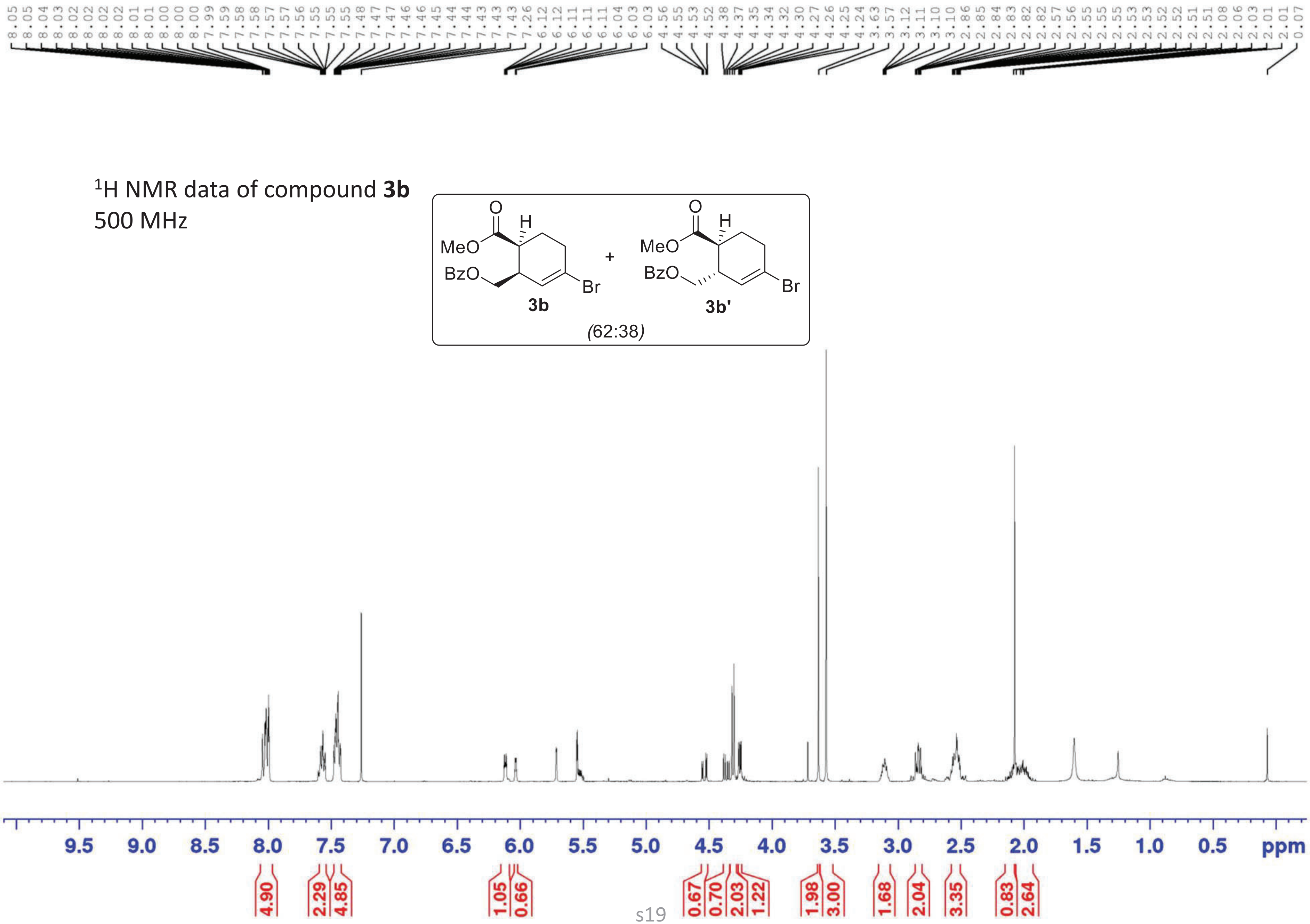

^{13}C NMR data of compound **3b**
500 MHz

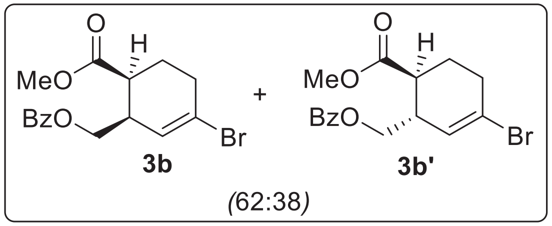

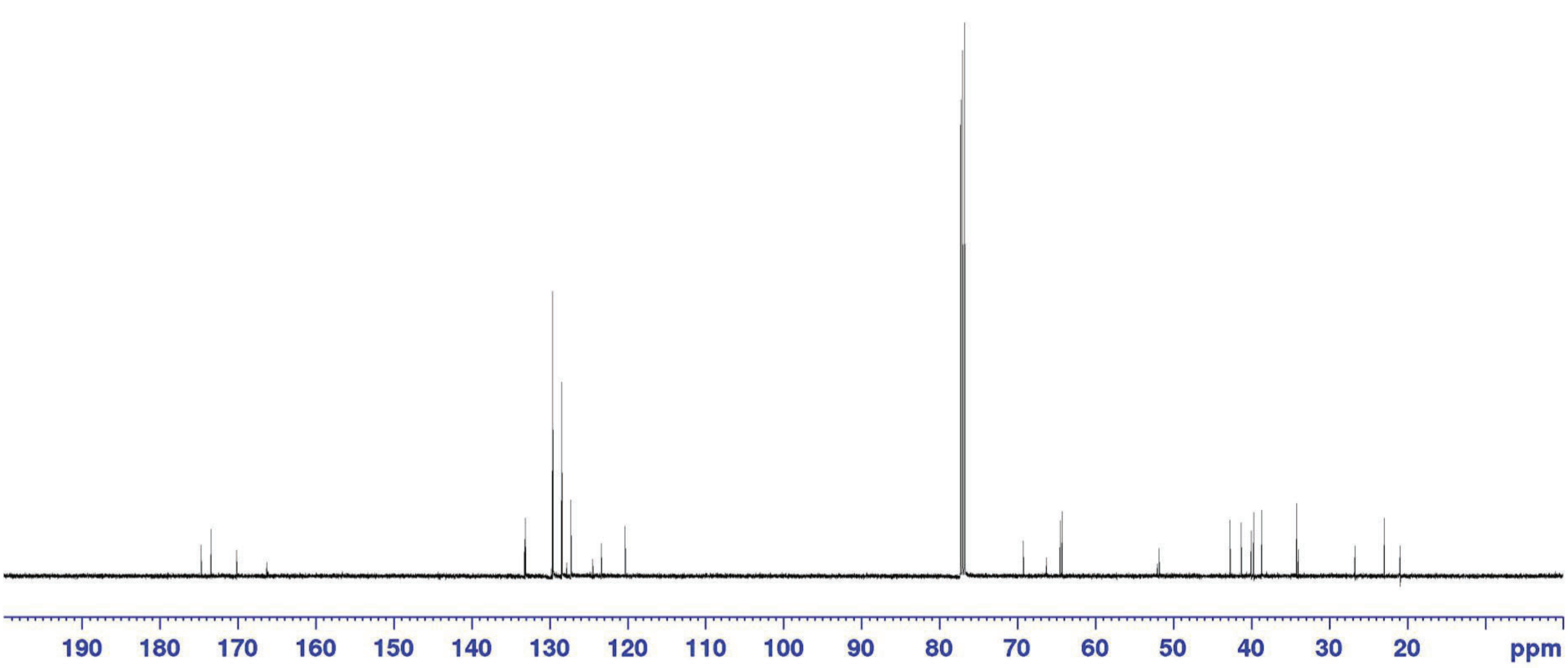

^{1}H NMR data of compound **3c**
500 MHz

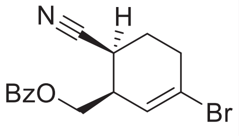

3c *endo*

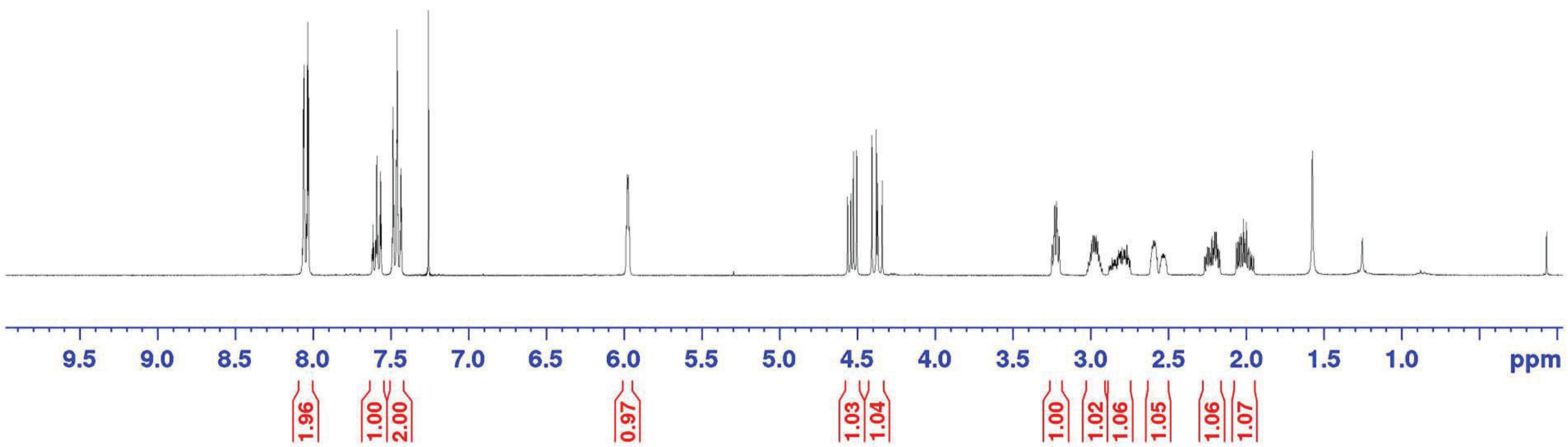

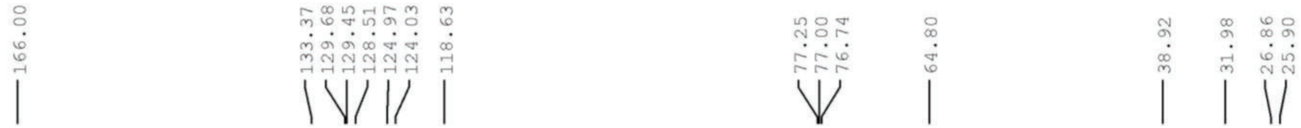

^{13}C NMR data of compound **3c**
500 MHz

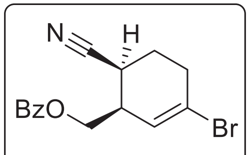

3c *endo*

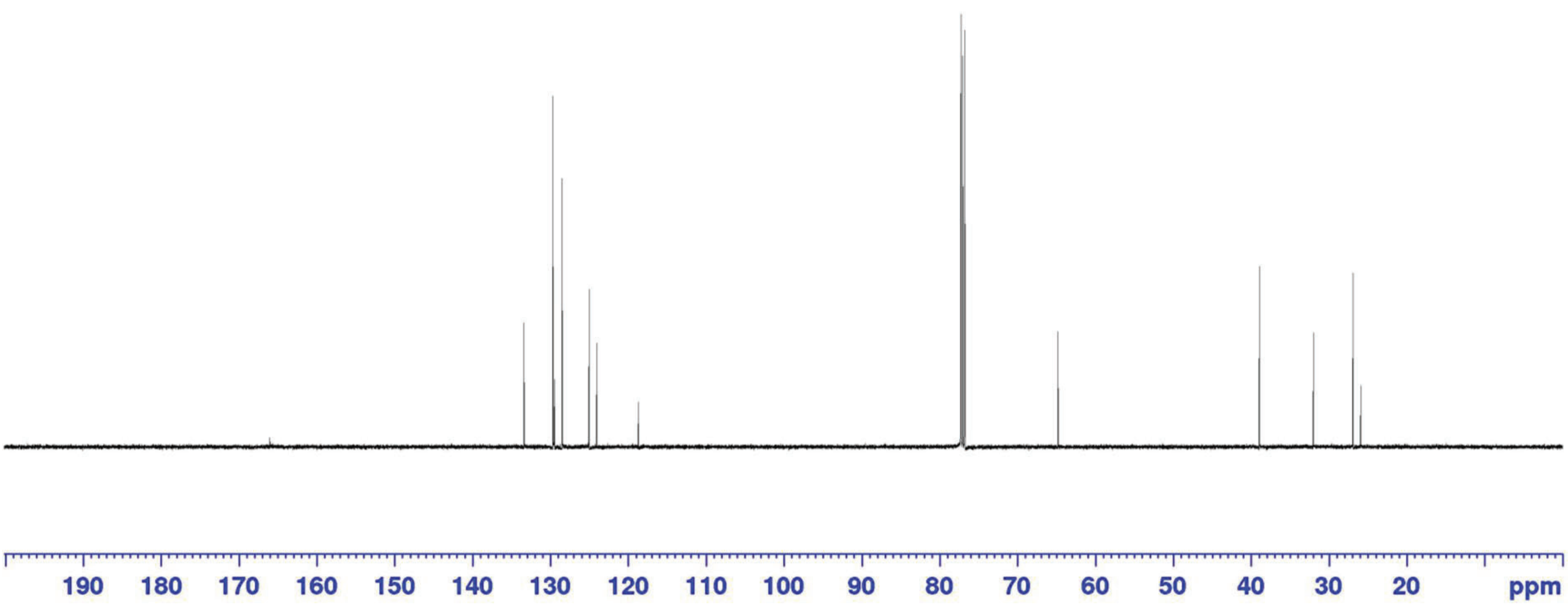

^{1}H NMR data of compound **3c’**
500 MHz

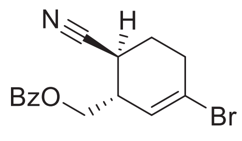

3c' *exo*

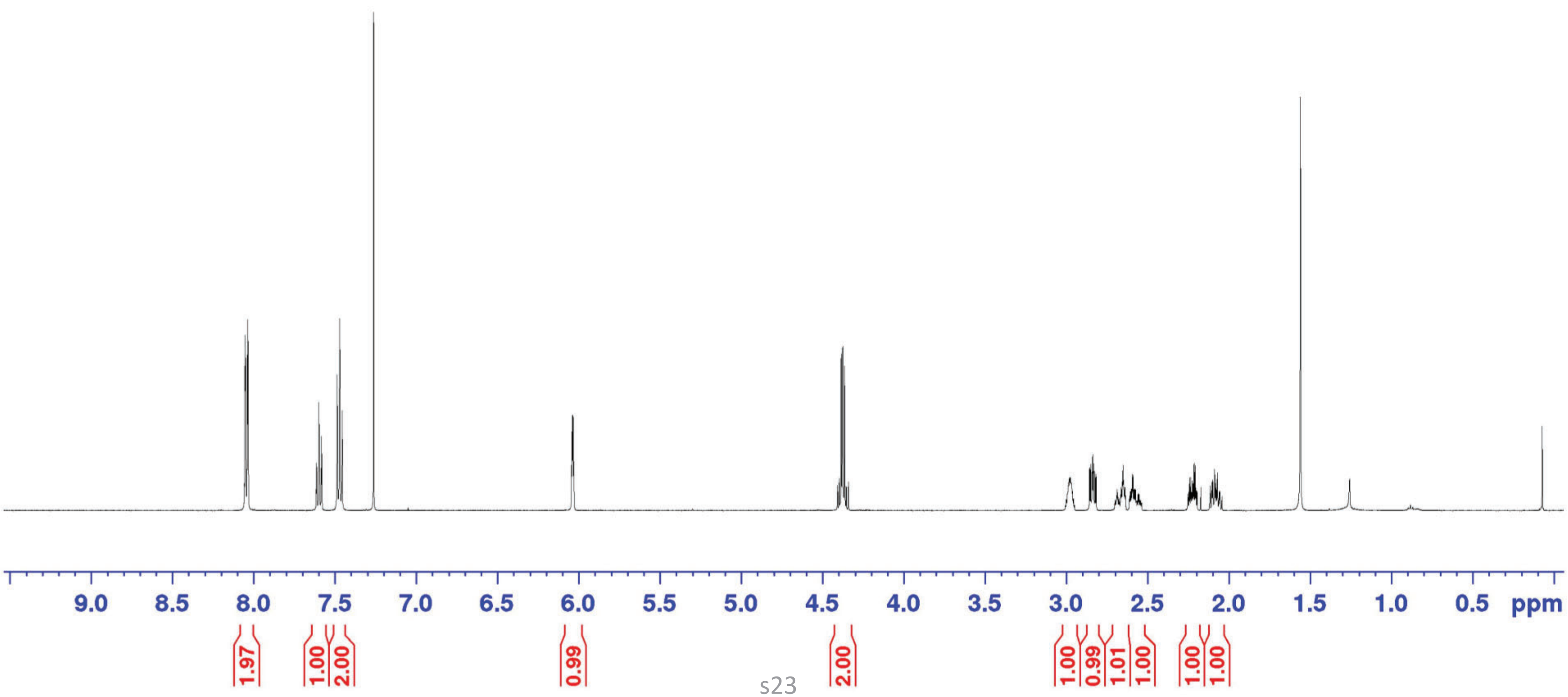

^{13}C NMR data of compound **3c'**
500 MHz

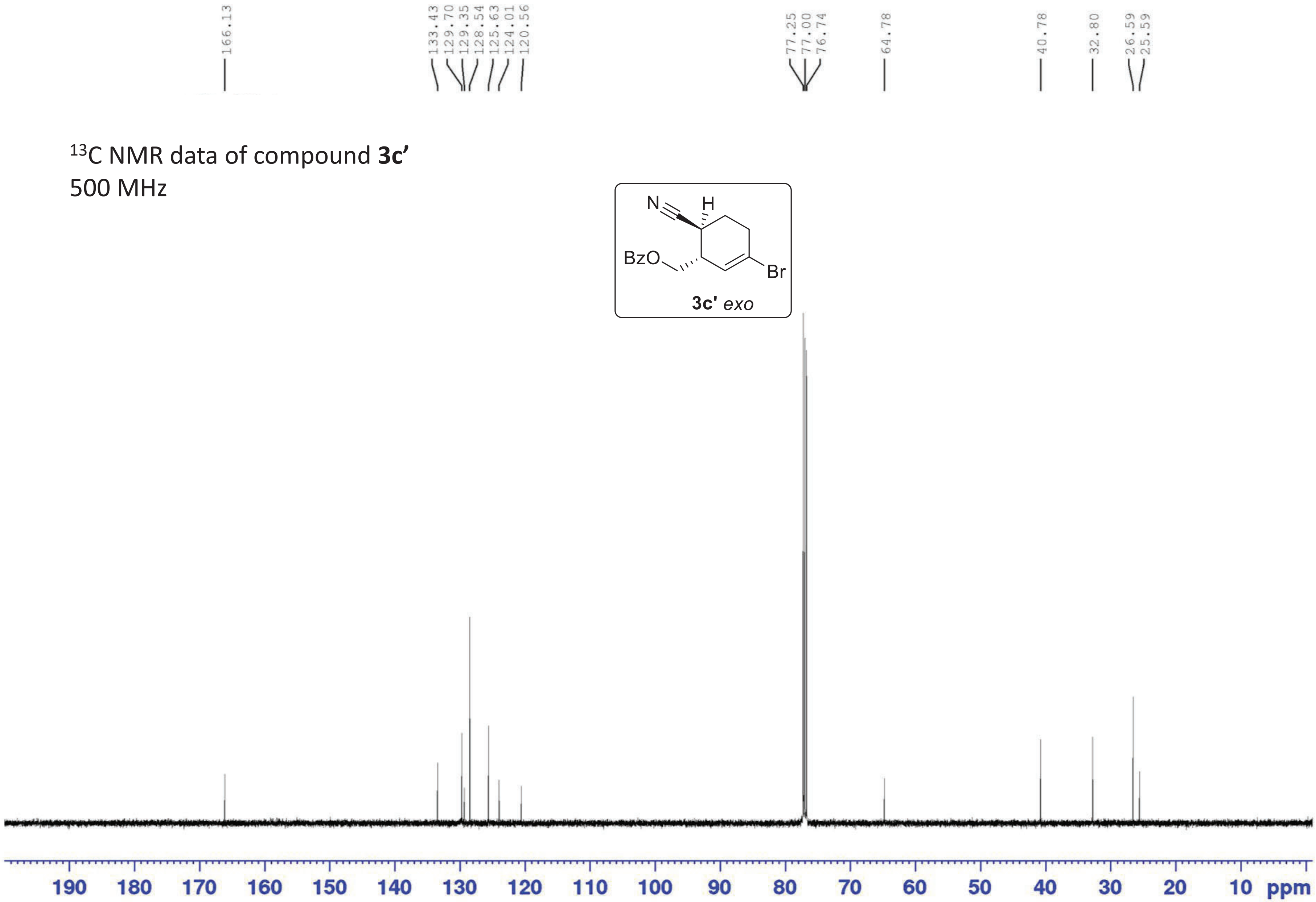

^{1}H NMR data of compound **3d**
500 MHz

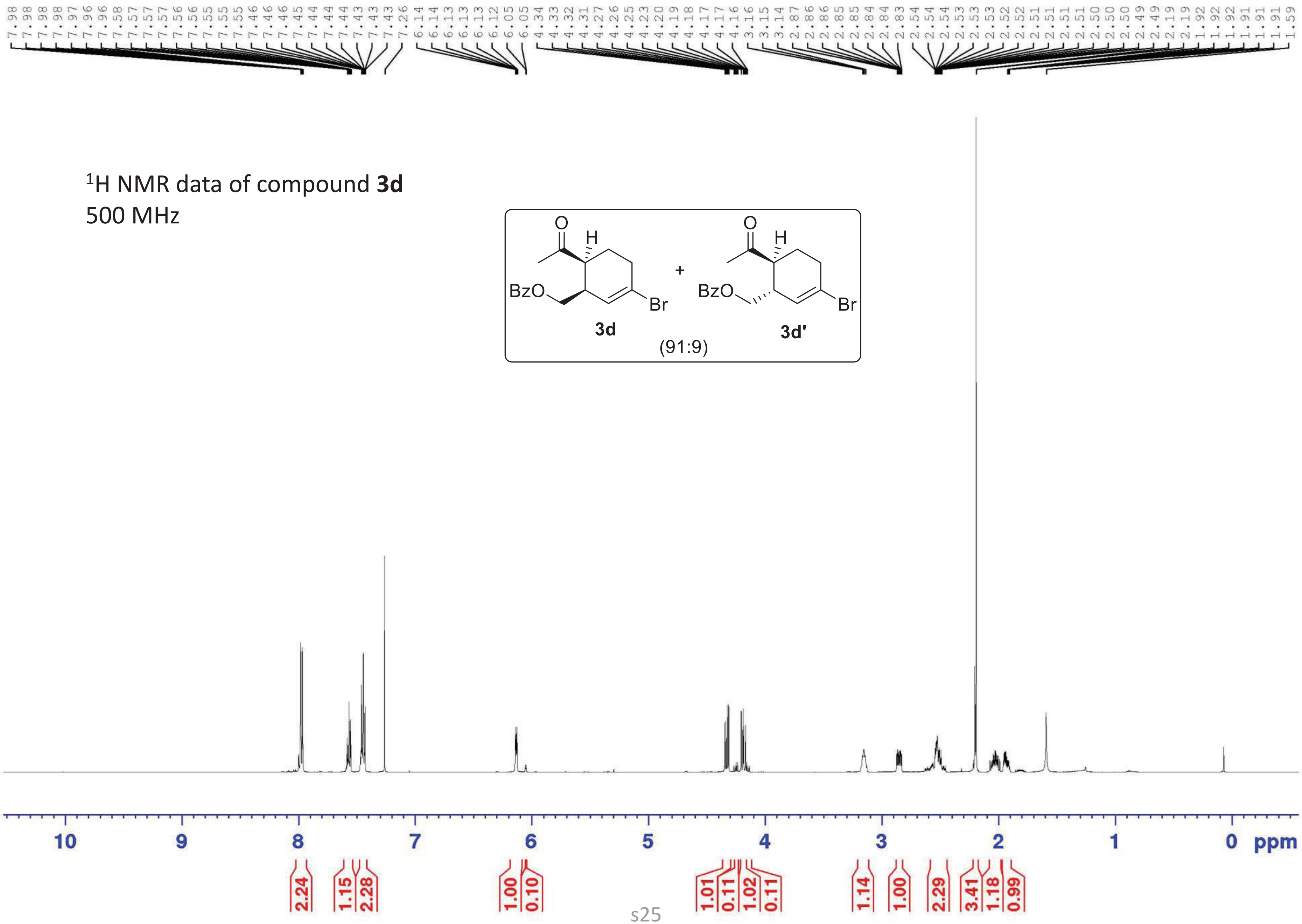

^{13}C NMR data of compound **3d**
500 MHz

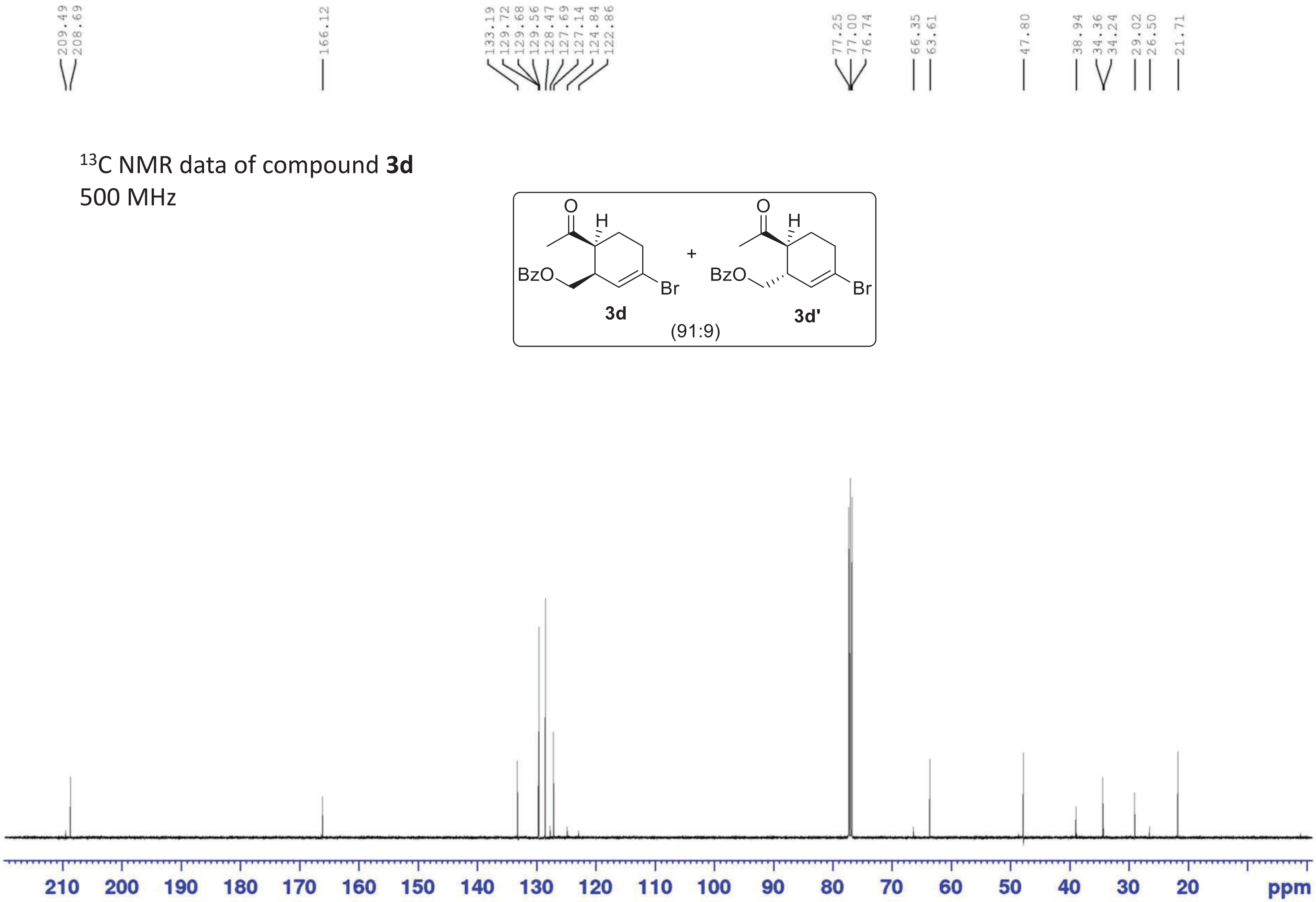

^{1}H NMR data of compound **3e**
500 MHz

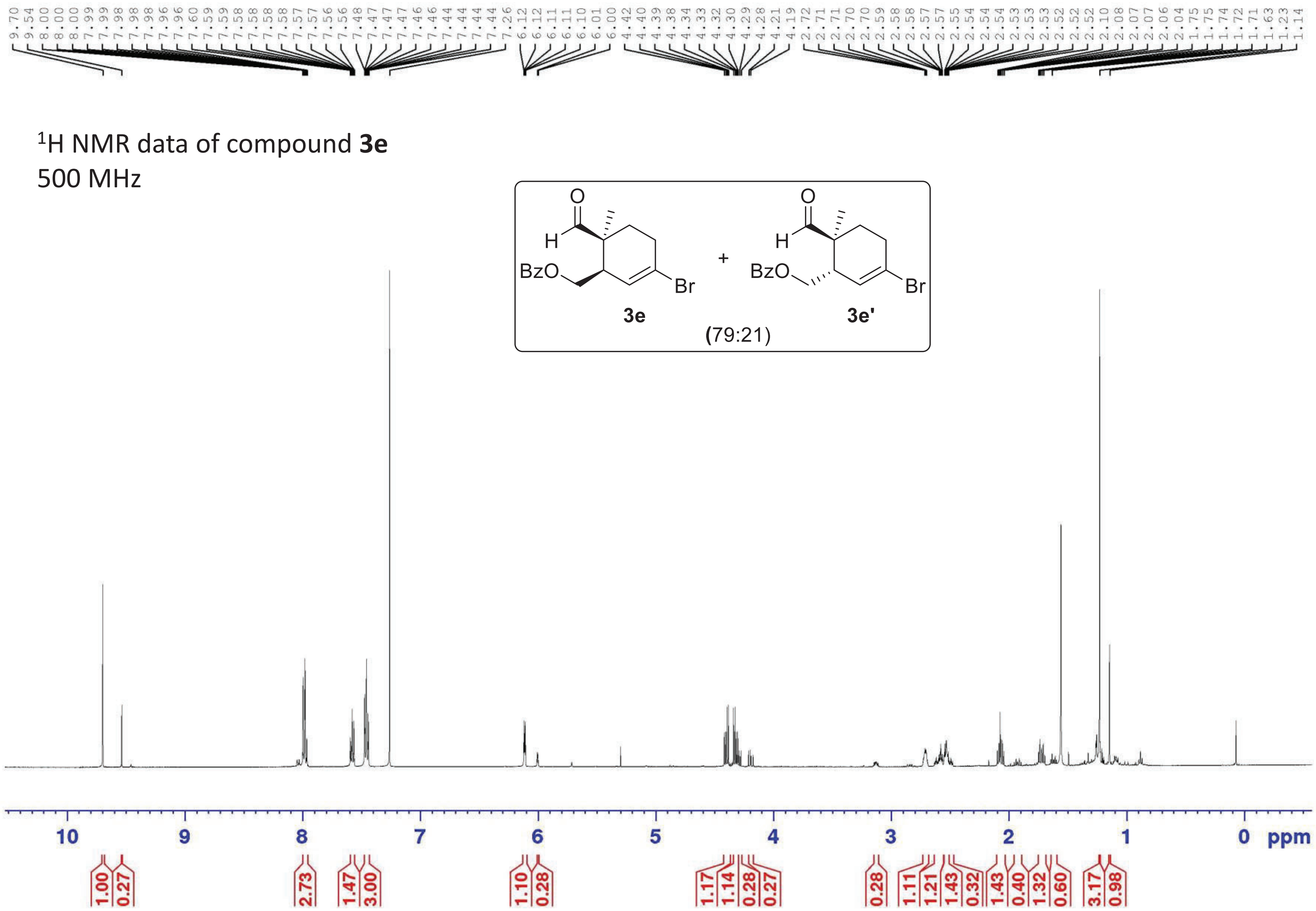

^{13}C NMR data of compound **3e**
500 MHz

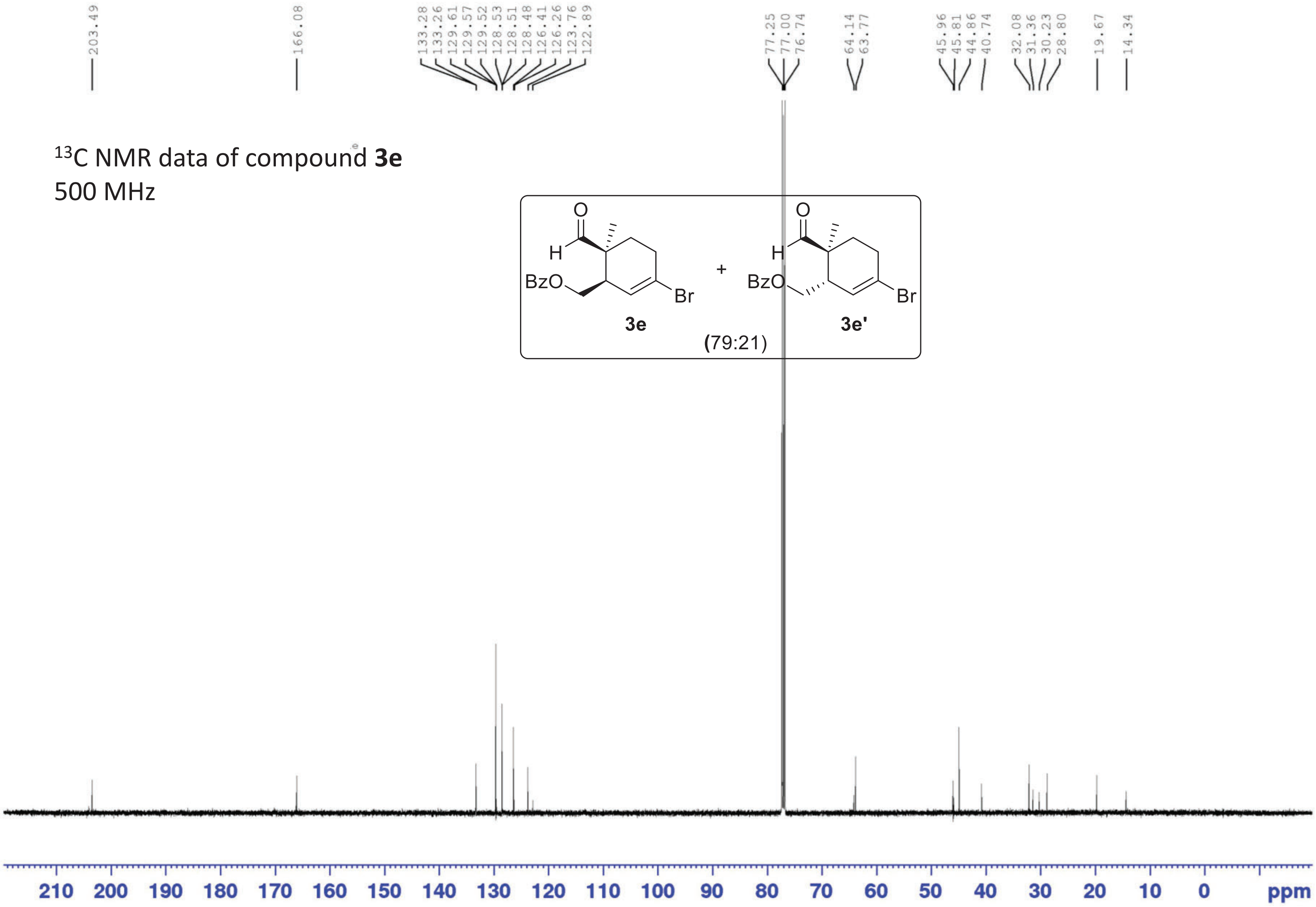

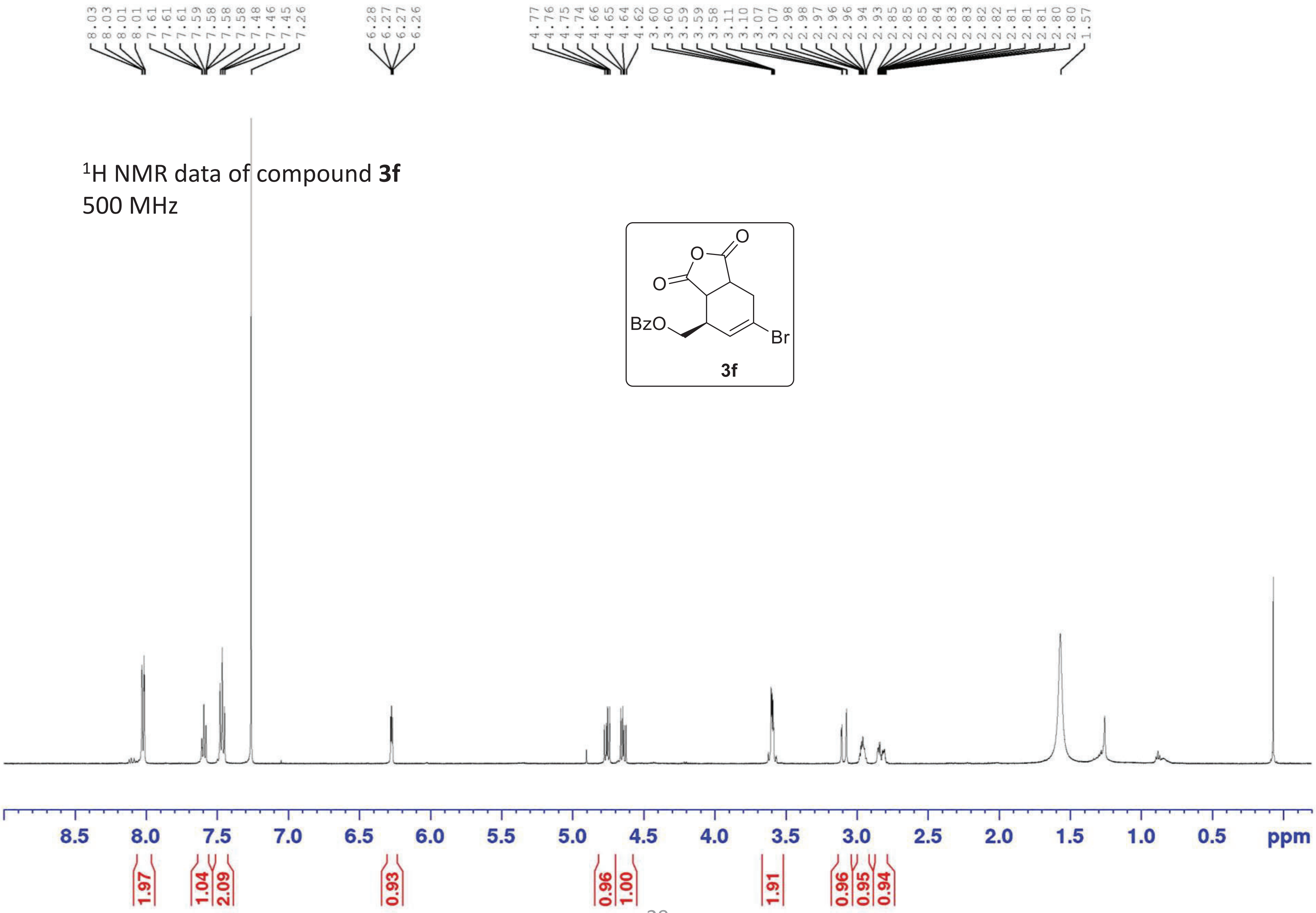

8.03 8.03 8.01 8.01 7.61 7.61 7.61 7.59 7.58 7.58 7.58 7.48 7.46 7.45 7.26
6.28 6.27 6.27 6.26
4.77 4.76 4.75 4.74 4.66 4.65 4.64 4.62 3.60 3.60 3.59 3.59 3.58 3.11 3.10 3.07 3.07 2.98 2.98 2.97 2.96 2.96 2.94 2.93 2.85 2.85 2.84 2.83 2.83 2.82 2.82 2.81 2.81 2.81 2.80 2.80 1.57
^{1}H NMR data of compound **3f**
500 MHz
O
O
O
BzO
Br
3f
8.5 8.0 7.5 7.0 6.5 6.0 5.5 5.0 4.5 4.0 3.5 3.0 2.5 2.0 1.5 1.0 0.5 ppm
1.97 1.04 2.09 0.93 0.96 1.00 1.91 0.96 0.95 0.94

^{13}C NMR data of compound **3f**
500 MHz

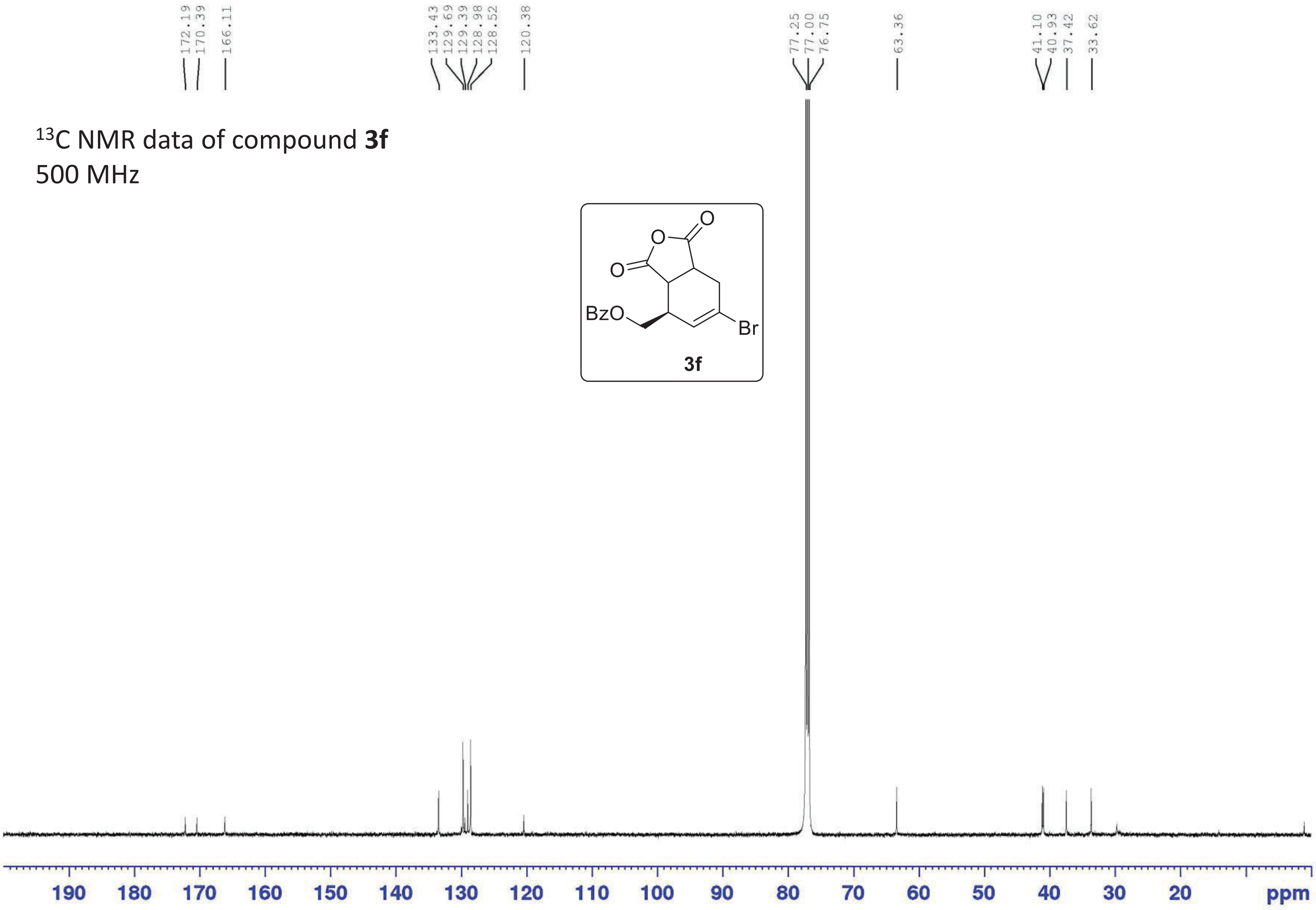

^{1}H NMR data of compound **3g**
500 MHz

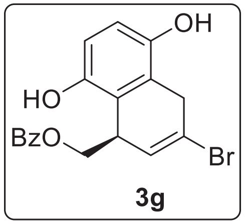

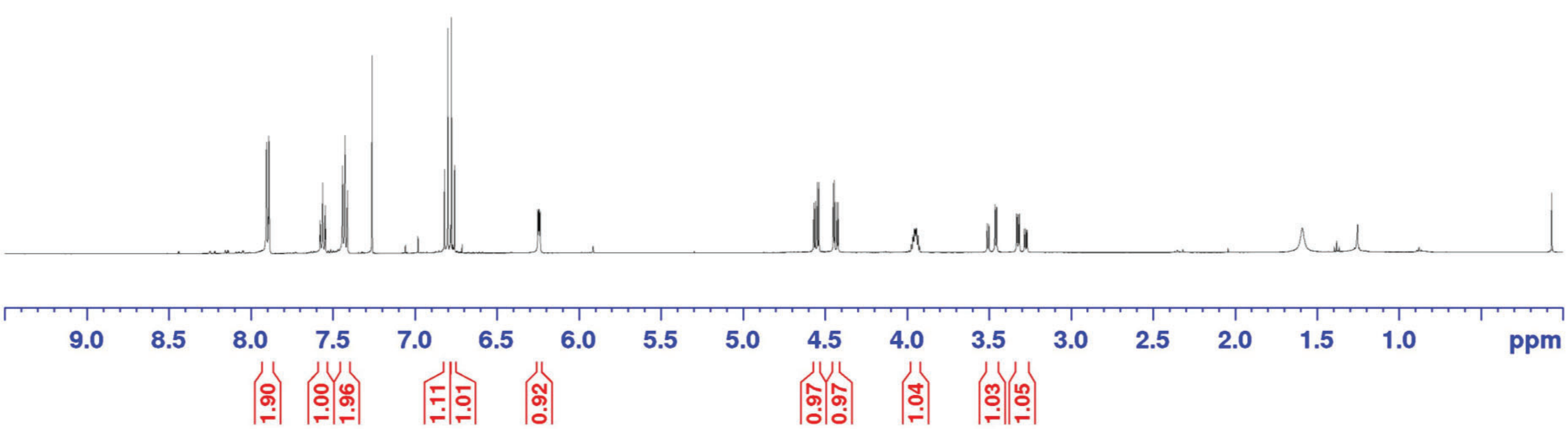

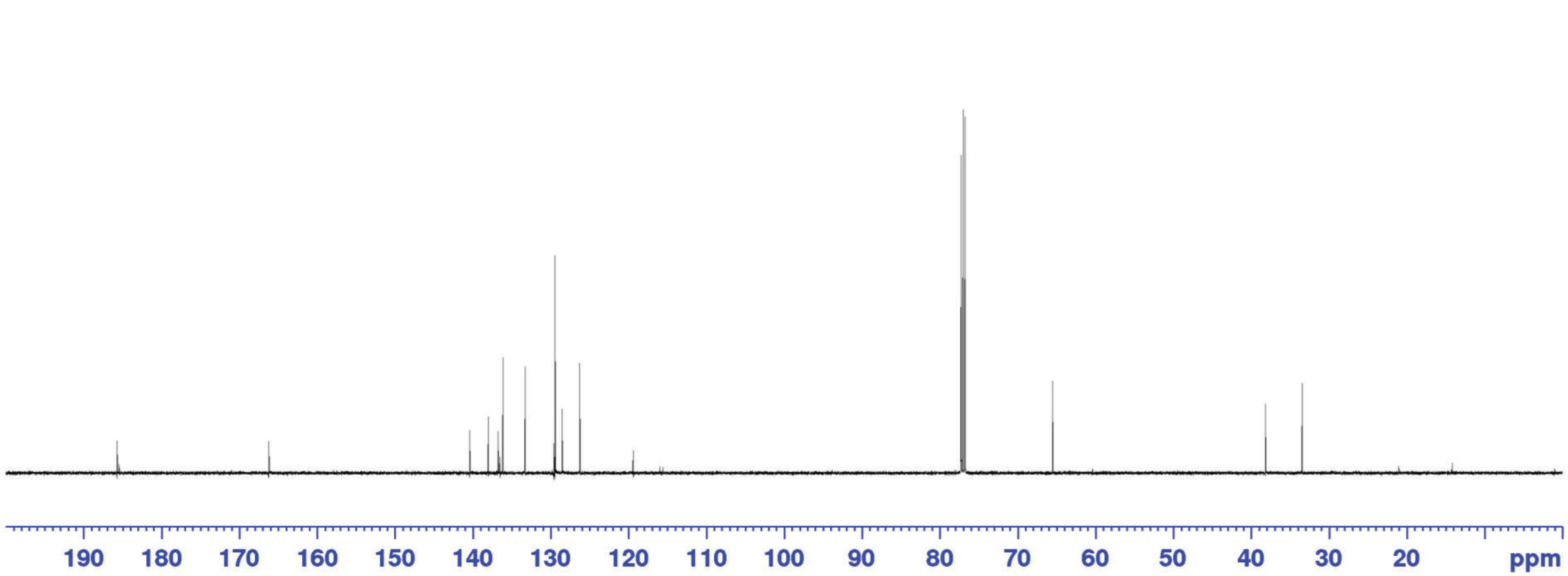

^{13}C NMR data of compound **3g**
500 MHz

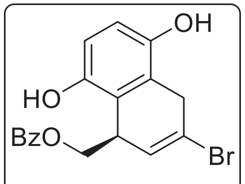

3g

^{1}H NMR data of compound **6a**
500 MHz

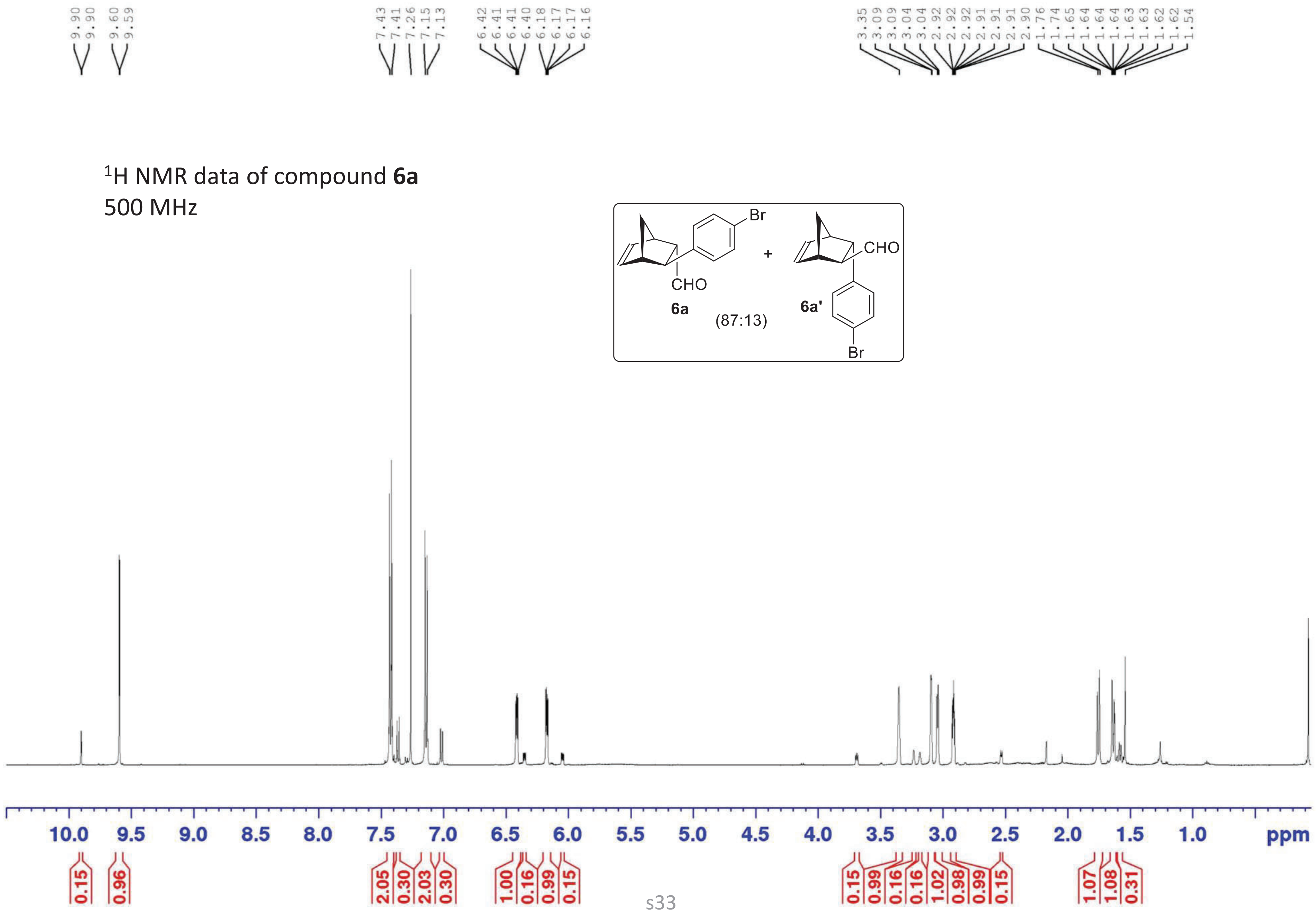

^{13}C NMR data of compound **6a**
500 MHz

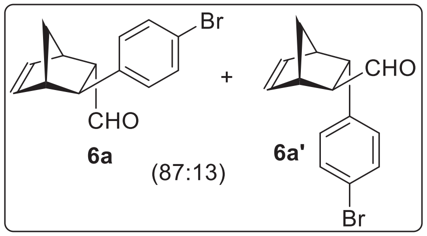

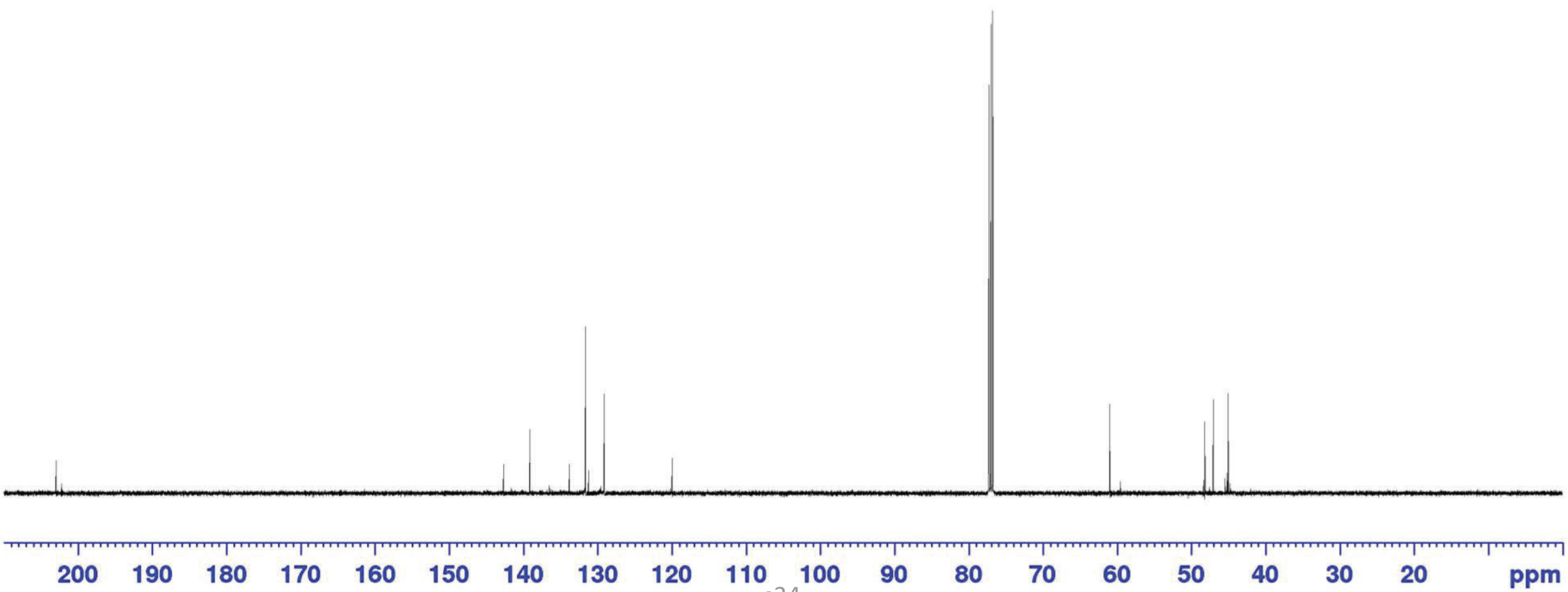

^{1}H NMR data of compound **6b**
500 MHz

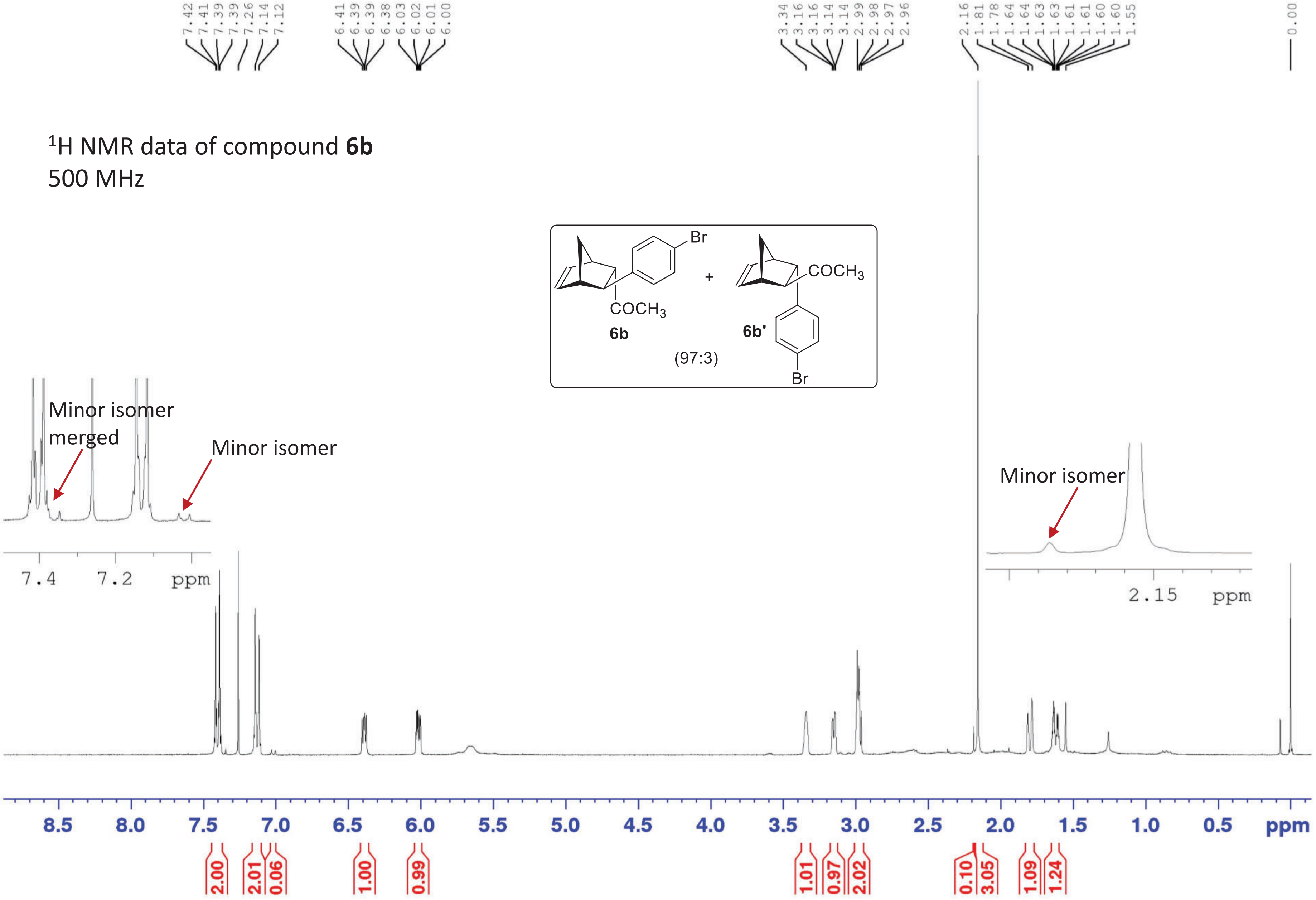

^{13}C NMR data of compound **6b**
500 MHz

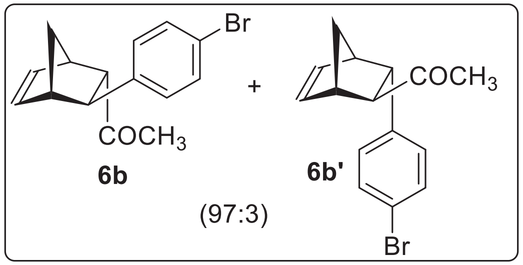

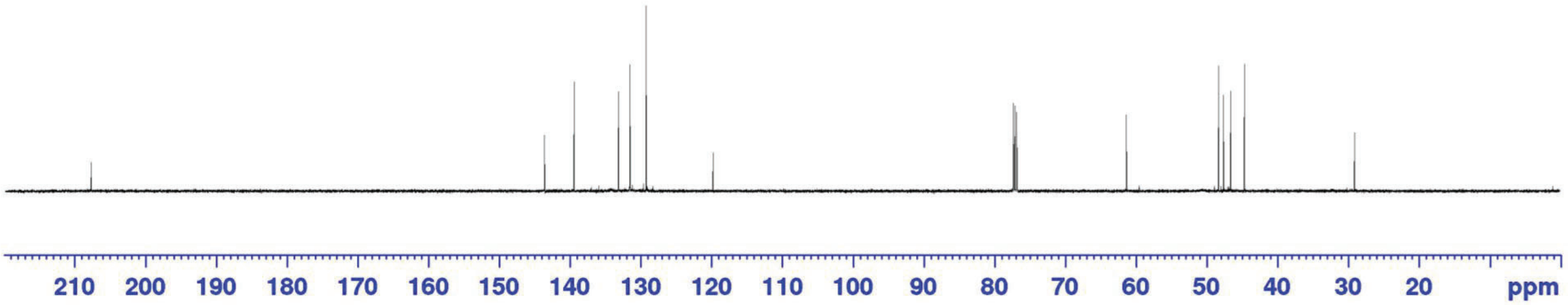

^{1}H NMR data of compound **6c**
500 MHz

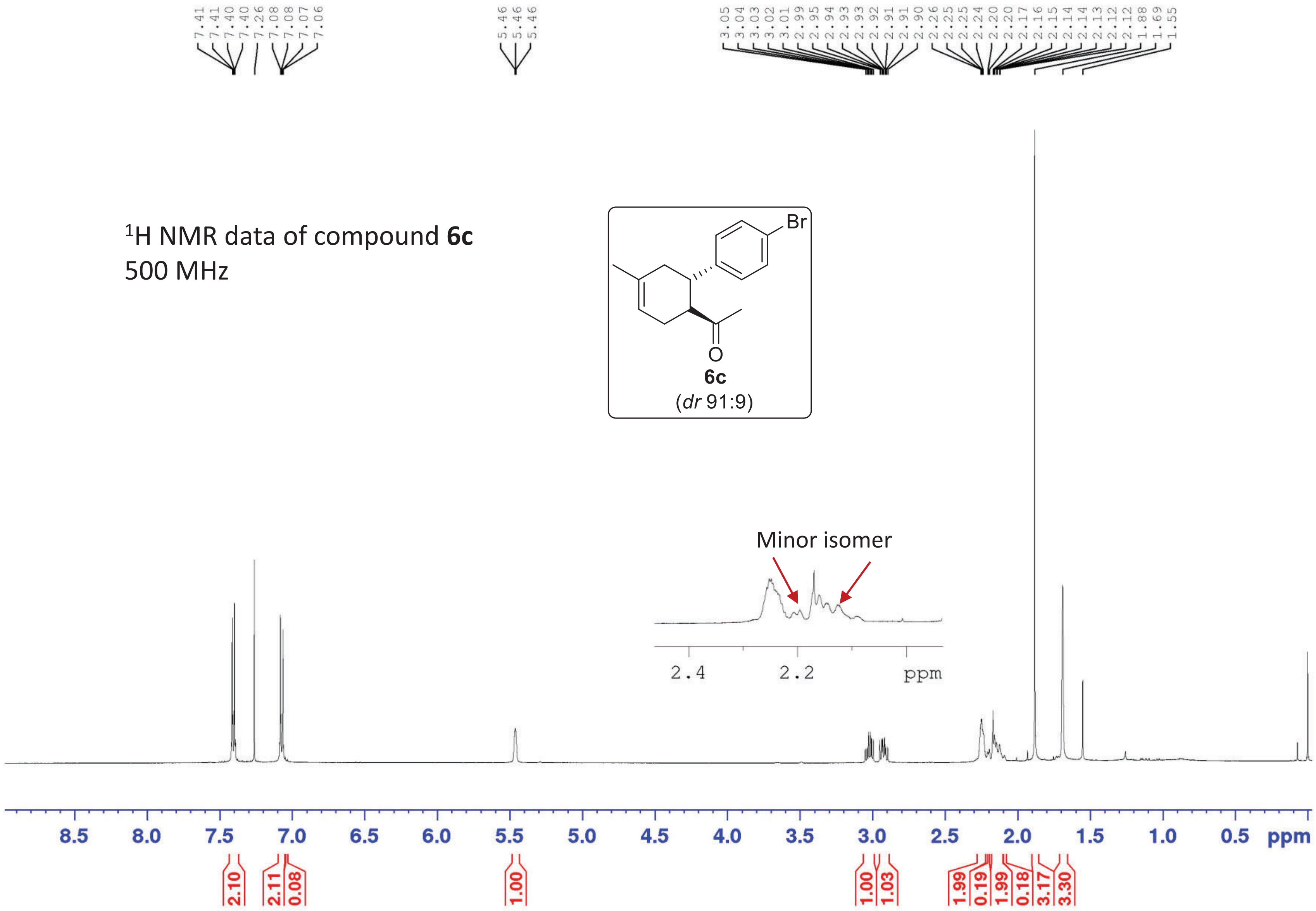

^{13}C NMR data of compound **6c**
500 MHz

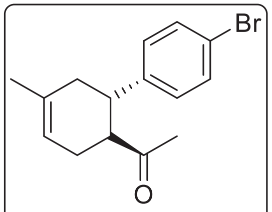

6c
(*dr* 91:9)

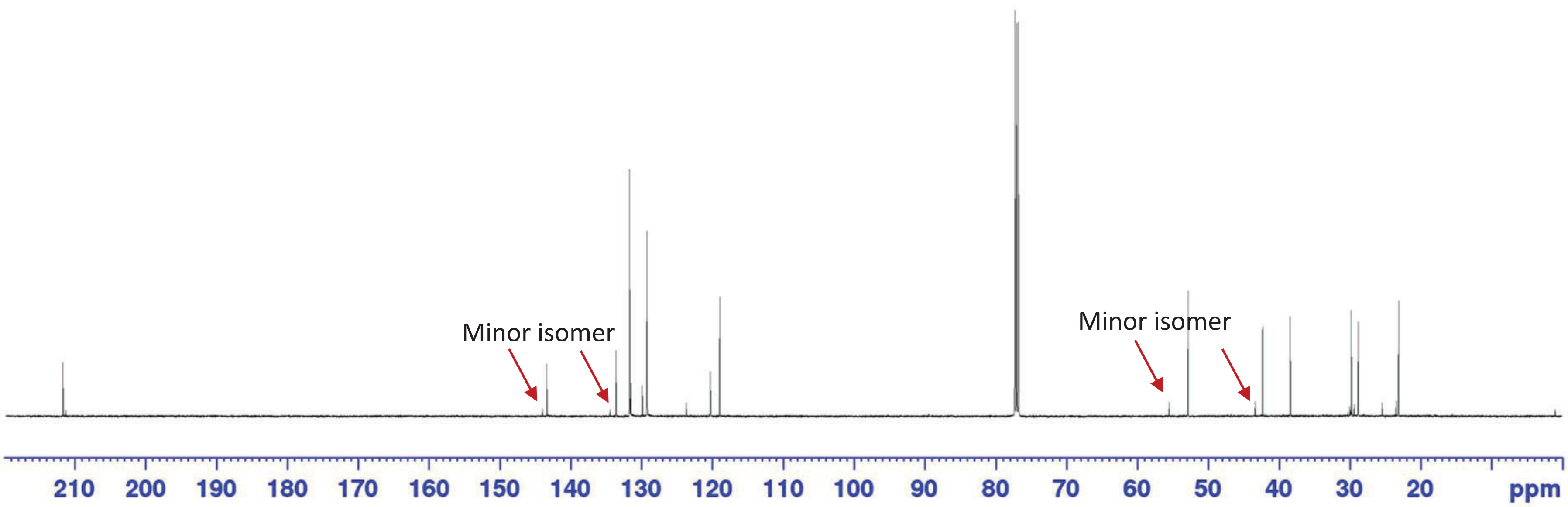

^{1}H NMR data of compound **6d**
500 MHz

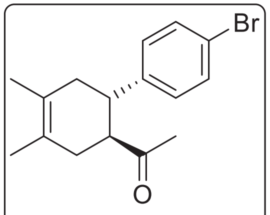

6d
(*dr* >99:1)

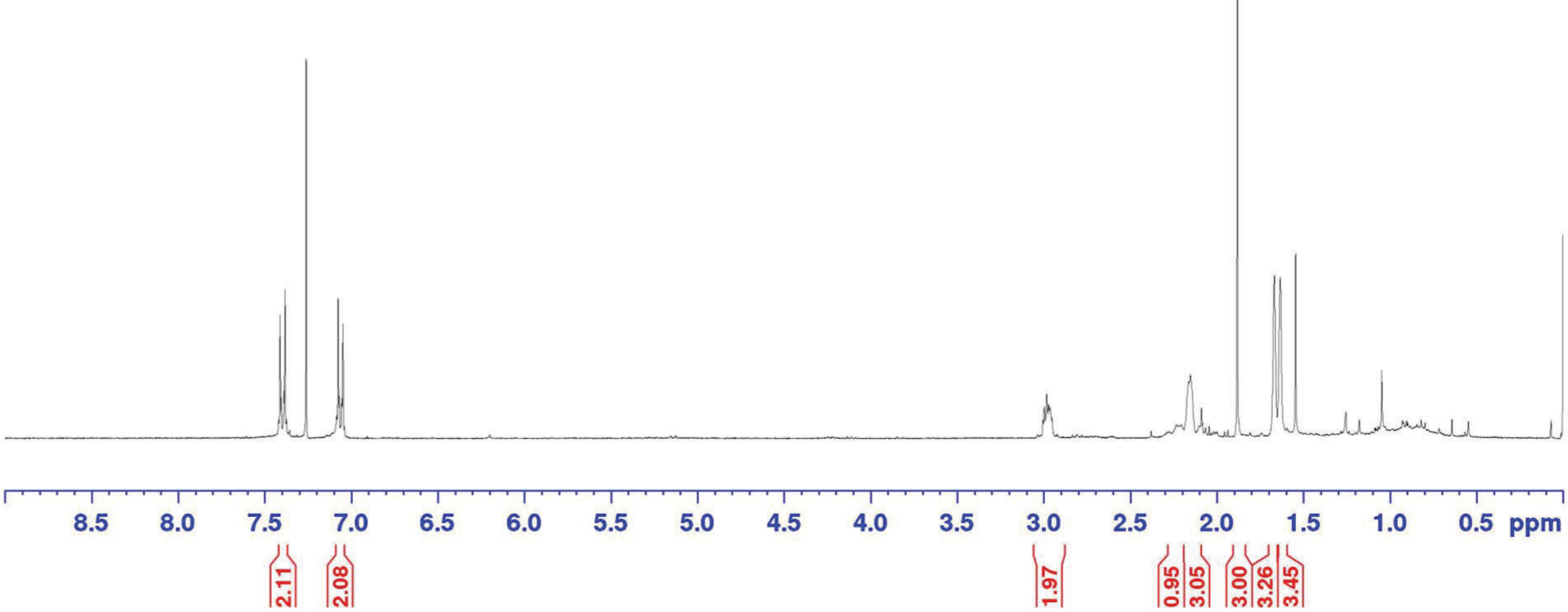

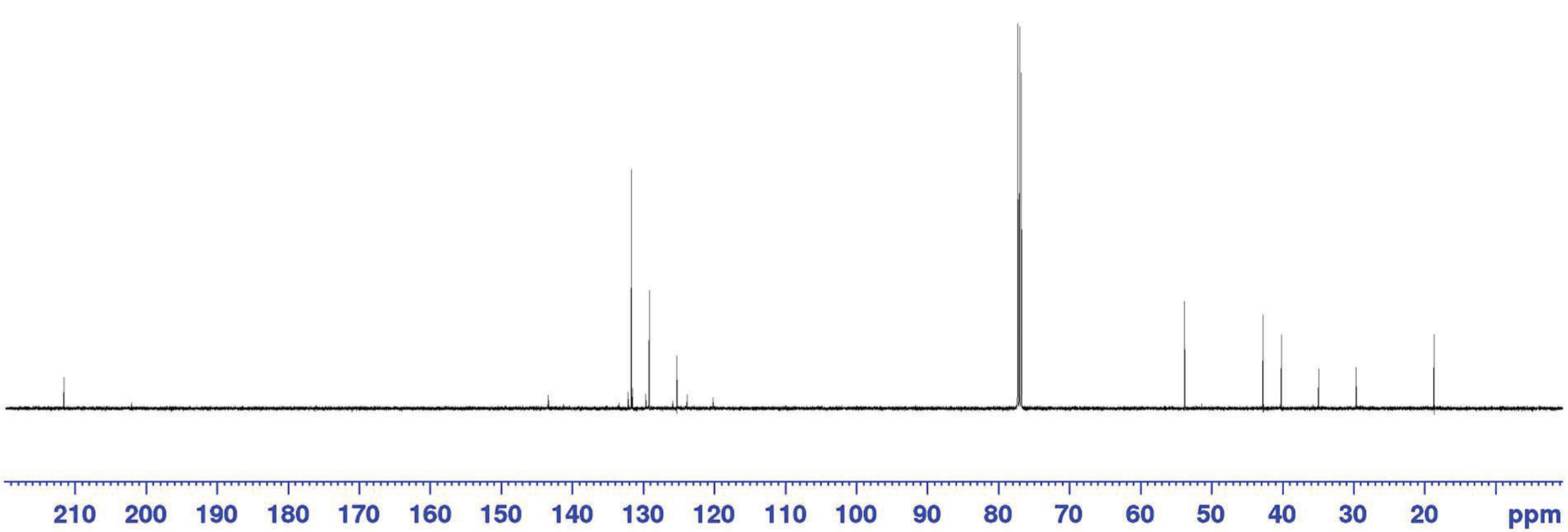

^{13}C NMR data of compound **6d**
500 MHz

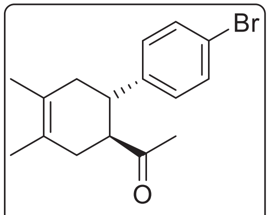

6d
(*dr* >99:1)

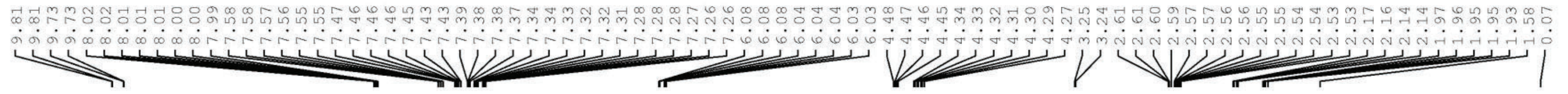

^{1}H NMR data of compound **7a**
500 MHz

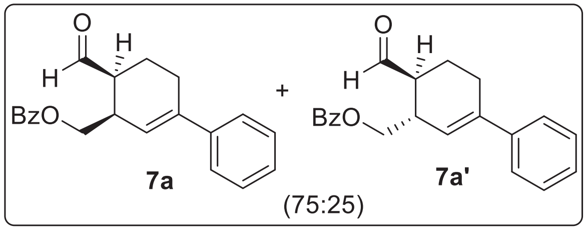

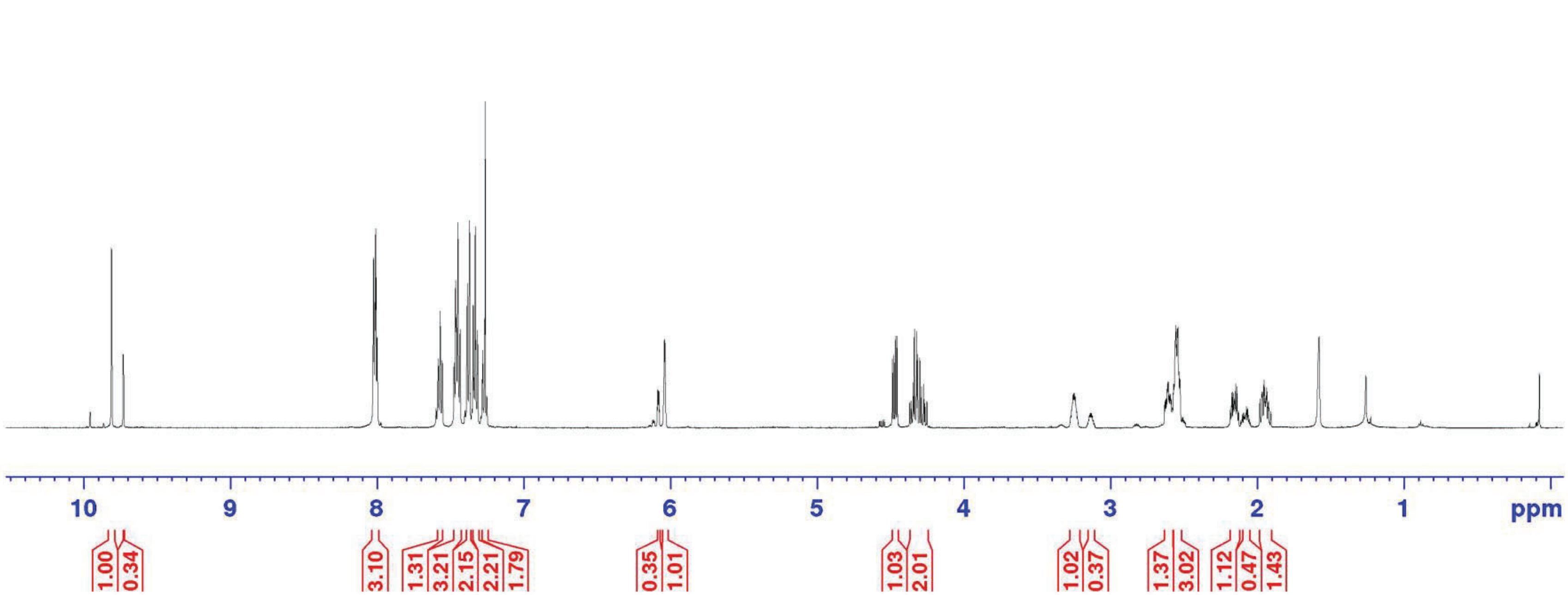

^{13}C NMR data of compound **7a**
500 MHz

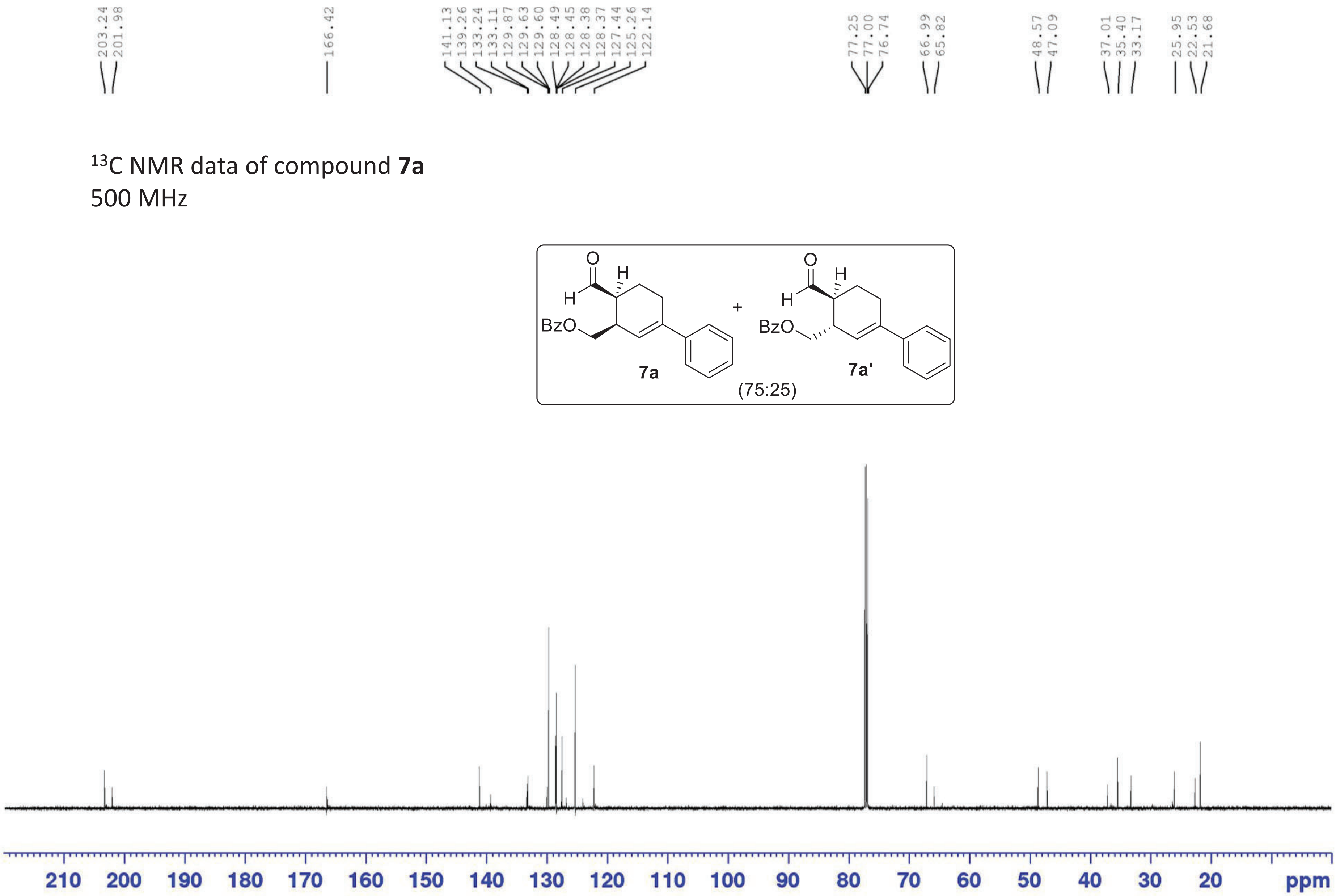

^{1}H NMR data of compound **7b**
500 MHz

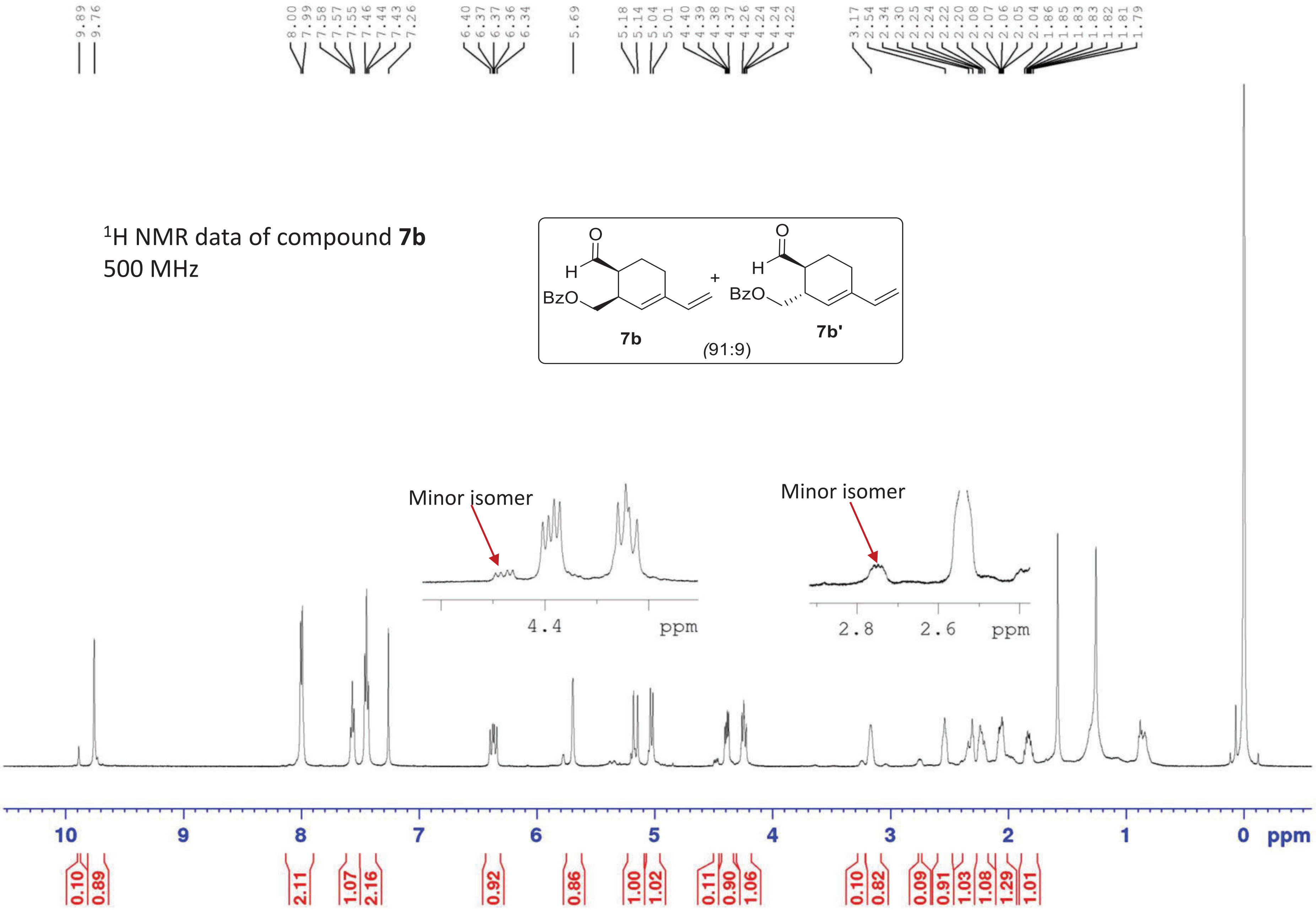

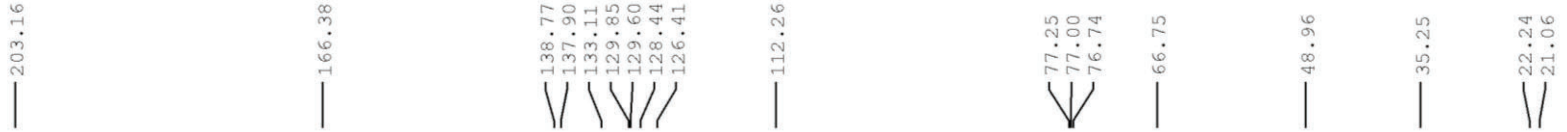

^{13}C NMR data of compound **7b**
500 MHz

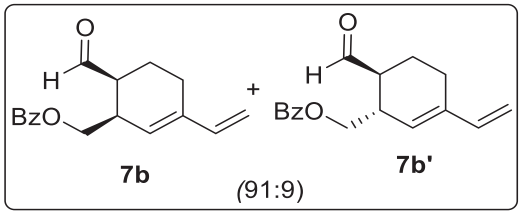

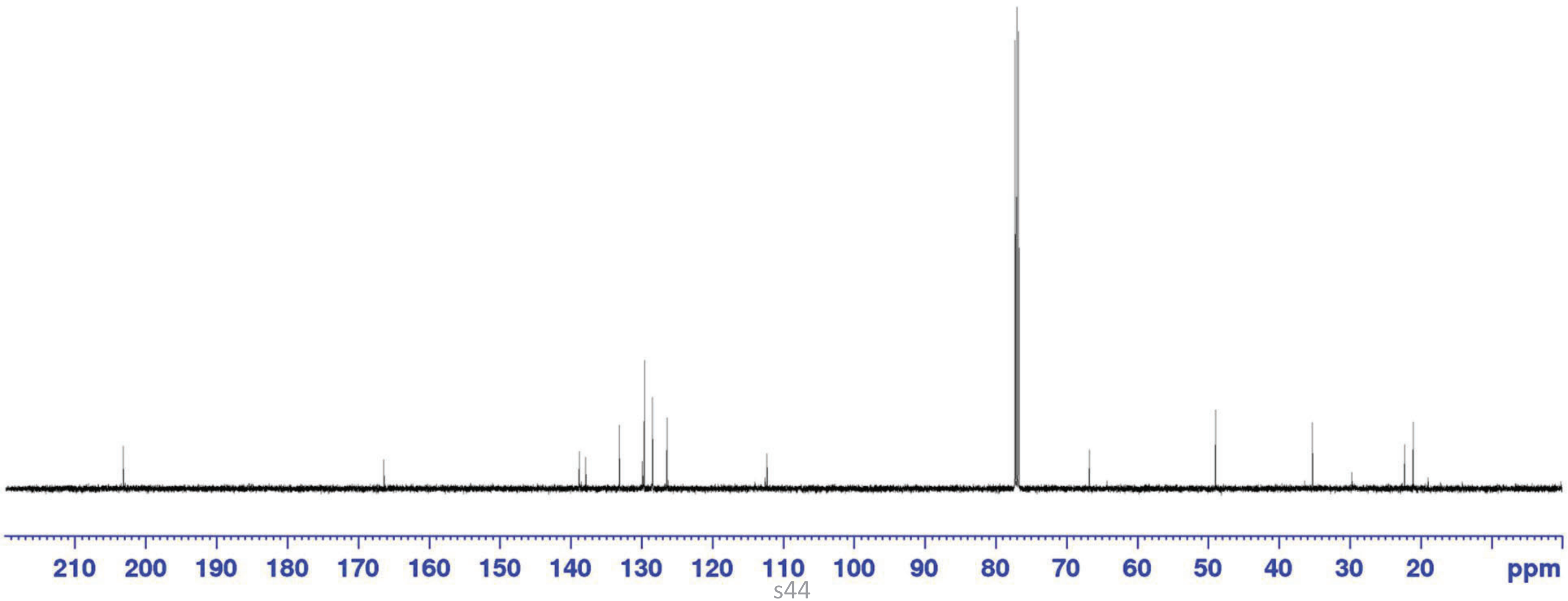

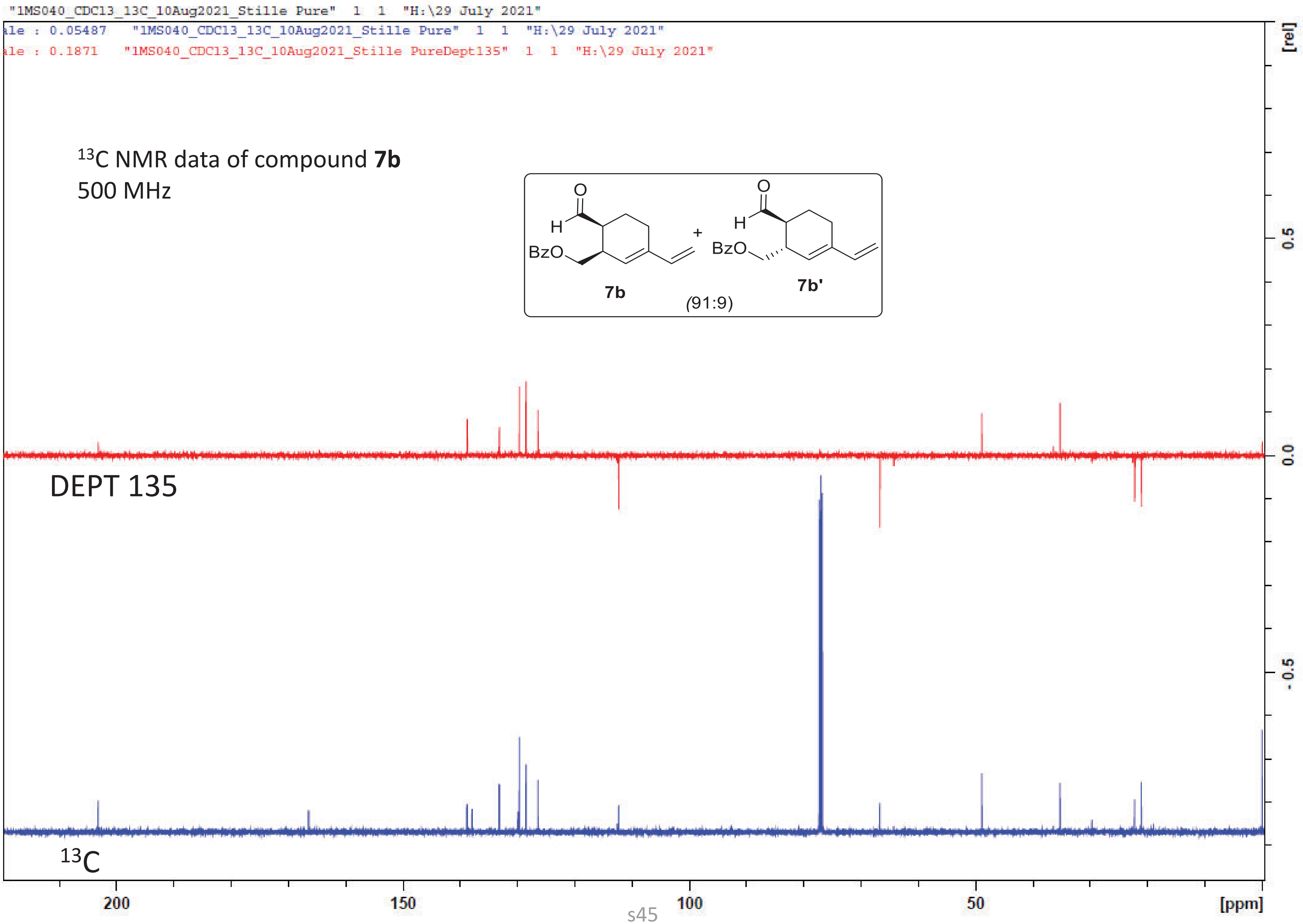
"1MS040_CDCl3_13C_10Aug2021_Stille Pure" 1 1 "H:\29 July 2021"
ale : 0.05487 "1MS040_CDCl3_13C_10Aug2021_Stille Pure" 1 1 "H:\29 July 2021"
ale : 0.1871 "1MS040_CDCl3_13C_10Aug2021_Stille PureDept135" 1 1 "H:\29 July 2021"
13C NMR data of compound 7b
500 MHz
O
H
BzO
7b
+
O
H
BzO
7b'
(91:9)
DEPT 135
13C
[rel]
0.5
0.0
-0.5
200
150
100
50
[ppm]

^{1}H NMR data of compound **7c**
500 MHz

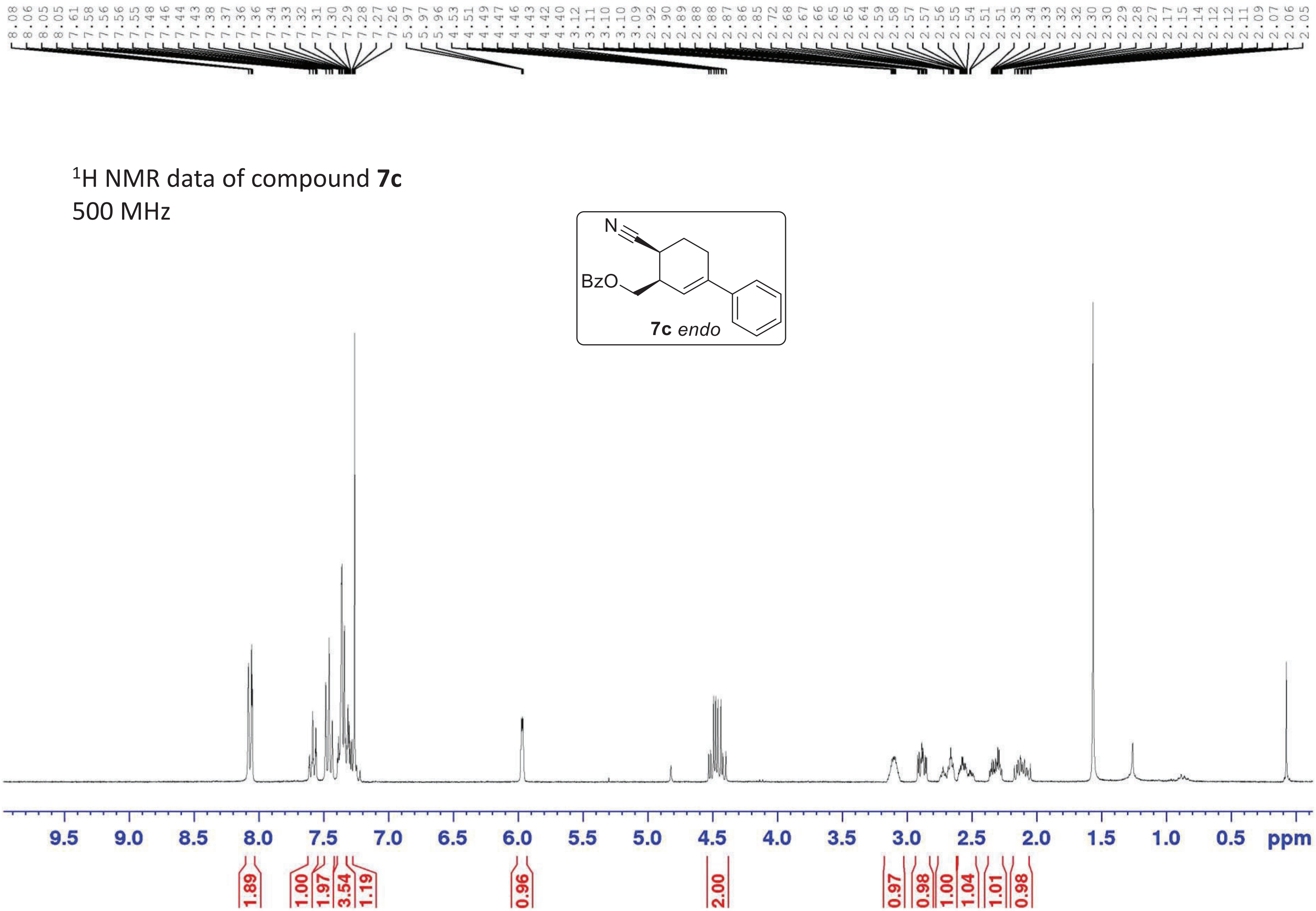

^{13}C NMR data of compound **7c**
500 MHz

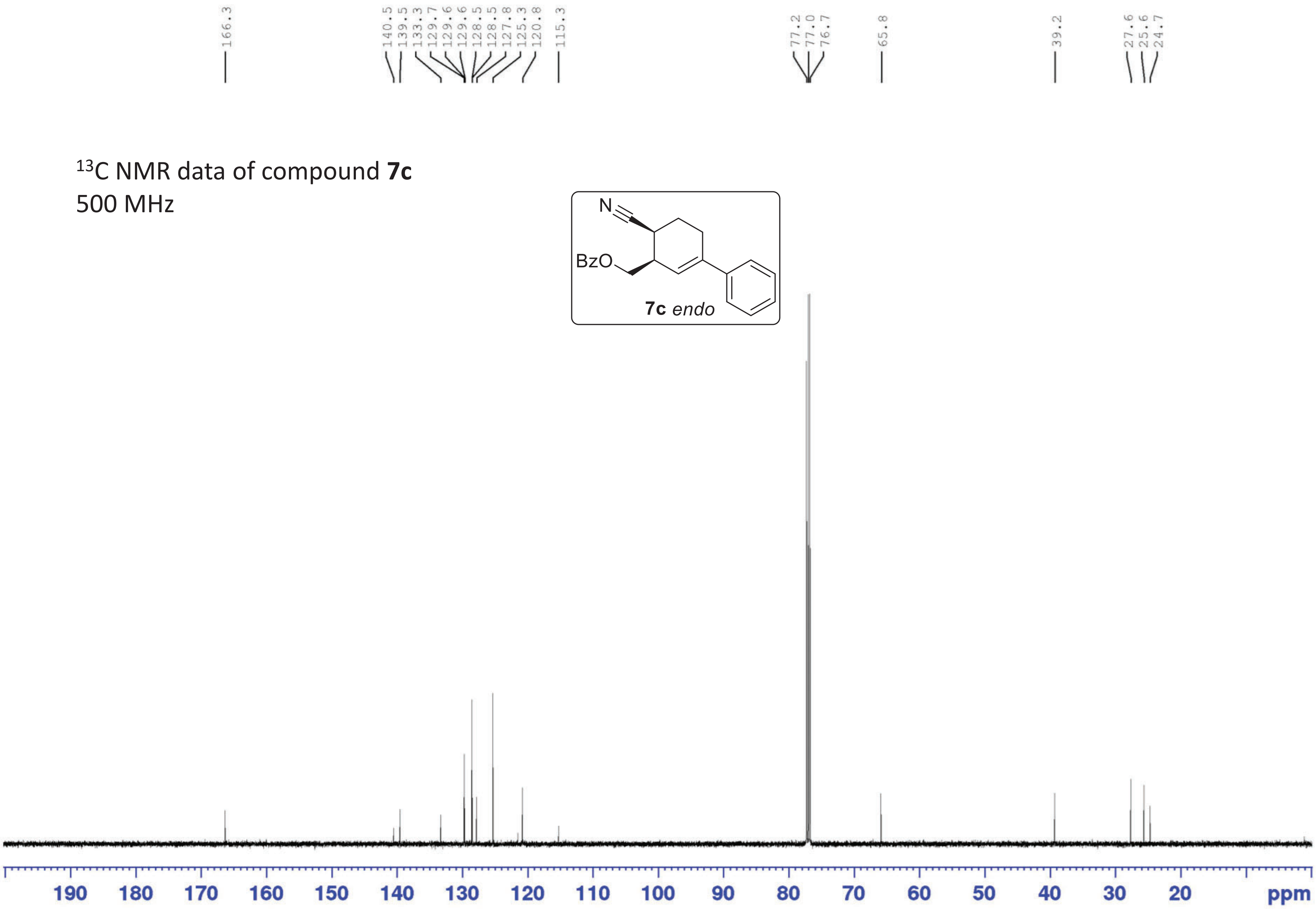

^{1}H NMR data of compound **7c'**
500 MHz

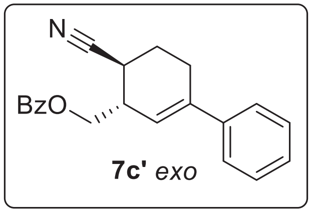

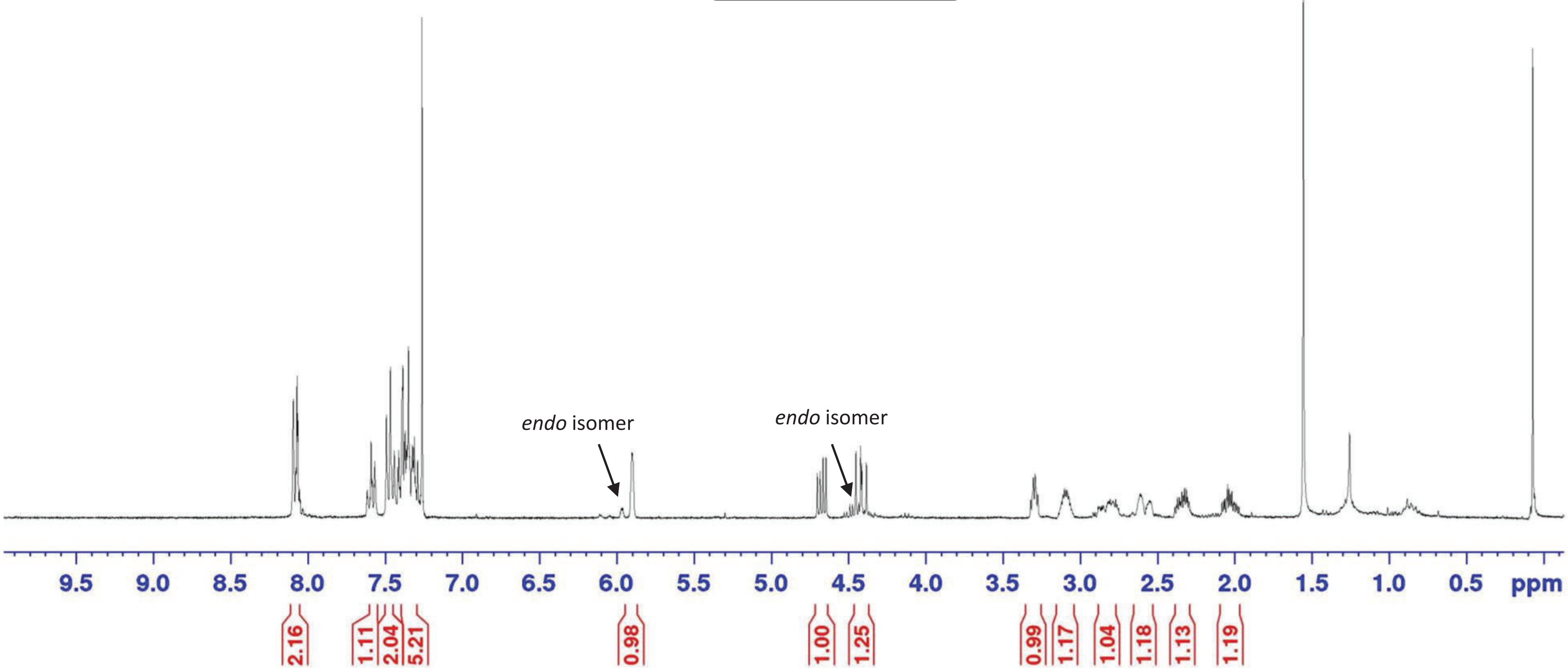

^{13}C NMR data of compound **7c'**
500 MHz

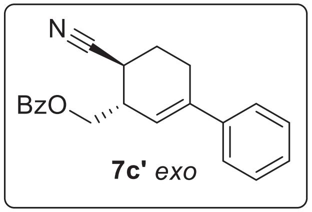

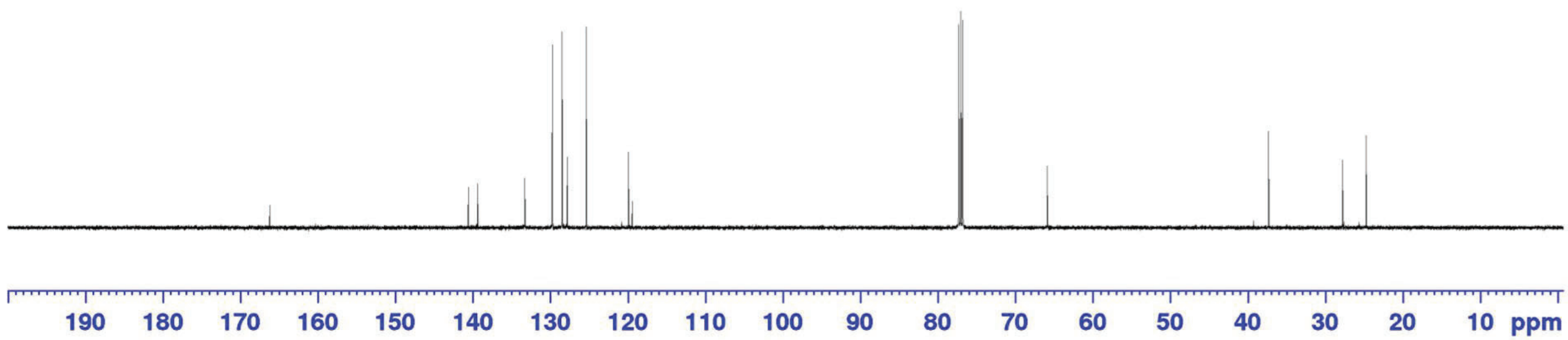

^{1}H NMR data of compound **7d**
500 MHz

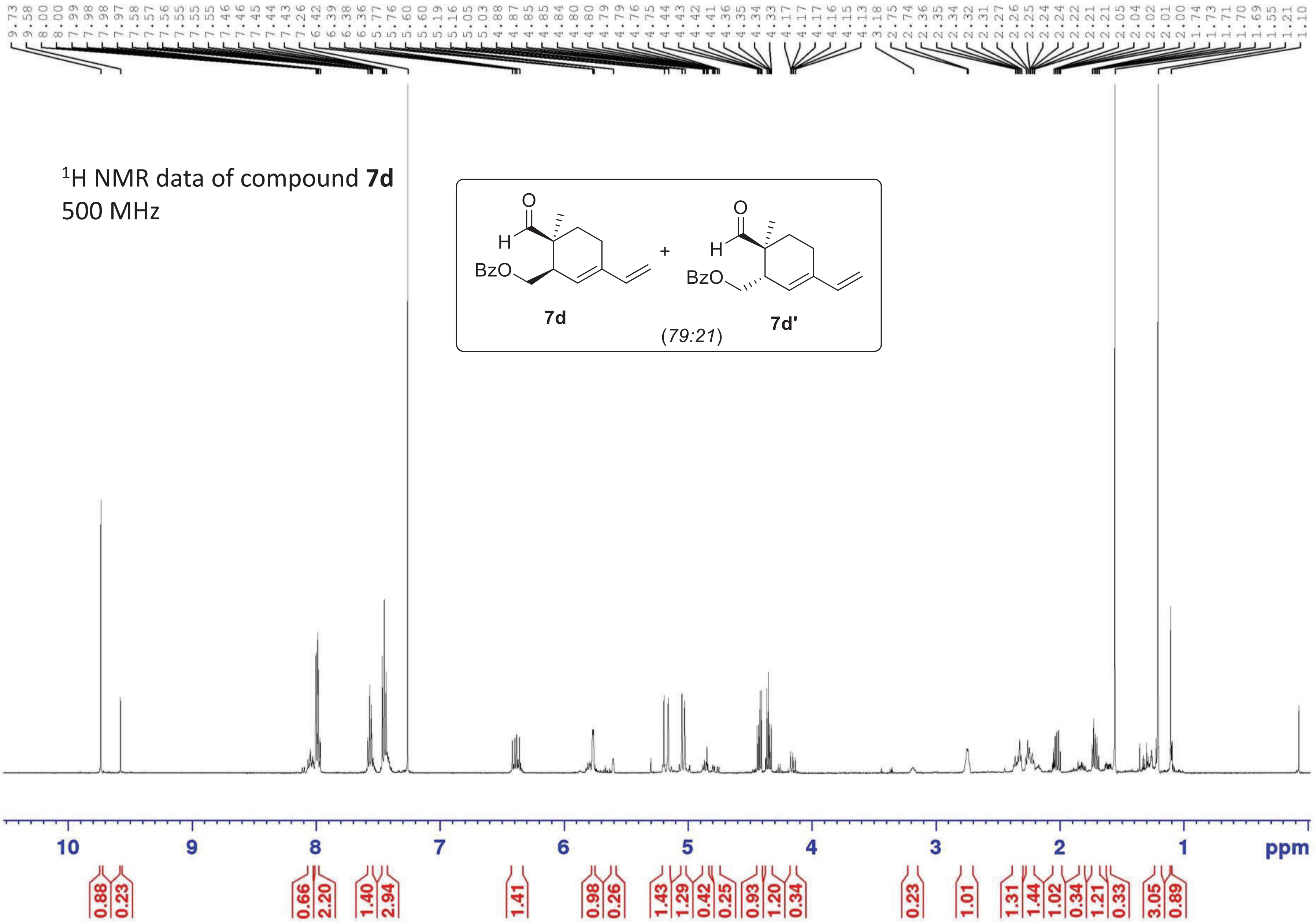

^{13}C NMR data of compound **7d**
500 MHz

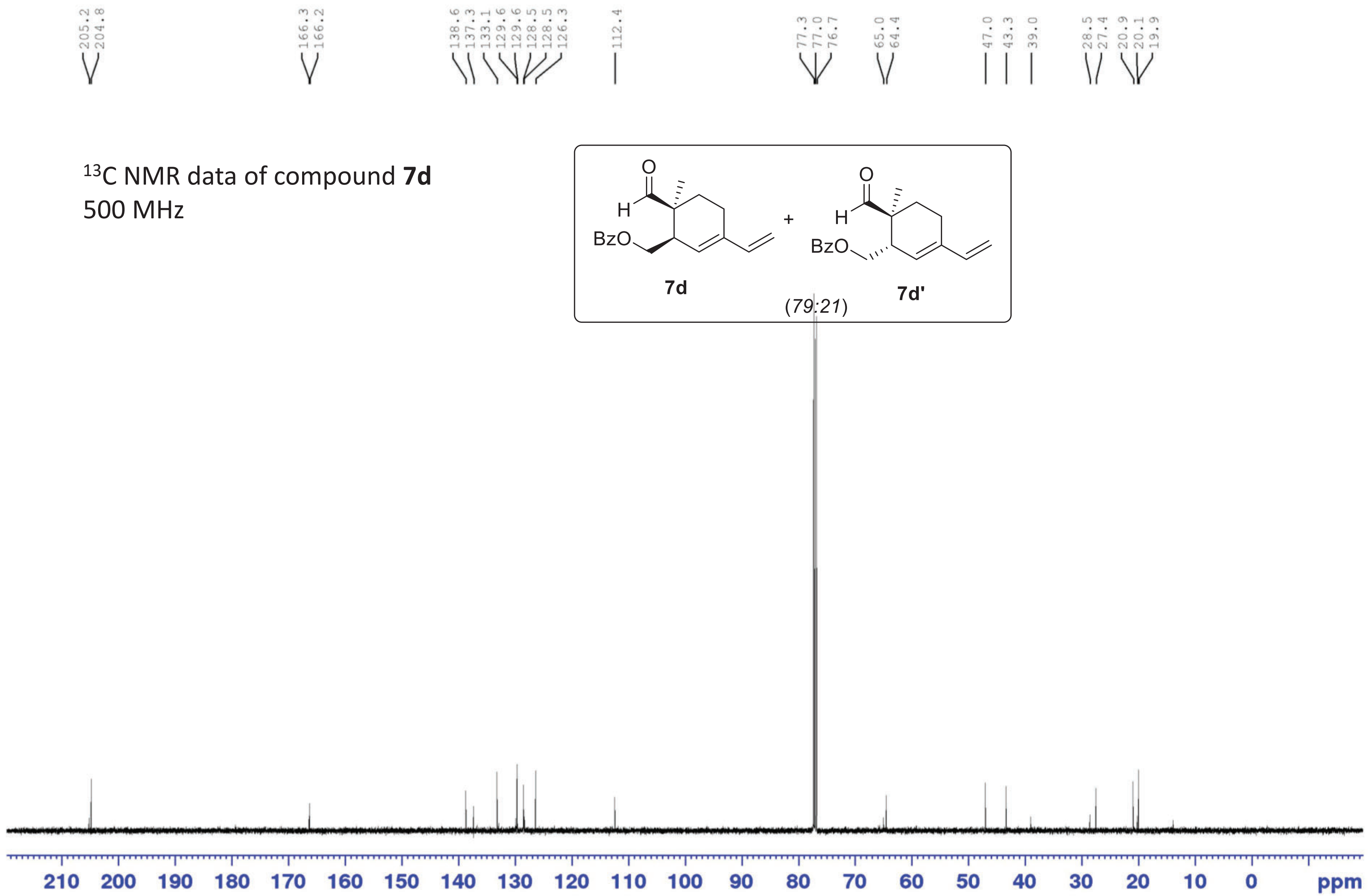

^{1}H NMR data of compound **7e**
500 MHz

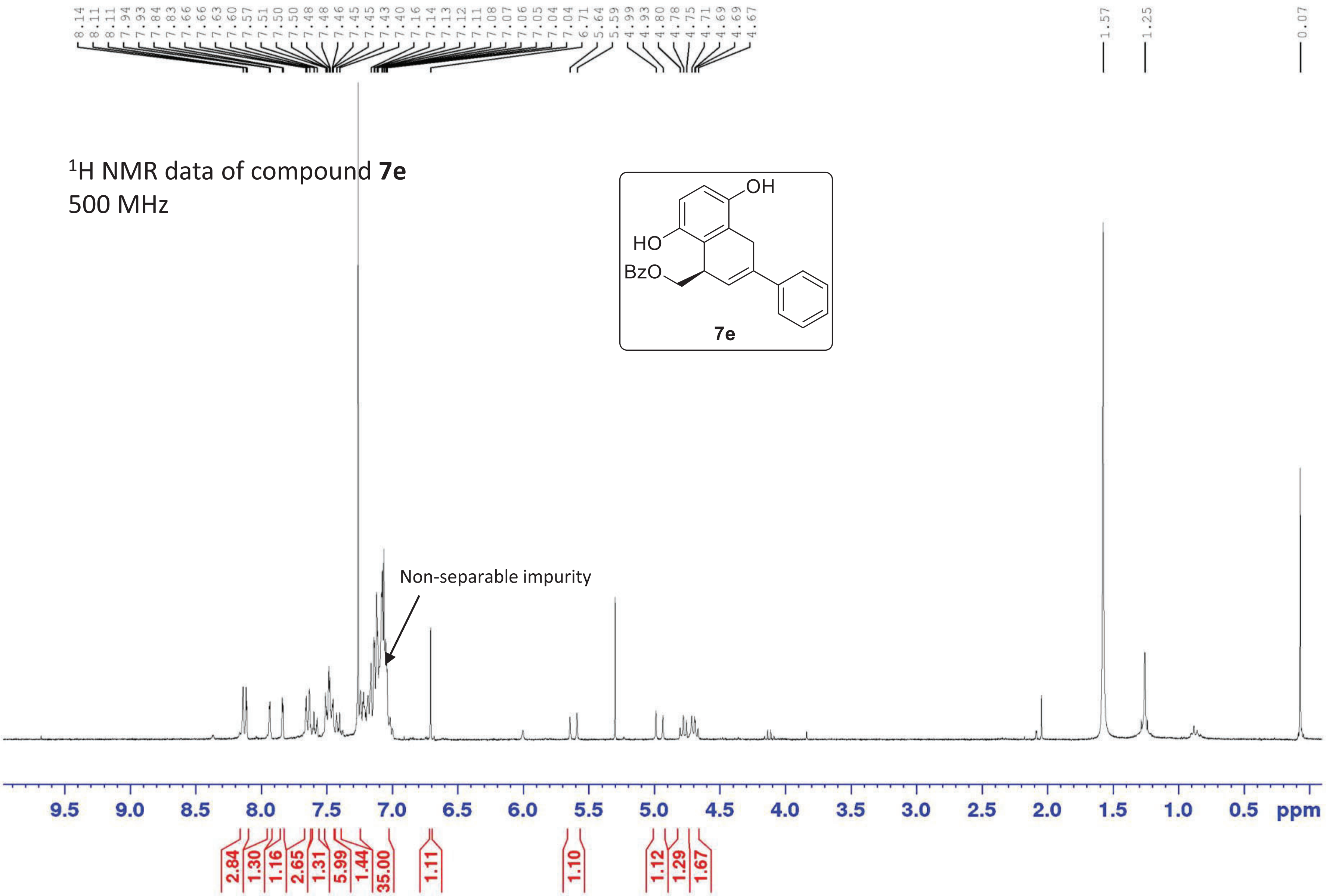

Comparison of DA product and Stille product in one pot for 3g

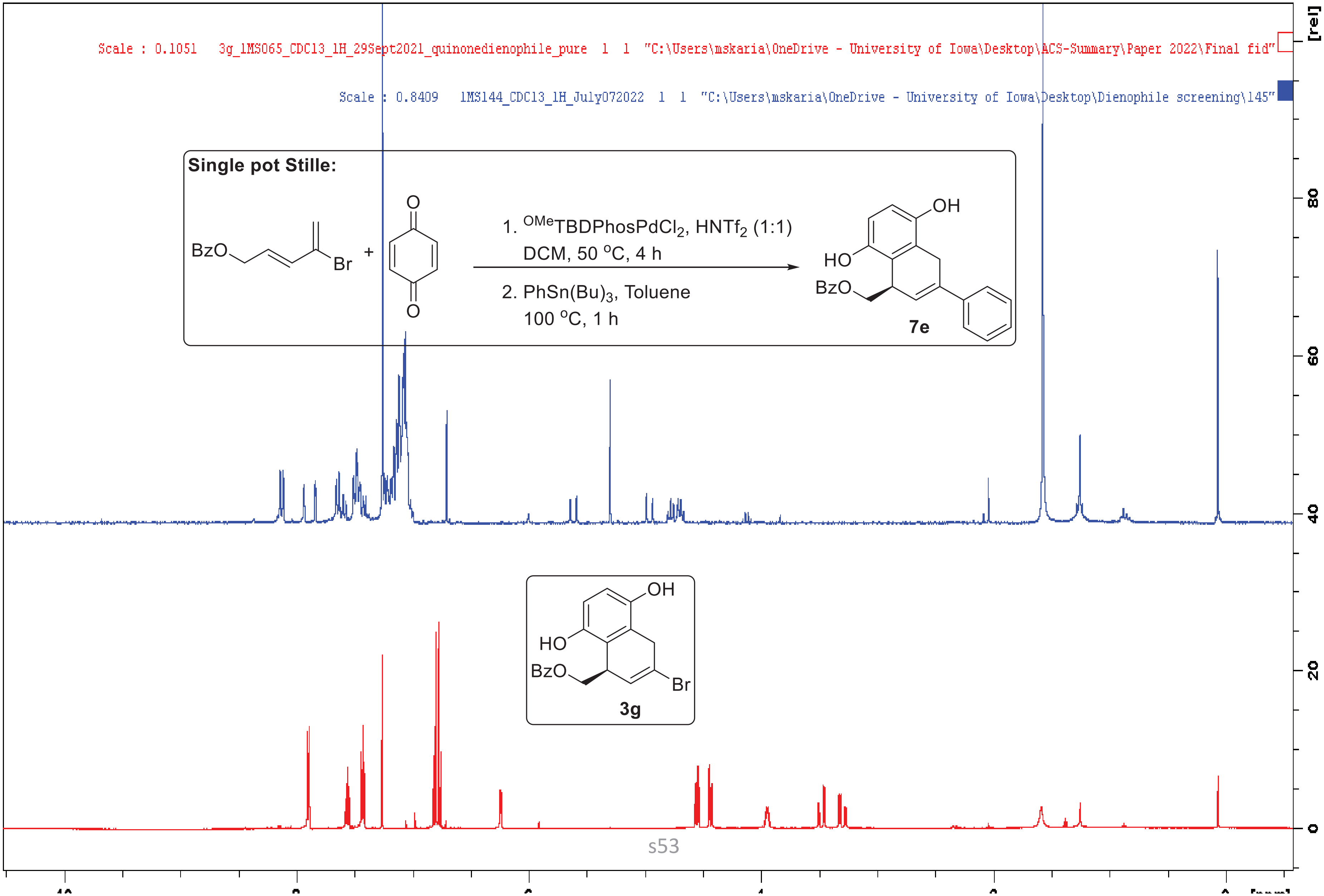

^{1}H NMR data of compound **8a**
500 MHz

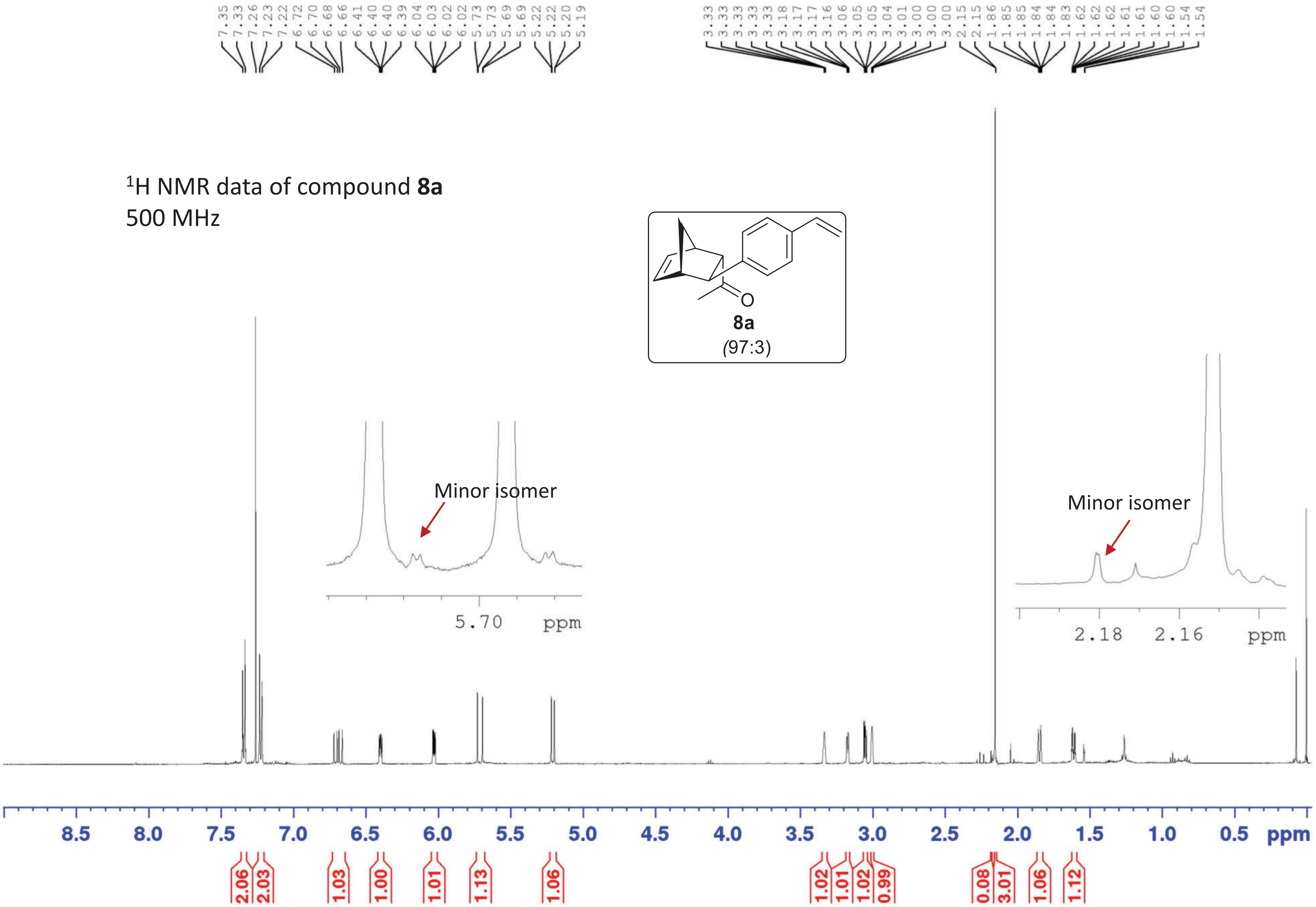

^{13}C NMR data of compound **8a**
500 MHz

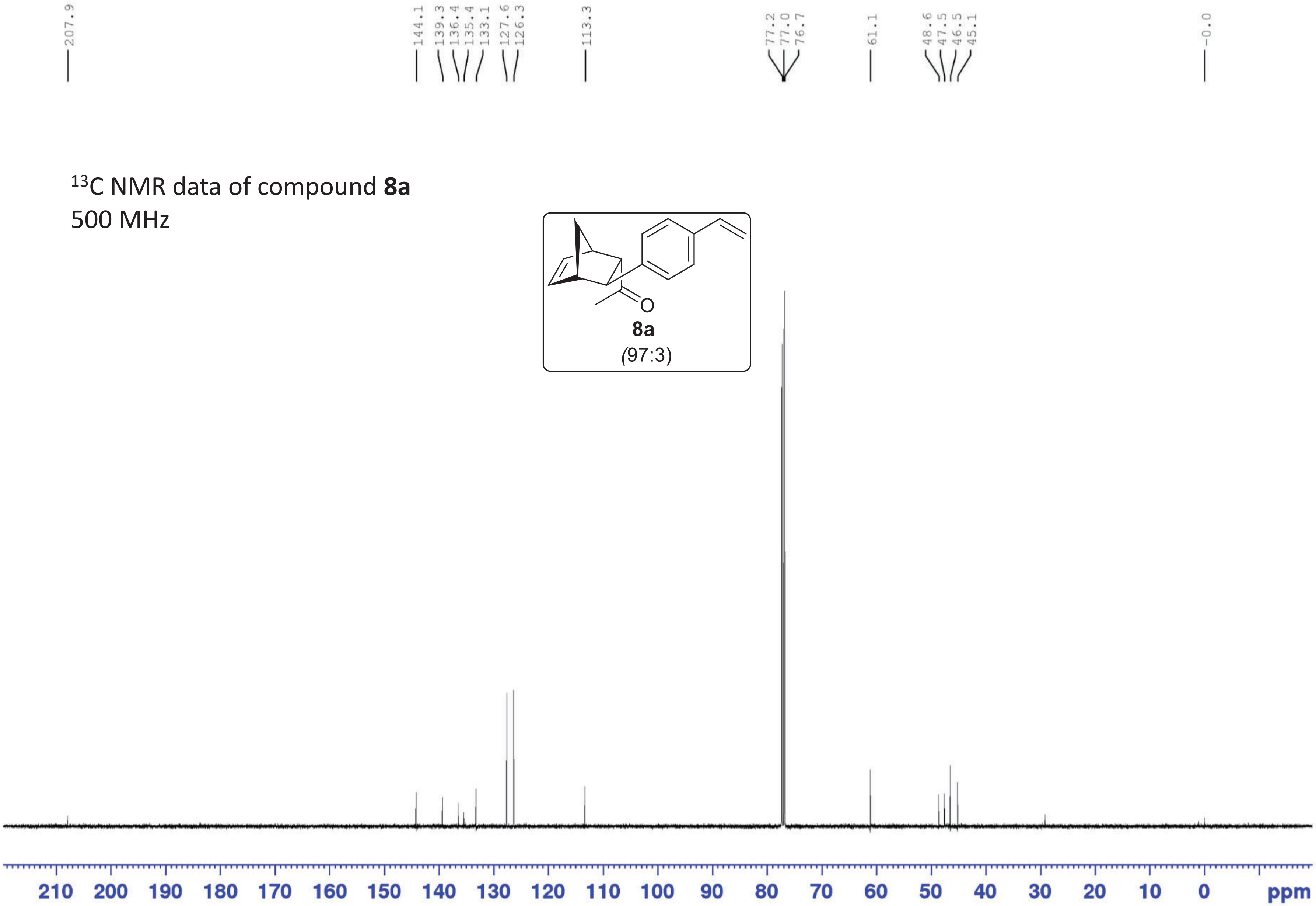

^{1}H NMR data of compound **8b**
500 MHz

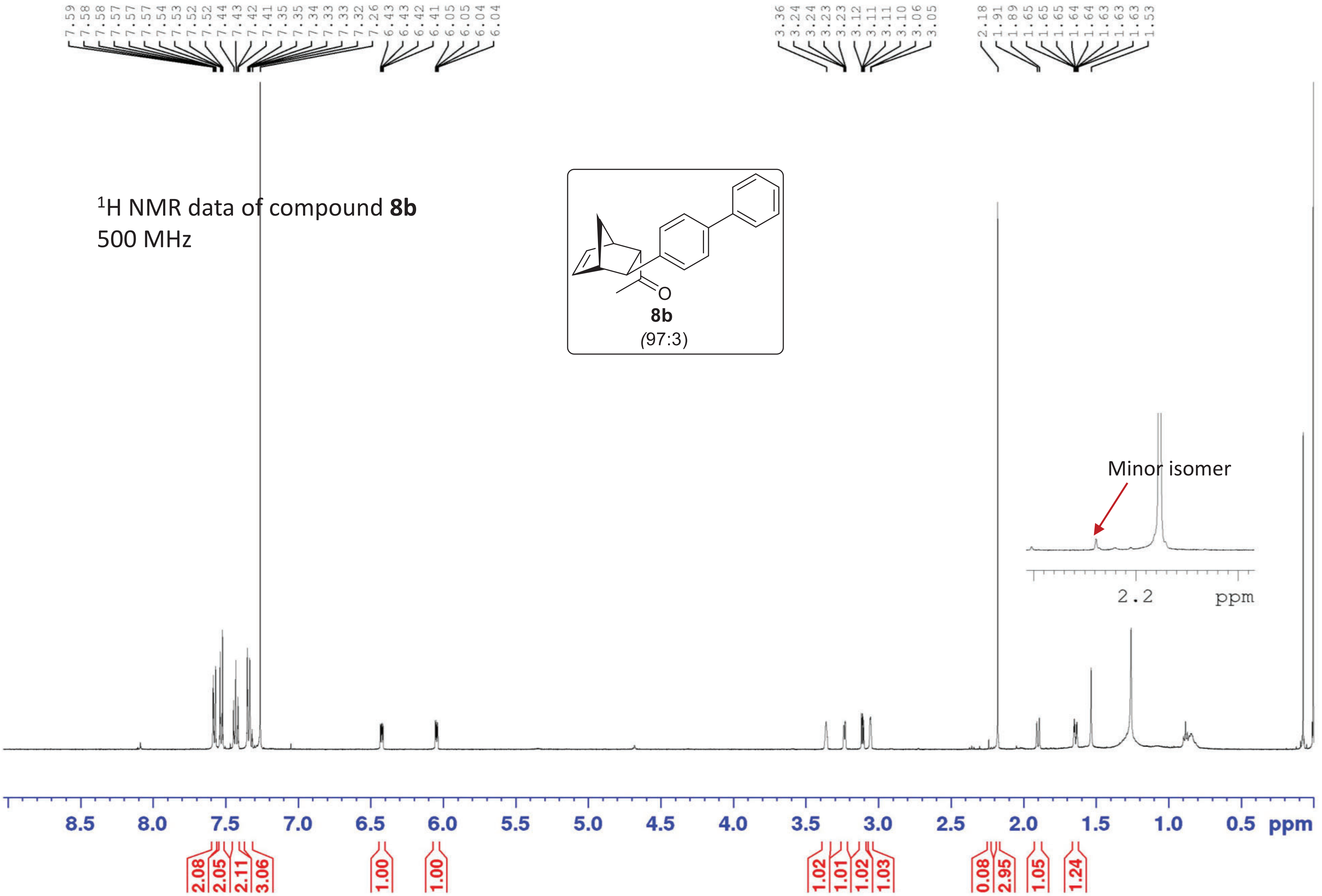

^{13}C NMR data of compound **8b**
500 MHz

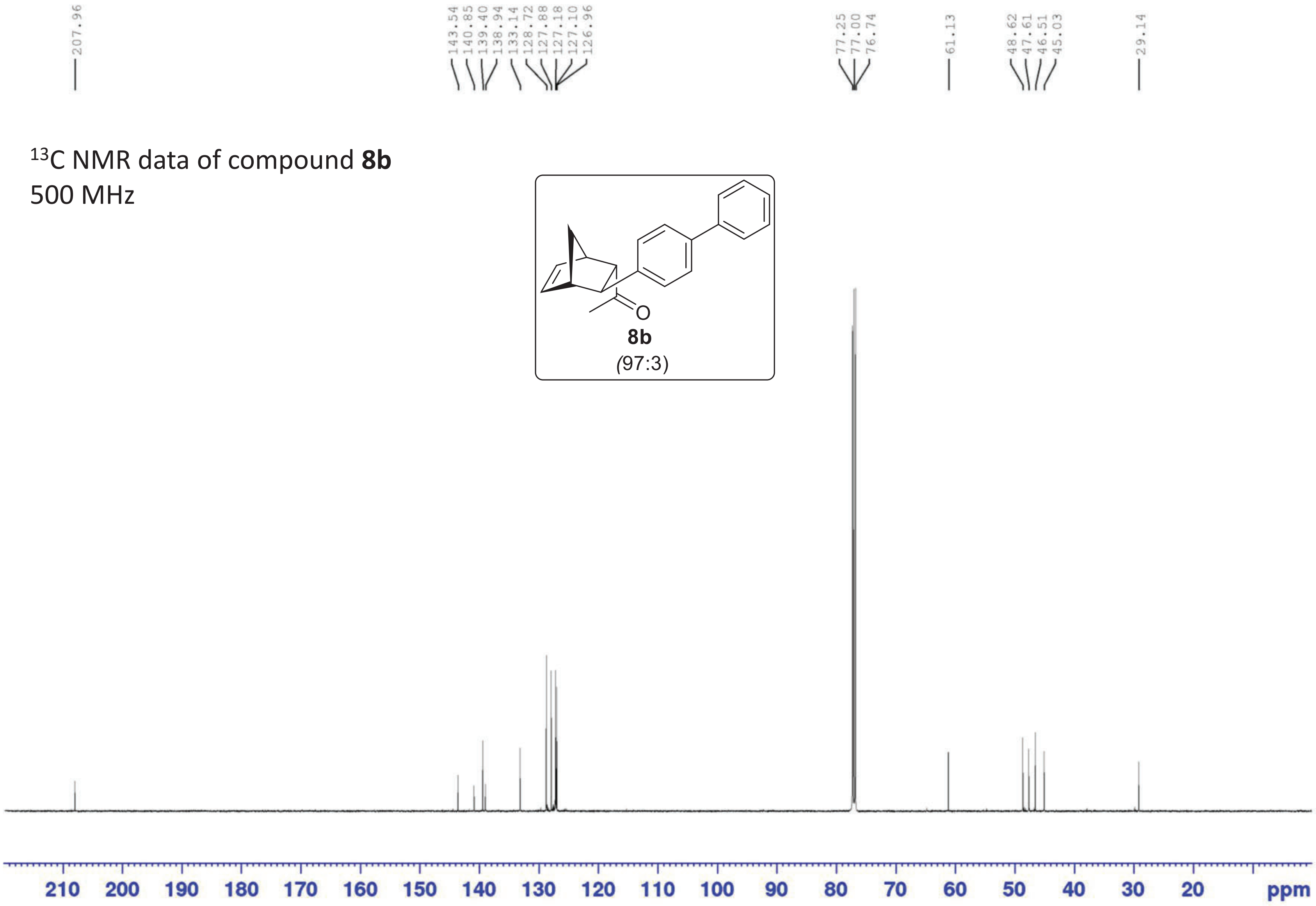

^{1}H NMR data of compound **8c**
500 MHz

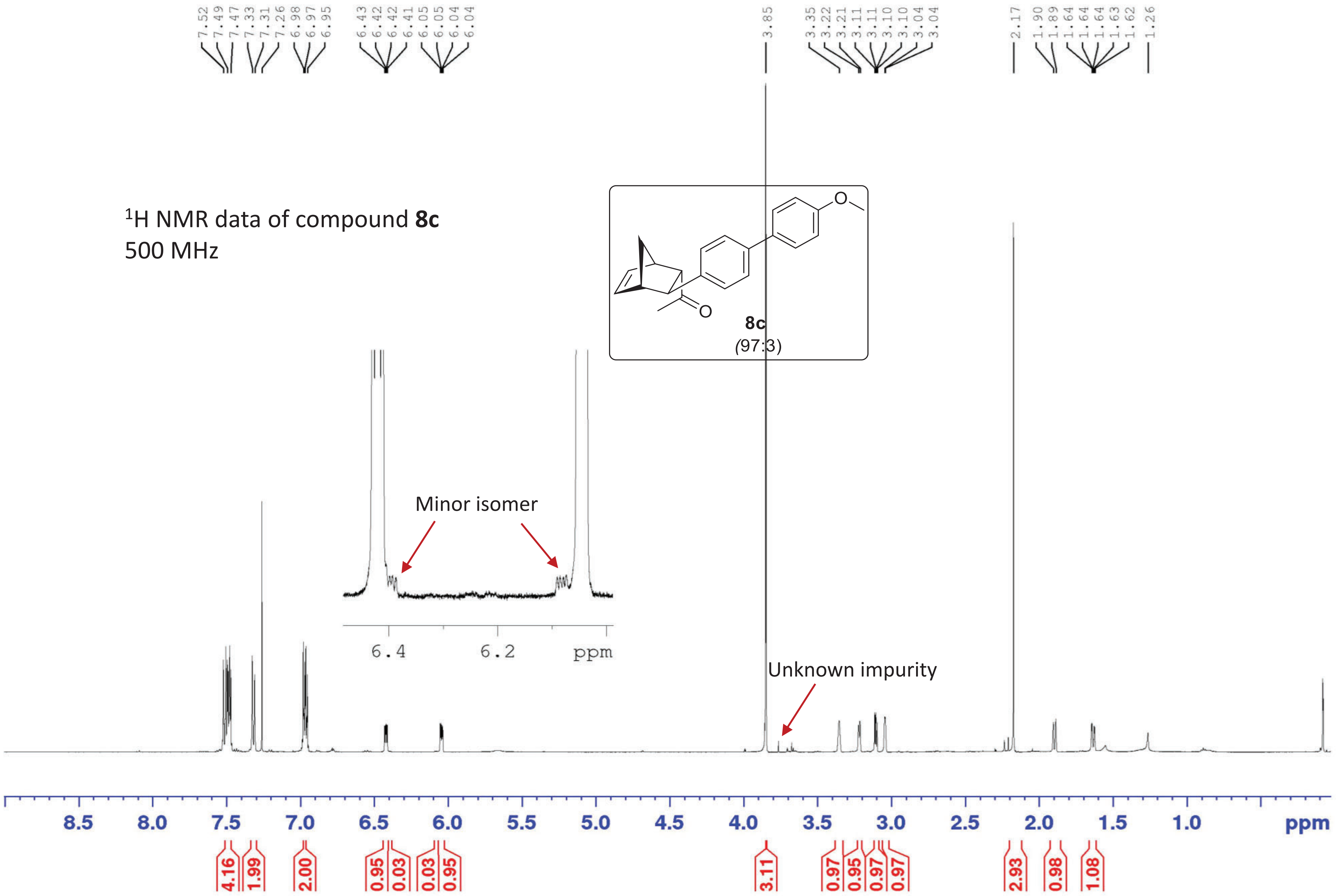

^{13}C NMR data of compound **8c**
500 MHz

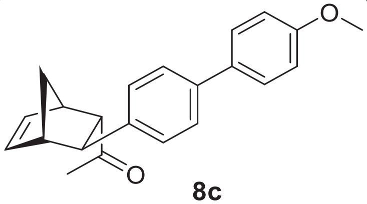

8c
(97:3)

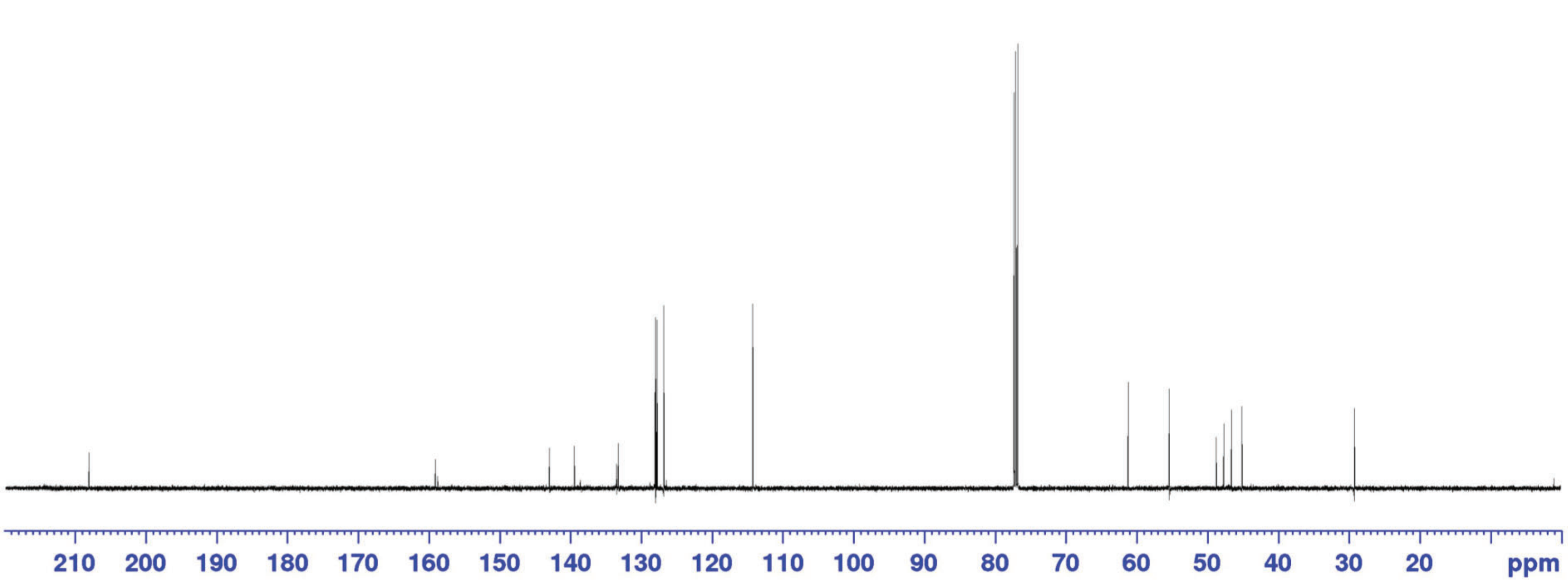

^{1}H NMR data of compound **8d**
500 MHz

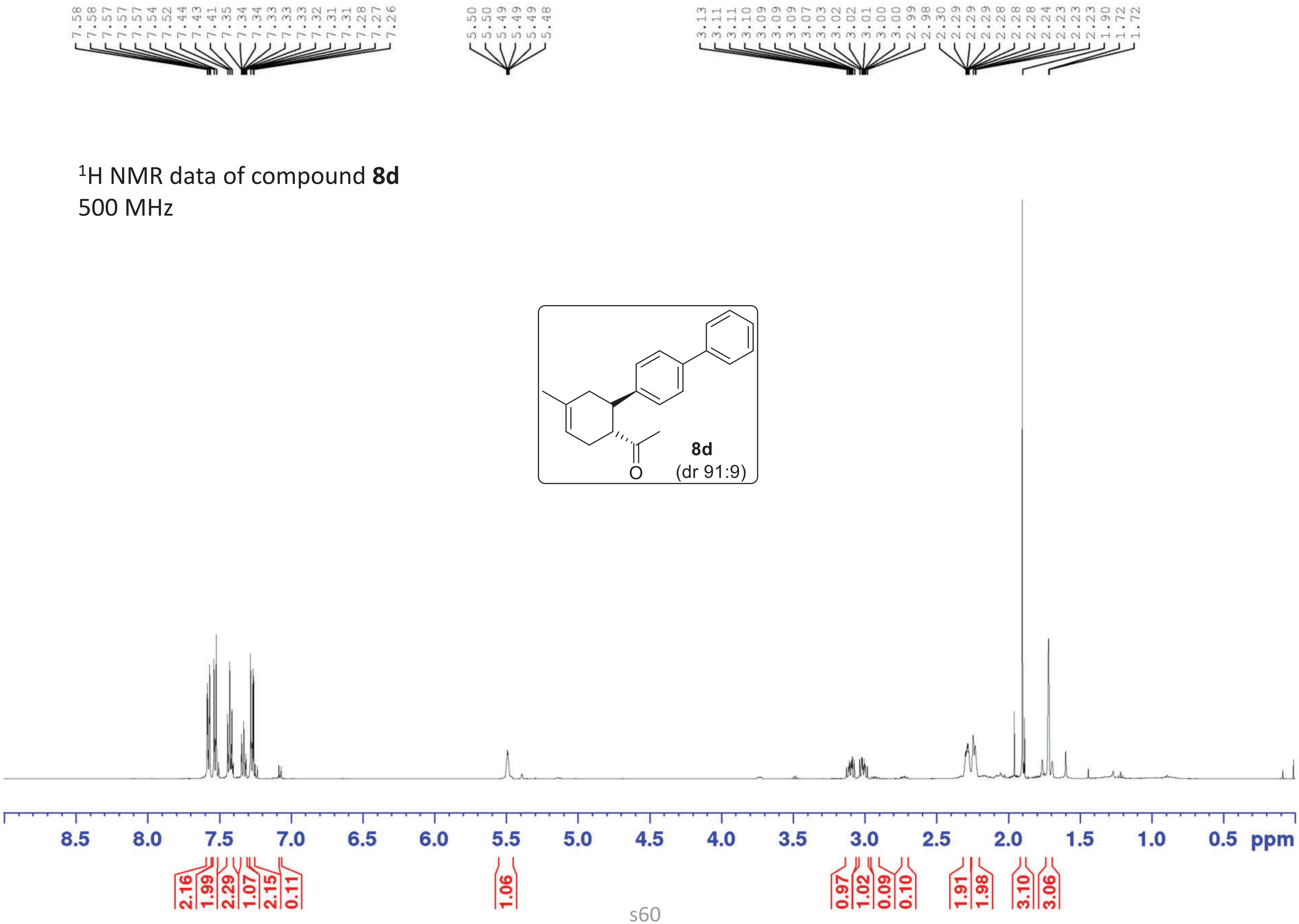

^{13}C NMR data of compound **8d**
500 MHz

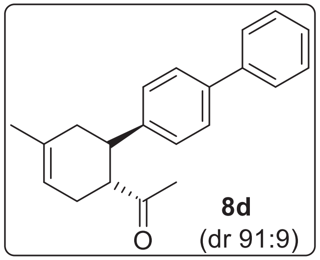

8d
(dr 91:9)

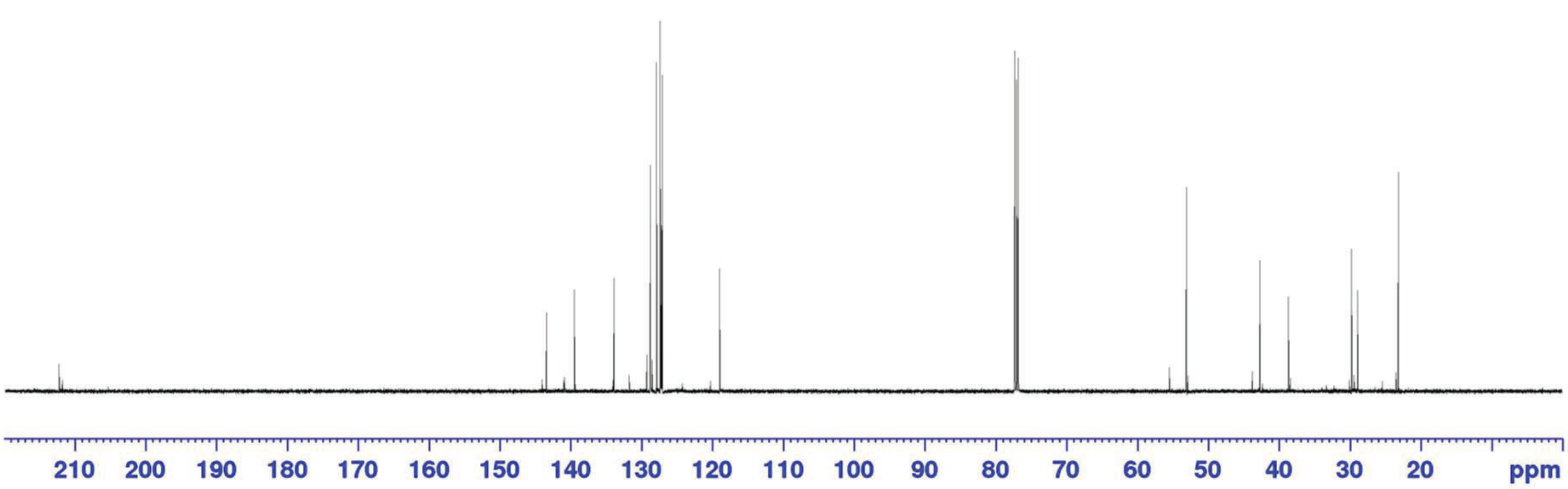

^{1}H NMR data of compound **9a**
500 MHz

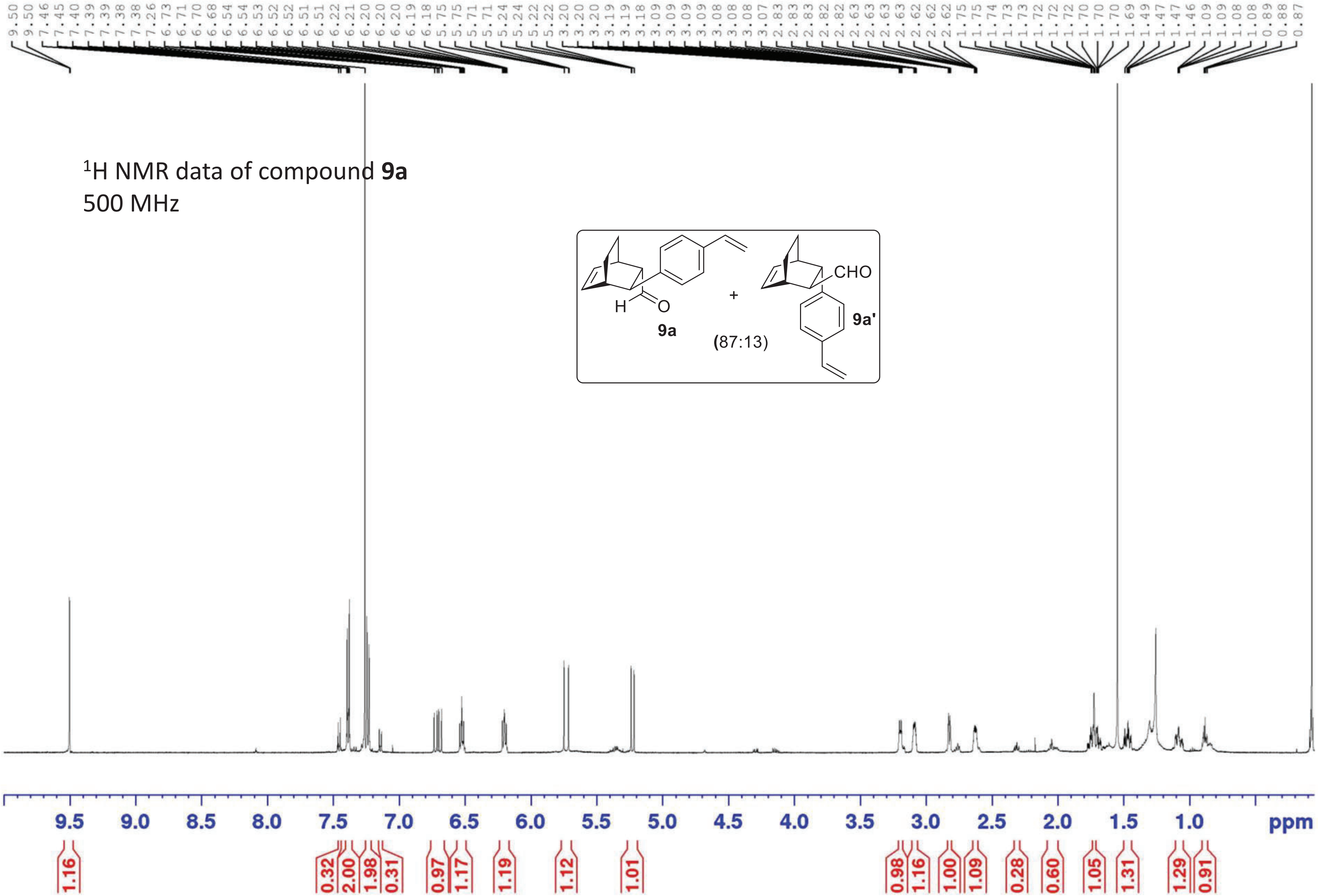

^{13}C NMR data of compound **9a**
500 MHz

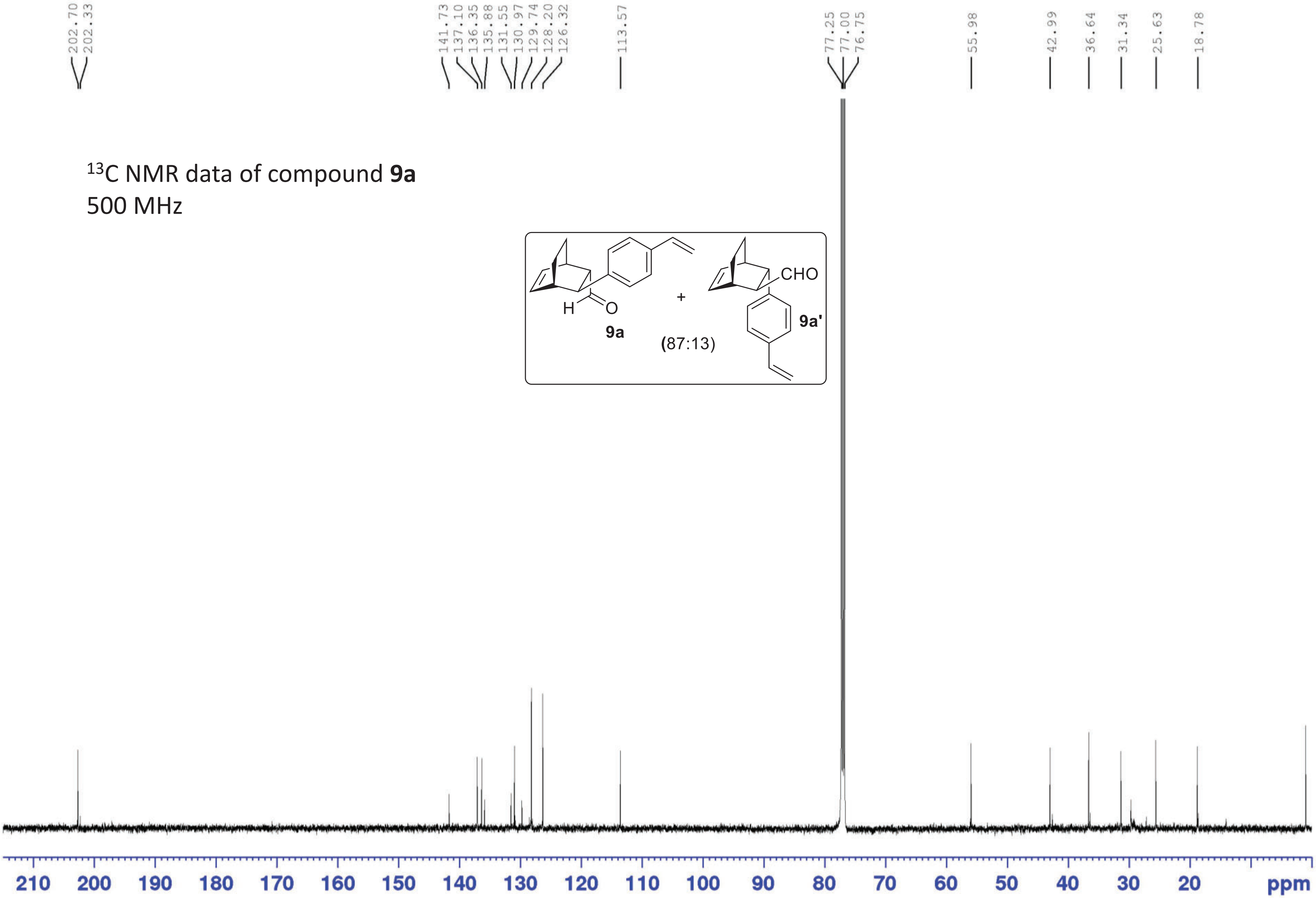

Catalytic Screening- changing substituents on Phosphorous

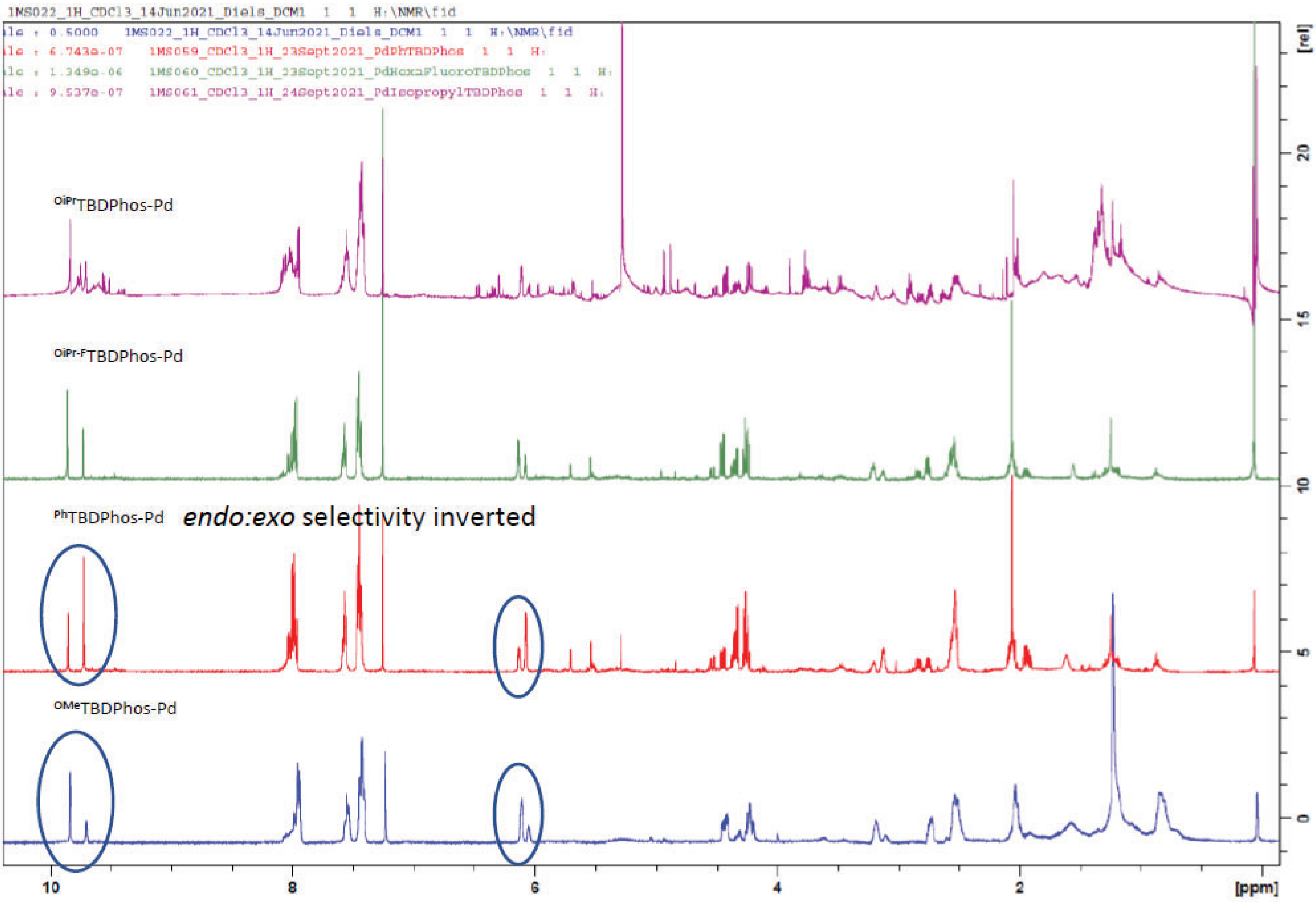

Supplement: Supplementary file 1 — Supporting Information [file CHEM-28-0-s001.pdf]
